# Platinum is essential in neoadjuvant treatment of triple-negative breast cancer: a network meta-analysis

**DOI:** 10.20892/j.issn.2095-3941.2021.0529

**Published:** 2022-02-16

**Authors:** Junjie Li, Li Chen, Wei Tan, Fang Qi, Yang Zhang, Zhonghua Wang, Zhimin Shao

**Affiliations:** 1 Key Laboratory of Breast Cancer in Shanghai, Department of Breast Surgery, Fudan University Shanghai Cancer Center, Shanghai 200032, China; 2 Department of Oncology, Shanghai Medical College, Fudan University, Shanghai 200032, China; 3 Nottingham Clinical Trials Unit, University of Nottingham, Nottingham NG7 2RD, UK; 4 Academic Department, Systematic Review Solutions Ltd, Shanghai 201400, China

**Keywords:** Breast cancer, triple negative, neoadjuvant, network meta-analysis

## Abstract

**Objective:**

This study aimed to assess the efficacy and safety of various neoadjuvant regimens for patients diagnosed with early-stage or locally advanced triple-negative breast cancer (TNBC).

**Methods:**

Medline, EMBASE, Cochrane Library, and Web of Science were searched in May 2020 to identify randomized controlled trials (RCTs). Bayesian network meta-analysis (NMA) was performed (Registration: PROSPERO CRD42020223012).

**Results:**

A total of 35 RCTs involving 8,424 participants were reviewed, of which 22 RCTs with 5,203 patients were included in this NMA focusing on pathologic complete response (pCR). An anthracycline-taxane-based (AT) regimen combined with a platinum (ATPt) [odds ratio (OR) = 2.04, 95% credible interval (CrI): 1.69, 2.48] regimen, and a docetaxel regimen combined with a carboplatin (TCb; OR = 2.16, 95% CrI: 1.20, 3.91) regimen improved pCR beyond that with AT only. AT and ATPt combined with targeted therapy [including bevacizumab (Bev), veliparib, atezolizumab, or pembrolizumab] also improved pCR. Five RCTs included in this NMA reported serious adverse events (SAEs) or grade ≥ 3 AEs. TCb was associated with fewer grade ≥ 3 AEs than was AT (OR = 0.66, 95% CrI: 0.23, 1.72) alone. In contrast, ATPt, AT + Bev, ATPt + Bev, ATPt + veliparib, and ATPt + pembrolizumab were associated with more SAEs than was AT alone.

**Conclusions:**

In patients with TNBC, platinum-based neoadjuvant regimens ATPt and TCb increase pCR beyond that with AT alone, but TCb appears to be better tolerated than either AT or ATPt. Platinum-based regimens combined with targeted therapies (Bev, PARPi, and PD-1/PD-L1 inhibitor) also improve the pCR rate beyond that with AT alone, but this benefit is accompanied by greater toxicity.

## Introduction

Triple-negative breast cancer (TNBC) is defined by cancer cells that lack estrogen receptors, progesterone receptors, and human epidermal growth factor receptor type 2 (HER2) expression. TNBC is a genetically heterogeneous, aggressive molecular subgroup of breast cancer (BC) that accounts for approximately 15%–20% of all BCs and often occurs in younger women^1^. Although many studies have been conducted on TNBC, its prognosis remains poor in the long term. Approximately 25%–30% of patients with early-stage TNBC are estimated to develop distant metastases within 3–5 years after diagnosis^2^. Although adjuvant therapy remains commonly used, neoadjuvant chemotherapy is now recognized as the standard of care for patients with TNBC^3,4^.

Neoadjuvant therapy, consisting of systemic therapy before surgical tumor removal, can downstage tumors, thus allowing for breast-conserving surgery and offering a valuable opportunity to monitor individual tumor responses^1,5,6^. Pathologic complete response (pCR) is used to interpret prognostic information, predict overall outcomes, and guide adjuvant therapy selection and decision-making^2,7^. Minckwitz et al.^8^ have reported a pooled analysis exploring the association between pCR and long-term clinical benefits in TNBC. The results indicated that patients who achieve a pCR have significantly better event-free survival and overall survival outcomes than those who do not; however, a similar difference was not observed in hormone receptor (HP)-positive patients. Achieving a pCR is thus highly prognostic in TNBC, because such patients have better survival in the long term. Although the Create-X^9^ study has recently shown that adding the adjuvant capecitabine after standard neoadjuvant chemotherapy prolongs overall survival in patients with TNBC with residual invasive disease on pathological testing, gaps exist in the medical knowledge regarding how best to increase the pCR rate for TNBC. Therefore, more individualized therapy strategies are needed for patients without pCR.

Several studies combining standard neoadjuvant regimens with platinum or targeted agents, such as bevacizumab (Bev), PARP inhibitors (PARPi), and PD-1/PD-L1 inhibitors, have been shown to improve pCR rates in TNBC^3,4,10^.

Most drugs used in neoadjuvant regimens can cause serious adverse effects (AEs) that may lead to poorer prognosis or death. Several studies have reported that participants withdrew or discontinued treatment because of severe toxicity^11^. Common AEs of neoadjuvant regimens include thrombocytopenia, neutropenia, anemia, myelogenous leukemia, alopecia, stomatitis, anorexia, pyrexia, conjunctivitis, cardiac disorder, and pigmentation^12^.

Because of the heterogeneity of TNBC and the variety of neoadjuvant regimens, finding the optimal neoadjuvant regimen to improve long-term outcomes in patients with early-stage TNBC remains a challenge in clinical practice. A previous meta-analysis^13^ has shown that a platinum-based regimen may be an option in the neoadjuvant setting; however, the regimen providing the best benefit/risk ratio when combined with targeted agents such as Bev, PARPi, and PD-1/PD-L1 inhibitors remains unknown. The toxicity of neoadjuvant regimens may be a barrier for clinicians, who might prefer to select better tolerated agents and dosages for patients with TNBC. To help clinicians choose appropriate treatments for patients with TNBC, we conducted a network meta-analysis (NMA) to assess the efficacy and safety of various neoadjuvant regimens for patients diagnosed with early-stage or locally advanced TNBC.

## Materials and methods

This study was registered with the International Prospective Register of Systematic Reviews (PROSPERO; registration number CRD42020223012)^14^. The study was conducted according to the Preferred Reporting Items for Systematic Reviews and Meta-Analyses (PRISMA)-NMA checklist^15^.

### Search strategy and selection criteria

Medline, EMBASE, Cochrane Library, and Web of Science were searched from inception to September 2020, without limitations on the date/time, language, or document type. The reference lists of the included studies were examined to identify any additional relevant published or unpublished material not retrieved by the electronic search. Search strategies for all databases are described in detail in Online Appendix 1.

Randomized controlled trials (RCTs) fulfilling the following criteria were included: 1) patients with early or locally advanced TNBC (clinical stage of I–III or M0); 2) any neoadjuvant regimen (concurrent or sequential chemotherapy) including a single drug or a combination of any of the following drugs: paclitaxel, docetaxel, platinum/cisplatin/carboplatin/oxaliplatin, albumin paclitaxel, capecitabine/gemcitabine/5-fluorouracil, doxorubicin/epirubicin, cyclophosphamide, pembrolizumab/nivolumab/atezolizumab, veliparib/olaparib, or everolimus; and 3) any outcomes of interest, namely pCR (ypT0/is ypN0 or ypT0 ypN0), serious AEs (SAEs), or grade ≥ 3 AEs.

Patients in studies with subgroup analysis of TNBC were included only if they were stratified according to receptor status when randomized. Randomized controlled trials (RCTs) published only as abstracts without full articles or detailed reports were excluded from the analysis. Studies in a language other than English were excluded.

### Screening, data extraction, and assessment of risk of bias

Four reviewers were divided into 2 groups to independently screen the articles (JL and LC; FQ and YZ), perform data extraction (JL and LC; FQ and YZ), and assess the risk of bias (JL and LC; WT and FQ). Disagreements were resolved by discussion, with assistance from a third party (ZW or ZS) if necessary. The 7 domains of the Cochrane Risk of Bias tool^16^ were evaluated, comprising sequence generation, allocation concealment, blinding of participants and personnel, blinding of outcome assessment, incomplete outcome data, selective outcome reporting, and other bias. More details have been presented in our protocol^14^.

### Statistical analysis

The primary objective was to compare pCR among all included RCT regimens in the network. The second objective was to compare aggregated AEs (defined as total SAEs or grade ≥3 AEs, owing to different AE reports in the RCTs) among all included RCT regimens in the network. A fixed-effect NMA within a Bayesian framework was performed in R 3.6.2 software (gemtc package)^17^. The pooled estimation and the probability of a given drug being optimal were obtained according to the Markov chain Monte Carlo method. The model convergence was assessed with trace plots and Brooks-Gelman-Rubin plots^18^. The results of dichotomous outcomes are reported as odds ratios (ORs) and credible intervals (CrIs). The ranking probabilities for all neoadjuvant regimens were estimated and are reported as the area under the cumulative ranking curve (SUCRA). Evidence inconsistency and clinical similarities in patient characteristics and settings across trials were carefully assessed before analysis. Network geometry was performed in STATA 16.0 software.

## Results

### Results of the search

**Figure 1:**
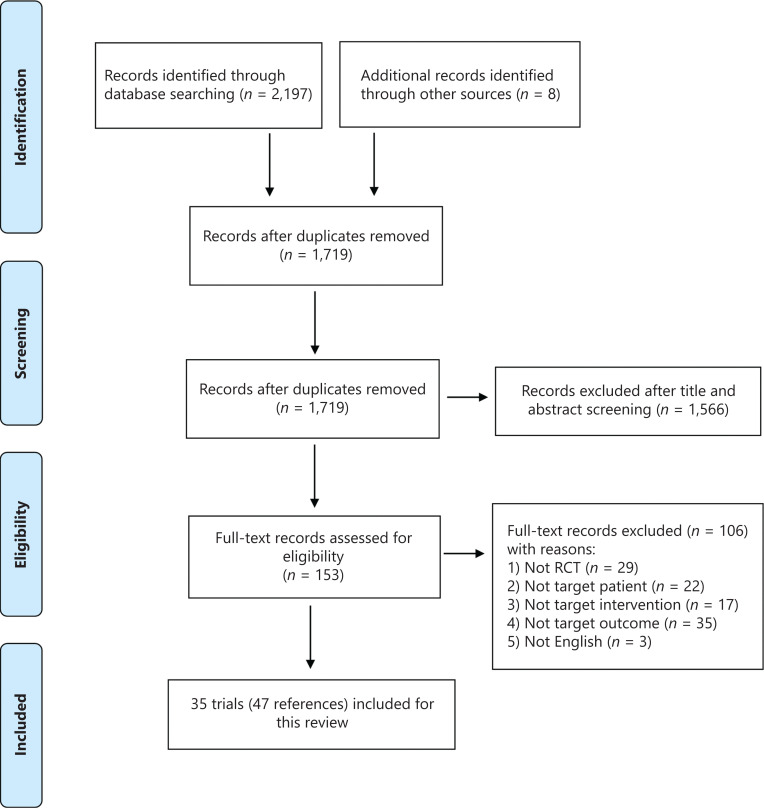
PRISMA flow diagram.

A total of 2,205 articles were identified [2,197 articles identified through an electronic database search in August 2020, and 8 articles identified from abstracts and posters for the American Society of Clinical Oncology (ASCO) and the European Society for Medical Oncology (ESMO) annual meetings, and the San Antonio Breast Cancer Symposium (SABCS)]. After removal of duplicates, 1,719 articles were identified for screening. An additional 1,566 articles were excluded after inspection of the titles and abstracts. The remaining 153 articles were read in full, and 106 articles were subsequently excluded for various reasons (further details in **Figure 1**). Thirty-five RCTs (with 47 references) were eligible according to the inclusion criteria; of these, 22 RCTs (with 29 references) were included in the NMA.

### Characteristics of the included studies

A total of 35 RCTs (published from 2012 to 2020) involving 8,424 participants met the inclusion criteria for this review. A total of 28 RCTs (80%) were multicenter trials. Participants were recruited from South America (Brazil, Columbia), Mexico, the United States, Canada, Australia, Europe (including Belgium, Czechia, France, Germany, Hungary, Ireland, Italy, the Netherlands, Poland, Portugal, Russia, Spain, Sweden, Turkey, and the United Kingdom), and Asia (including China, India, Israel, Japan, Korea, and Singapore). The average age of the included participants was approximately 50 years. **Table 1** (Online Appendix 4) and Online Appendix 2 (**Supplementary Tables S1** and **S2**) provide more details on the study and population characteristics.

**Table 1:** Characteristics of the included RCTs

Study ID	Country	Center	Sample size at randomization	Clinical stage	Mean age (years)	BRCA (BRCA-1 or BRCA-2) mutation	Direct comparisons	Outcomes reported	Data extracted from subgroup analysis of RCT	Data in network meta-analysis (NMA)	Comparisons in NMA
Alba 2012	Spain	Multi	94	Non-metastatic (non-specific)	47 (median)	NR	EC-T *vs.* EC-TCb	pCR; AE (grade 3–4)	No	Yes	AT *vs.* ATPt
Ando 2014	Japan	Multi	75	II–IIIA	NR	NR	PCb-FEC *vs.* P-FEC	pCR	Yes	Yes	AT *vs.* ATPt
Bear 2012	USA (Puerto Rico), Canada, India	Multi	490	T1c-T3; N0-N2a; M0	NR	NR	T/TX/TG-AC + Bev *vs.* T/TX/TG-AC	pCR	Yes	No	NA
Chen 2016	China	Multi	102	IIB or III	NR	NR	TC *vs.* TAC	pCR	Yes	Yes	AT *vs.* TC
Earl 2015	UK	Multi	248	Early stage (non-specific)	NR	NR	T-FEC *vs.* T-FEC + Bev	pCR	Yes	Yes	AT *vs.* AT + Bev
Fasching 2019	Germany	Multi	77	Early stage (non-specific)	NR	NR	P-EC + Ola *vs.* PCb-EC	pCR	Yes	Yes	AT + Ola *vs.* ATPt
Gerber 2013	Germany	Multi	678	Untreated cT1c-T4d	48	18.3%BRCA1 mutation 15.1%BRCA2 mutation 3.2%	EC-T + Bev *vs.* EC-T	pCR	Yes	Yes	AT *vs.* AT + Bev
Geyer 2017	USA, Australia, Belgium, Canada, Czechia, France, Germany, Hungary, Italy, Korea, Netherlands, Russia, Spain, China (Taiwan), UK	Multi	634	Early stage (non-specific)	50 (median)	NR	PCb-AC + Veli *vs.* PCb-AC *vs.* P-AC	pCR; AE (grade 3–4)	No	Yes	AT *vs.* ATPt *vs.* ATPt + Veli
Gianni 2018	Australia, Germany, Italy, Russia, Singapore, Spain	Multi	219	T2N01; T3N0; T3N1; T4 any N; any T N2-3	NR	NR	P-AC/EC/FEC *vs.* nabP-AC/EC/FEC	pCR	Yes	No	NA
Gigolaeva 2019	Russia	NR	192	IIB–IIIA	47 (median)	BRCA1 mutation 12.0%	AC-P *vs.* AC-q3w PCb/EriCb	pCR	No	Yes	AT *vs.* ATPt
Gluz 2018	Germany	Multi	336	I–IV (IV-1.4%)	50	NR	q3w nabPG *vs.* q3w nabPCb	pCR; SAE	No	No	NA
Gonzalez-Angulo 2014	Germany	Single	62	IIA–IIIC	48	NR	P-FEC *vs.* P-FEC + Eve	pCR	No	Yes	AT *vs.* AT + Eve
Harbeck 2020	USA, Australia, Belgium, Brazil, Canada, Germany, Italy, Japan, Korea, Poland, Spain, China (Taiwan), UK	Multi	333	II–III	NR	NR	nabP-AC + Atezo *vs.* nabP-AC	pCR; AE (grade 3–5)	No	Yes	AT *vs.* AT + Atezo
Ishikawa 2016	Japan	Single	66	I–IIIC	53	NR	TC *vs.* FEC-T	pCR	Yes	Yes	AT *vs.* TC
Jovanovic 2017	USA	Multi	145	II or III	52	4.0%	PCis + Eve *vs.* PCis	pCR; AE (grade 3–5)	No	No	NA
Kummel 2017	Germany	Multi	131	cT2-T3	NR	NR	Caba *vs.* P	pCR	Yes	No	NA
Llombart-Cussac 2015	France, Germany, Spain	Multi	141	II–IIIA	NR	NR	P *vs.* P + weekly Ini *vs.* P + q2w Ini	pCR; treatment-related AE (grade 3–4)	No	No	NA
Loi 2019†	UK	Multi	60	Early stage (non-specific)	48.5 (median)	NR	nabP-AC + Pembro *vs.* nabPCb-AC + Pembro *vs.* PCb-AC + Pembro	pCR; SAE	No	No	NA
Loibl 2018	USA, Australia, Belgium, Canada, Czechia, France, Germany, Hungary, Italy, Korea, Netherlands, Russian, Spain, China (Taiwan), UK	Multi	634	II–III	50	Deleterious mutation 14.7%	PCb-AC + Veli *vs.* PCb-AC *vs.* P-AC	pCR; AE (grade 3–4)	No	Yes	AT *vs.* ATPt *vs.* ATPt + Veli
Loibl 2019	Germany	Multi	174	Early stage (non-specific)	49.5	NR	nabP-AC + Durva *vs.* nabP-AC	pCR; SAE	No	Yes	AT *vs.* AT + Durva
Martinez 2015	Mexico	NR	61	Locally advanced (non-specific)	47 (median)	NR	P-FAC *vs.* PA + Cis	pCR	No	Yes	AT *vs.* ATPt
Mayer 2019	USA	NR	140	I–III	NR	NR	Cis *vs.* P	pCR	No	No	NA
Nahleh 2016	USA (Puerto Rico), India	Multi	67	IIB–IIIC	NR	NR	nabP-AC + Bev *vs.* AC-nabP	pCR	Yes	Yes	AT *vs.* AT + Bev
Nanda 2020	USA	Multi	88	II–III	NR	NR	P-AC *vs.* P-AC + Pembro	pCR	Yes	Yes	AT *vs.* AT + Pembro
Rugo 2016	USA	Multi	60	II–III	NR	NR	P-AC *vs.* PCb-AC + Veli	pCR	Yes	Yes	AT *vs.* ATPt + Veli
Schmid 2020	USA, Australia, Brazil, Canada, Columbia, France, Germany, Ireland, Israel, Italy, Japan, Korea, Poland, Portugal, Russia, Singapore, Spain, Sweden, China (Taiwan), Turkey, UK	Multi	1174	II–III	NR	NR	PCb-AC/EC + Pembro *vs.* PCb-AC/EC	pCR; AE (grade ≥ 3)	No	Yes	ATPt *vs.* ATPt + Pembro
Schneeweiss 2019	Germany	Multi	403	Early stage (non-specific)	NR	NR	AC-q2wP *vs.* PA + Cb	pCR	Yes	Yes	AT *vs.* ATPt
Sharma 2019	USA	Multi	100	I–III	52 (median)	17.0%	PCb-AC *vs.* TCb	pCR; AE (grade 3–4)	No	Yes	ATPt *vs.* TCb
Sikov 2015	USA	Multi	454	II–III	NR	NR	P-AC *vs.* P-AC + Bev *vs.* PCb-AC *vs.* PCb-AC + Bev	pCR; SAE	No	Yes	AT *vs.* ATPt *vs.* AT + Bev *vs.* ATPt + Bev
Tung 2020	USA	Multi	83	I–III	NR	NR	Cis *vs.* AC	pCR	Yes	No	NA
Untch 2016	Germany	Multi	276	Early stage (non-specific)	NR	NR	nabP-EC *vs.* P-EC	pCR	Yes	No	NA
Von Minckwitz 2014	Germany	Multi	315	II–III	NR	15.9%	PACb + Bev *vs.* PA + Bev	pCR	No	No	NA
Wu 2018	China	Single	128	I–III	47 (median)	NR	ET *vs.* ET + Loba	pCR	No	No	NA
Zhang 2016	China	Single	91	II–III	47 (median)	NR	q3w PCb *vs.* q3w PE	pCR	No	No	NA
Zhang 2020	USA	Multi	93	Early stage (non-specific)	49 (median)	Deleterious mutation 12.2%	TCb *vs.* EC-T	pCR	No	Yes	AT *vs.* TCb

A, doxorubicin; SAE, serious adverse event; Atezo, atezolizumab; Bev, bevacizumab; BRCA mutation, mutations in 2 genes producing a hereditary breast-ovarian cancer syndrome; BRCA1, the first of these genes to be discovered; BRCA2, the second of these genes to be discovered; C, cyclophosphamide; Caba, cabazitaxel; Cb, carboplatin; Cis, cisplatin; Durva, durvalumab; E, epirubicin; Eve, everolimus; F, 5-fluorouracil; G, gemcitabine; Ini, iniparib; Loba, lobaplatin; nabP, albumin paclitaxel (weekly cycle if not specifically noted); NR, not reported; Ola, olaparib; P (weekly cycle if not specifically noted); Pt, platinum; pCR, pathologic complete response; Pembro, pembrolizumab; q2/3w: every 2/3 weeks; T, docetaxel; X, capecitabine. According to previous reports, guidelines, and clinical practice, a reasonable combination was made to maximize the inclusion of RCTs in NMA, which included the following: doxorubicin and epirubicin regarded as equal, cisplatin and carboplatin regarded as equal, TAC and AC-T regarded as equal, different sequential sequences regarded as equal (such as AC-P equal to P-AC, etc.). Citations for included RCTs are presented in Online Appendix 4. We excluded studies with interventions in only one study from this network meta-analysis (NMA). †Loi 2019 is a phase Ib study with 6 treatment arms exploring doses for chemotherapy combined with pembrolizumab, whose objective was not the primary focus in this NMA; each arm enrolled only 10 participants. We excluded this study from the outcome description and primary NMA analysis. In network meta-analysis, regimens including FECT, P-FAC, ACT, AC-nabP, and ACP were merged as anthracycline-taxane based (AT) regimens, and regimens including EC-TCb, PA + Cis/Cb, PCb-FEC, and PCb-AC were merged as anthracycline-taxane based + platinum (ATPt) regimens. (Sensitivity analyses were also performed on the basis of detailed regimens; details in Online Appendix 7: **Supplementary Figures S7–S11** and **Supplementary Tables S17–S22** and Online Appendix 8: **Supplementary Figures S12–S15** and **Supplementary Tables S23–S28**).

All included RCTs reported pCR outcomes (neoadjuvant regimens in 22 RCTs were connected for NMA); 11 RCTs reported SAEs or grade ≥ 3 AEs (neoadjuvant regimens in 5 RCTs were connected for NMA). Data were extracted from subgroup analyses for TNBC in 15 RCTs. More details are presented in Online Appendix 2 (**Supplementary Tables S3** and **S4**). A detailed risk of bias assessment is reported in Online Appendix 3 (**Supplementary Figure S1**).

### Effects of interventions (pCR)

**Figure 2:**
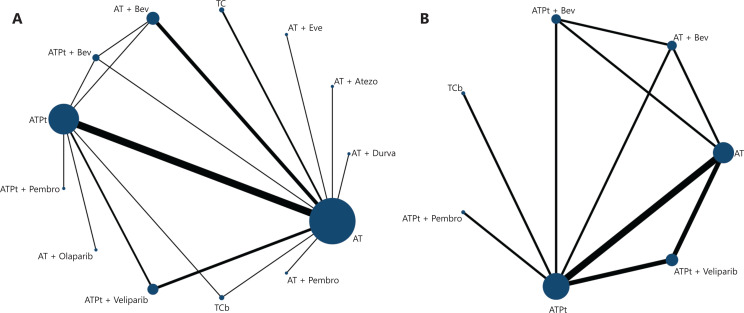
Network structure (A, pCR; B, aggregated AEs). Notes: Direct comparisons are represented by the black lines connecting the neoadjuvant therapy regimens. Line width is proportional to the number of trials including every pair of neoadjuvant regimens, whereas circle size is proportional to the total number of trials for each neoadjuvant regimen in the network. A, doxorubicin; Atezo, atezolizumab; Bev, bevacizumab; C, cyclophosphamide; Cb, carboplatin; Durva, durvalumab; Pt, platinum; pCR, pathologic complete response; Pembro, pembrolizumab; T, Taxane; In network meta-analysis, regimens including FEC-T, P-FAC, ACT, AC-nabP, and ACP (E, epirubicin; F, 5-fluorouracil; nabP, albumin paclitaxel) were merged as anthracycline-taxane based (AT) regimens, and regimens including EC-TCb, PA + Cis/Cb, PCb-FEC, and PCb-AC (Cis, cisplatin) were merged as anthracycline-taxane based + platinum (ATPt) regimens.

**Figure 3:**
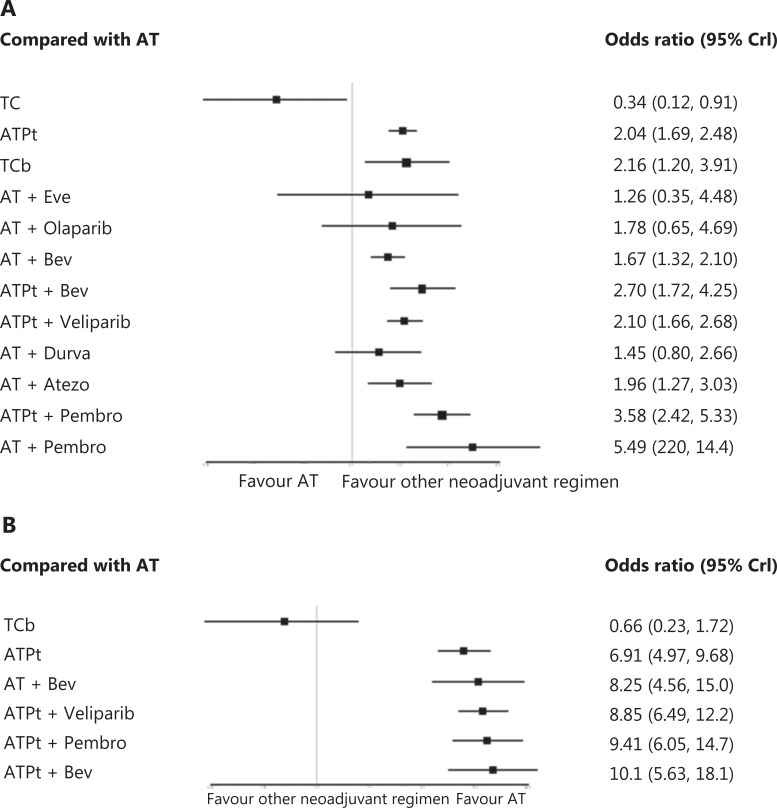
Network meta-analysis (A, pCR; B, aggregated AEs). A. NMA results for all regimens compared with the AT regimen. B. NMA results for all regimens compared with the AT regimen. Notes: A, doxorubicin; Atezo, atezolizumab; Bev, bevacizumab; C, cyclophosphamide; Cb, carboplatin; Durva, durvalumab; Pt, platinum; pCR, pathologic complete response; Pembro, pembrolizumab; T, Taxane. In network meta-analysis, regimens including FECT, P-FAC, ACT, AC-nabP, and ACP (E, epirubicin; F, 5-fluorouracil; nabP, albumin paclitaxel) were merged as anthracycline-taxane based (AT) regimens, and regimens including EC-TCb, PA + Cis/Cb, PCb-FEC, and PCb-AC (Cis, cisplatin) were merged as anthracycline-taxane based + platinum (ATPt) regimens.

A total of 22 RCTs and 5,203 patients were included in the NMA, and a network plot is shown in **Figure 2A** (more details in Online Appendix 5: **Supplementary Tables S5–8**). An improved pCR was detected for the taxane-platinum-anthracycline (ATPt; OR = 2.04, 95% CrI: 1.69, 2.48) and docetaxel-carboplatin (TCb) (OR = 2.16, 95% CrI: 1.20, 3.91) chemotherapy regimens compared with the anthracycline-taxane-based (AT) regimen. The addition of Bev also improved pCR outcomes in patients receiving AT + Bev (OR = 1.67, 95% CrI: 1.32, 2.10) and the ATPt + Bev (OR = 2.70, 95% CrI: 1.72, 4.25). Combination with PARP inhibitors improved pCRs only for the ATPt + veliparib (OR = 2.10, 95% CrI: 1.66, 2.68) regimens. Adding PD-1/PD-L1 inhibitors improved pCRs in the AT + atezolizumab (OR = 1.96, 95% CrI: 1.27, 3.03), the AT + pembrolizumab (OR = 5.49, 95% CrI: 2.20, 14.4), and the ATPt + pembrolizumab (OR = 3.58, 95% CrI: 2.42, 5.33) regimens. (**Figure 3A**; Online Appendix 5: **Supplementary Table S9**).

### Safety

The incidence of aggregated AEs reported in RCTs is summarized in **Table 2**. A total of 5 RCTs with 2,965 patients were connected in the NMA, and a network plot is shown in **Figure 2B** (more details are shown in Online Appendix 6: **Supplementary Tables S11–14**). The incidence of aggregated AEs was lower with TCb than with AT (OR = 0.66, 95% CrI: 0.23, 1.72), but the difference was not statistically significant. In contrast, a significantly higher incidence of aggregated AE was observed with ATPt (OR = 6.91, 95% CrI: 4.97, 9.68), AT + Bev (OR = 8.25, 95% CrI: 4.56, 15.0), ATPt + Bev (OR = 10.1, 95% CrI: 5.63, 18.1), ATPt + veliparib (OR = 8.85, 95% CrI: 6.49, 12.2), and ATPt + pembrolizumab (OR = 9.41, 95% CrI: 6.05, 14.7) than with AT (**Figure 3B**; Online Appendix 6: **Supplementary Table S15**).

**Table 2:** Incidence of aggregated AEs

Study ID	Neoadjuvant regimen	Neoadjuvant regimen in network meta-analysis	No. of participants with aggregated AEs	Sample size	Incidence
Alba 2012	EC-T	NA	25	46	54.35%
Alba 2012	EC-TCb	NA	26	47	55.32%
Geyer 2017	PCb-AC + Veli	ATPt + Veli	272	316	86.08%
Geyer 2017	PCb-AC	ATPt	136	160	85.00%
Geyer 2017	P-AC	AT	71	158	44.94%
Gluz 2018	q3w nabPG	NA	31	178	17.42%
Gluz 2018	q3w nabPCb	NA	16	146	10.96%
Harbeck 2020	nabP-AC + Atezo	NA	103	165	62.42%
Harbeck 2020	nabP-AC	NA	101	168	60.12%
Jovanovic 2017	PCis + Eve	NA	22	96	22.92%
Jovanovic 2017	PCis	NA	6	49	12.24%
Llombart-Cussac 2015	P	NA	5	46	10.87%
Llombart-Cussac 2015	P + weekly Ini	NA	5	46	22.34%
Llombart-Cussac 2015	P + q2w Ini	NA	16	48	33.33%
Loibl 2018	PCb-AC + Veli	ATPt + Veli	222	313	70.93%
Loibl 2018	PCb-AC	ATPt	108	158	68.35%
Loibl 2018	P-AC	AT	23	157	14.65%
Loibl 2019	nabP-AC + Durva	NA	30	92	32.61%
Loibl 2019	nabP-AC	NA	29	82	35.37%
Schmid 2020	PCb-AC/EC + Pembro	ATPt + Pembro	633	781	81.05%
Schmid 2020	PCb-AC/EC	ATPt	295	389	75.84%
Sharma 2019	PCb-AC	ATPt	35	48	72.92%
Sharma 2019	TCb	TCb	11	52	21.15%
Sikov 2015	P-AC	AT	15	107	14.02%
Sikov 2015	P-AC + Bev	AT + Bev	39	105	37.14%
Sikov 2015	PCb-AC	ATPt	29	111	26.13%
Sikov 2015	PCb-AC + Bev	ATPt + Bev	46	110	41.82%

A, doxorubicin; Atezo, atezolizumab; Bev, bevacizumab; C, cyclophosphamide; Cb, carboplatin; Cis, cisplatin; Durva, durvalumab; E, epirubicin; Eve, everolimus; G, gemcitabine; Ini, iniparib; nabP, albumin paclitaxel (weekly cycle if not specifically noted); No.: number; P, paclitaxel (weekly cycle if not specifically noted); Pt, platinum; Pembro, pembrolizumab; q2/3w: every 2/3 weeks; T, docetaxel; Veli, veliparib. In network meta-analysis, regimens including FECT, P-FAC, ACT, AC-nabP, and ACP were merged as anthracycline-taxane based (AT) regimens, and regimens including EC-TCb, PA + Cis/Cb, PCb-FEC, and PCb-AC were merged as anthracycline-taxane based + platinum (ATPt) regimens.

The trace plot and density plot showed a good degree of convergence (Online Appendix 5: **Supplementary Figure S4** and Online Appendix 6: **Supplementary Figure S6**). Except for interventions in which the loop could not be constructed, we observed no significant inconsistencies between the direct and indirect results [inconsistency test results in Online Appendix 5 (**Supplementary Figure S3**)]. Online Appendix 5 and Online Appendix 6 show the mean SUCRA values for providing the hierarchy ranking of the different neoadjuvant regimens in terms of pCR (**Supplementary Table S10** and **Supplementary Figure S2**) and aggregated AEs (**Supplementary Table S16** and **Supplementary Figure S5**). The ranking might be highly biased, and interpretation should be made with caution. Funnel plots were not constructed because the number of included studies in one comparison was less than 10.

## Discussion

TNBC presents a more proliferative pattern with a poorer prognosis than that of the HR-positive pattern, and the biological characteristics of TNBC remain unclear. Some studies have examined the biological characteristics of TNBC and their links to different treatment responses^19^. However, to date, chemotherapy-based treatment remains the first choice to decrease the risk of relapse, and insufficient evidence is available to recommend the routine addition of target drugs, such as immune checkpoint inhibitors, to neoadjuvant chemotherapy in patients with early-stage TNBC^6^.

Currently, neoadjuvant therapy is a standard treatment strategy that can decrease the relapse rate and prolong survival^20^. According to breast cancer guidelines, all adjuvant treatment regimens may be used^20^. To date, many RCTs evaluating neoadjuvant therapy in TNBC have been reported, and no evidence has indicated that any one regimen is superior to others.

As a surrogate for long-term survival^21^, pCR has been used as the primary endpoint in many neoadjuvant clinical trials. This NMA is the first to compare the efficacy and safety of neoadjuvant RCTs combining chemotherapy with VEGF inhibitors, PARP inhibitors, immunotherapy, and other drugs. In this NMA, most of the included studies used the pCR definition of ypT0/is ypN0, which is the most commonly used definition according to the Miller and Payne criteria. Several studies using ypT0 ypN0 were included; this limitation was a result of changes in the pCR definition over the years. In several articles reviewed herein^22–27^, to include as many studies in the NMA as possible, we considered 2 pCR definitions to be coincident, according to clinical practice. This NMA provides several findings of interest for physicians, because it compares neoadjuvant regimens that could not have been compared through conventional meta-analyses, owing to a lack of head-to-head evidence. Before analysis, clinical heterogeneity was fully discussed for the various regimens, and sensitivity analysis was conducted to assess the consistency of the conclusions (Online Appendix 7: **Supplementary Figures S7–11** and **Supplementary Tables S17–22** and Online Appendix 8: **Supplementary Figures S12–15** and **Supplementary Tables S23–28**). The network inconsistency was also low in this analysis. In addition, we performed a comprehensive search with no limitations on language, date, document type, or publication status to identify all relevant published or unpublished RCTs. Four reviewers divided into 2 groups performed the screening, data extraction, and assessment independently to minimize possible bias in the review process.

NCCN guidelines^20^ recommend AC (where A indicates doxorubicin, and C indicates cyclophosphamide) followed by biweekly or weekly paclitaxel as the preferred regimens for HER2-negative breast cancer. AC-T (where T indicates docetaxel) (q3w) or TAC are both commonly used regimens for neoadjuvant/adjuvant therapy in clinical practice. The combination of carboplatin with paclitaxel/docetaxel can be used in patients with TNBC in preoperative settings but is not routinely recommended for most patients.

In this NMA, we found that adding platinum to an AT-based regimen resulted in a significantly greater pCR than observed with the AT regimen alone. Removing anthracycline from the taxane-platinum regimen showed a pCR benefit comparable to that of ATPt, but with a relatively better safety profile, possibly because it combines only 2 chemotherapeutic agents. We additionally conducted an analysis without combining similar regimens (**Supplementary Figure S8**). An improvement effect of pCR was detected for chemotherapy regimens including AC-nabP (where nabP indicates albumin paclitaxel; OR = 1.82, 95% CrI: 1.27, 2.65), TCb (OR = 2.38, 95% CrI: 1.03, 5.46), and PCb-AC (OR = 2.60, 95% CrI: 2.02, 3.36), as compared with AC-P (where P indicates paclitaxel) (**Supplementary Figure S8A**). Including a platinum agent in TNBC neoadjuvant therapy appears to be important to improve pCR benefits, and TCb appears to be effective but better tolerated than an ATPt regimen.

The VEGF inhibitor Bev combined with chemotherapy has demonstrated an improvement over chemotherapy alone, with respect to patient outcomes in several cancers, such as NSCLC^28^ and colorectal cancer^29^. In breast cancer, NCCN guidelines recommend Bev in combination with chemotherapy for only selected patients with recurrent or stage IV disease^20^. In this NMA, we report an improvement in pCR when Bev is added to chemotherapy in the neoadjuvant setting; adding platinum to AT plus Bev appears to be associated with even higher pCR rates, but this benefit is accompanied by higher toxicity (Online Appendix 5: **Supplementary Table S9**).

This NMA also suggests that the use of PD-1/PD-L1 inhibitors (including atezolizumab, pembrolizumab, and veliparib) combined with various chemotherapy regimens, compared with AT alone, significantly improves pCR in patients with TNBC. However, no clear difference was identified between AT plus durvalumab and AT alone. In addition, none of the regimens including a PD-1/PD-L1 inhibitor showed superiority to TCb (Online Appendix 5: **Supplementary Table S9**). Head-to-head trials are needed to confirm these data. In all reported studies, the chemotherapy regimens combined with PD-1/PD-L1 inhibitors were paclitaxel- or nab-paclitaxel-based dose dense regimens, but this combination was associated with a high incidence of aggregated AEs (**Table 2**)^25,30,31^. Additional clinical trials are thus needed to define the optimal chemotherapy regimen to be combined with a PD-1/PD-L1 inhibitor. The superiority of TCb *vs.* AT supports future clinical trials combining TCb with immunotherapy. The results of the NeoPACT (NCT03639948) study, an ongoing phase II single-arm clinical trial combining TCb with pembrolizumab in neoadjuvant treatment of TNBC, are awaited. Immunotherapy may also result in different responses according to the PD-L1 expression level, and patients with PD-L1-positive expression have been found to have higher pCR rates^25,31,32^. Therefore, additional research is needed to define which patients would benefit most from immunotherapy.

This NMA has some limitations. First, the methods (particularly random process and allocation, and the blinding of outcome assessment) were not always adequately reported in the included studies; thus, the risk was unclear for several domains of bias risk. Second, owing to a lack of head-to-head evidence and insufficient data in the included studies, we were unable to explore the comparative effects in some subgroups, such as BRCA mutation, dosage, and treatment duration. Third, owing to limited reports on survival data, long-term survival outcomes should be further assessed.

## Conclusions

In conclusion, the key messages of this NMA are as follows. First, adding platinum to TNBC neoadjuvant therapy (ATPt and TCb) significantly increases pCR beyond that with AT alone. TCb and ATPt show comparable pCR rates, but TCb is better tolerated than ATPt. Second, adding Bev, veliparib, and PD-1/PD-L1 inhibitors to AT and ATPt improves pCR rates. We observed no significant differences between regimens, including PD-1/PD-L1, but ATPt plus PD-1/L1 inhibitor led to relatively higher rates of aggregated AEs. The increased efficacy of regimens should be balanced with patients’ quality of life.
