# Supplementary material for: Platinum is essential in neoadjuvant treatment of triple-negative breast cancer: a network meta-analysis
Source: Cancer Biol Med. 2022 Feb 16;19(5):742–54. doi: 10.20892/j.issn.2095-3941.2021.0529 (PMC9196055; doi:10.20892/j.issn.2095-3941.2021.0529)

**ONLINE APPENDIX**

**Index**

[Appendix 1 Search strategies 3](#_Toc75788462)

[MEDLINE 3](#_Toc75788463)

[Embase 4](#_Toc75788464)

[Cochrane library 6](#_Toc75788465)

[Web of Science 8](#_Toc75788466)

[Appendix 2 Characteristics of included RCTs 11](#_Toc75788467)

[Table S1 Characteristics of Study and Population 11](#_Toc75788468)

[Table S2 Inclusion and Exclusion of included RCTs 19](#_Toc75788469)

[Table S3 Characteristics of Interventions 44](#_Toc75788470)

[Table S4 Characteristics of Outcomes 70](#_Toc75788471)

[Appendix 3 Risk of bias in the included studies 78](#_Toc75788472)

[Figure S1 Risk of Bias Summary 78](#_Toc75788473)

[Appendix 4 Citations for studies in Table 1 79](#_Toc75788474)

[Appendix 5 Results of network meta-analysis (NMA) for pCR 83](#_Toc75788475)

[Table S5 Number of Studies per treatment 83](#_Toc75788476)

[Table S6 Number of n-arm studies 83](#_Toc75788477)

[Table S7 Number of Studies per treatment comparison 83](#_Toc75788478)

[Table S8 Overall heterogeneity and consistency 83](#_Toc75788479)

[Table S9 NMA results 85](#_Toc75788480)

[Table S10 Ranking with SURCA 86](#_Toc75788481)

[Figure S2 Ranking plot 87](#_Toc75788482)

[Figure S3 Node-split plot 87](#_Toc75788483)

[Figure S4 Trace plot and density plot 89](#_Toc75788484)

[Appendix 6 Results of NMA for aggregated AE 90](#_Toc75788485)

[Table S11 Number of Studies per treatment 90](#_Toc75788486)

[Table S12 Number of n-arm studies 90](#_Toc75788487)

[Table S13 Number of Studies per treatment comparison 90](#_Toc75788488)

[Table S14 Overall heterogeneity and consistency 90](#_Toc75788489)

[Table S15 NMA results 90](#_Toc75788490)

[Table S16 Ranking with SURCA 91](#_Toc75788491)

[Figure S5 Ranking plot 91](#_Toc75788492)

[Figure S6 Trace plot and density plot 92](#_Toc75788493)

[Appendix 7 Results of NMA for pCR (detailed regimens) 93](#_Toc75788494)

[Figure S7 Network plot 93](#_Toc75788495)

[Table S17 Number of Studies per treatment 93](#_Toc75788496)

[Table S18 Number of n-arm studies 94](#_Toc75788497)

[Table S19 Number of Studies per treatment comparison 94](#_Toc75788498)

[Table S20 Overall heterogeneity and consistency 94](#_Toc75788499)

[Table S21 NMA results 96](#_Toc75788500)

[Figure S8 Forest plot for all treatments compared with key regimens (A. AC-P; B. PCb-AC+Pembro; C. TCb) 97](#_Toc75788501)

[Table S22 Ranking with SURCA 99](#_Toc75788502)

[Figure S9 Ranking plot 100](#_Toc75788503)

[Figure S10 Node-split plot 100](#_Toc75788504)

[Figure S11 Trace plot and density plot 101](#_Toc75788505)

[Appendix 8 Results of NMA for aggregated AE (detailed regimens) 102](#_Toc75788506)

[Figure S12 Network plot 102](#_Toc75788507)

[Table S23 Number of Studies per treatment 102](#_Toc75788508)

[Table S24 Number of n-arm studies 102](#_Toc75788509)

[Table S25 Number of Studies per treatment comparison 102](#_Toc75788510)

[Table S26 Overall heterogeneity and consistency 103](#_Toc75788511)

[Table S27 NMA results 103](#_Toc75788512)

[Figure S13 Forest plot for all treatments compared with key regimens (AC-P) 103](#_Toc75788513)

[Table S28 Ranking with SURCA 104](#_Toc75788514)

[Figure S14 Ranking plot 104](#_Toc75788515)

[Figure S15 Trace plot and density plot 105](#_Toc75788516)

# Appendix 1 Search strategies

## MEDLINE

#1. MH:exp=(Breast Neoplasms) OR TS=((Breast OR Mamma*) NEAR/3 (Neoplasm* OR Tumor* OR Cancer* OR Carcinoma* OR sarcoma*))

#2. TS=(triple*)

#3. MH=(Neoadjuvant Therapy) OR TS=(Neoadjuvant OR "neo-adjuvant" OR "pre-operative" OR preoperative)

#4. #1 and #2 and #3 1613

#5. MH=(Docetaxel) OR TS=(Docetaxel OR benzoate OR daxotel OR dexotel OR docefrez OR Docetaxol OR "lit 976" OR lit976 OR "n debenzoyl n tert butoxycarbonyl 10 deacetyltaxol" OR "n tert butoxycarbonyl 10 deacetyl n debenzoyltaxol" OR "nsc 628503" OR nsc628503 OR oncodocel OR "rp 56976" OR rp56976 OR taxespira OR taxoter OR taxotere OR texot OR "Taxoltere Metro" OR DOC)

#6. MH=(Paclitaxel) OR TS=(benzoate OR "abi 007" OR abi007 OR abraxane OR paclitaxel OR anzatax OR apealea OR asotax OR biotax OR "bms 181339" OR bms181339 OR "bmy 45622" OR bmy45622 OR bristaxol OR britaxol OR coroxane OR "dts 301" OR dts301 OR "endotag-1" OR formoxol OR genexol OR hunxol OR ifaxol OR infinnium OR intaxel OR "mbt 0206" OR mbt0206 OR medixel OR mitotax OR "nsc 125973" OR nsc 673089 OR nsc125973 OR nsc673089 OR "oas pac 100" OR oaspac100 OR oncogel OR onxol OR pacitaxel OR pacxel OR padexol OR parexel OR paxceed OR paxene OR paxus OR pazenir OR praxel OR "sb 05 terpenoid" OR "sb05 terpenoid" OR taxocris OR taxol OR "taxus drug" OR taycovit OR yewtaxan)

#7. MH=(Cisplatin OR Platinum Compounds) OR TS=(Platinum OR Cisplatin OR "cis Platinum" OR "Platinum Diamminodichloride" OR "cis-Diamminedichloroplatinum" OR Dichlorodiammineplatinum OR "NSC-119875" OR Platino OR Platinol OR Biocisplatinum OR Platidiam OR "cis diammine dichloroplatinum" OR "cis diamminedichloroplatinum" OR platiran OR platistil OR platistin OR platosin OR pronto platamine OR randa OR romcis OR sicatem OR spi 077 OR tecnoplatin OR DDP)

#8. MH=(Carboplatin) OR TS=(Carboplatin OR CBDCA OR Paraplatin* OR Platinwas OR Ribocarbo OR "cis-Diammine(cyclobutanedicarboxylato)platinum II" OR Carboplat OR Neocarbo OR Carbosin OR Carbotec OR Ercar OR "JM-8" OR JM8 OR Nealorin OR "NSC-241240" OR NSC241240 OR Blastocarb OR CBP)

#9. TS=(nedaplatin OR "CGDP-II" OR "cis-diammine(glycolato)platinum II" OR "(glycolato-O,O')diammineplatinum II" OR "NSC 375101D" OR "254-S" OR aqupla OR 254s OR nsc375101d OR SHP OR "Sulfatodaiamino Cyclohxane Platinum" OR "Sulfatodiaminocyclohexane Platinu")

#10. MH=(Oxaliplatin) OR TS=(Oxaliplatin OR Oxalate NEAR/4 platinum OR L-OHP OR Oxaliplatine OR Eloxatine OR Eloxatin OR "ACT 078" OR ACT078 OR axiplatin OR bendaplatin OR crisapla OR croloxat OR dacotin OR dacplat OR ebeoxal OR elatofen OR eloxatin OR eloxatine OR elplat OR euroxaliplatin OR geneplatin OR gessedil OR heloxatin OR lipoxal OR mbp426 OR "mbp 426" OR medoxa OR oksaliplatin OR oksaliplatina OR oplat OR oxali* OR transplastin OR velminox OR xaliplat OR xoplan)

#11. TS=(Lobaplatin OR "D 19466" OR D19466 OR "platinum 1, 2 bis (aminomethyl) cyclobutane lactate")

#12. TS=("nab-paclitaxel" OR "130-nm albumin-bound paclitaxel")

#13. MH:exp=(Fluorouracil) OR TS=(Fluorouracil OR "5 FU" OR "5 HU Hexal" OR "Fluoro-Uracile" OR Efudex OR Fluoroplex OR Flurodex OR Fluracedyl OR "Haemato-FU" OR Neofluor OR Onkofluor OR Ribofluor OR efurix OR fivoflu OR fluorouracile OR flurablastin OR fluroblastine)

#14. MH:exp=(Tegafur) OR TS=(Tegafur OR "FT-207" OR FT207 OR Utefos OR Futraful OR "Sunfural S" OR Uftoral OR Florafur OR Fluorofur OR Ftorafur)

#15. MH:exp=(Capecitabine) OR TS=(Capecitabine OR Xeloda OR gemcitabin* OR gemcite OR gemtro OR gemzar)

#16. MH:exp=(Doxorubicin OR Epirubicin) OR TS=(Farmiblastina OR Ribodoxo OR Rubex OR Adriamycin OR Adriblastin* OR Adriablastin* OR Adrimedac OR "DOXO-cell" OR Doxolem OR Doxorubicin* OR Doxotec OR Myocet OR Onkodox OR adrim OR caelix OR doxorubin OR evacet OR "mcc 465" OR rubidox OR Epirubicin OR Epidoxorubicin OR "Epi Doxorubicin" OR Epiadriamycin OR "Epi-DXR" OR "EPI cell" OR Epilem OR Farmorubicin* OR "IMI 28" OR epirubicin* OR pharmorubicin*)

#17. MH:exp=(cyclophosphamide) OR TS=(cyclophosphamide OR Sendoxan OR "B 518" OR Cytophosphan* OR Cytoxan OR Endoxan OR Neosar OR "NSC-26271" OR Procytox OR Cyclophosphan* OR ciclofosfamida OR cyclophosphamid* OR ledoxan OR ledoxina OR syklofosfamid)

#18. MH:exp=(Programmed Cell Death 1 Receptor OR Programmed Cell Death 1 Ligand 2 Protein OR B7-H1 Antigen OR Nivolumab) OR TS=("programmed cell death protein-1" OR "programmed cell death-ligand 1" OR "PD-1" OR "PD-L1" OR "PD1" OR "PDL1" OR Nivolumab OR Opdivo OR "ONO-4538" OR ONO4538 OR "MDX-1106" OR MDX1106 OR "BMS-936558" OR BMS936558 OR atezolizumab OR "anti-PDL1" OR MPDL3280A OR "MPDL-3280A" OR Tecentriq OR "RG-7446" OR RG7446 OR camrelizumab OR "SHR-1210" OR SHR1210 OR sintilimab OR "IBI 308" OR IBI308 OR pembrolizumab OR "SCH-900475" OR Keytruda OR "MK-3475" OR lambrolizumab OR mk3475 OR sch900475 OR tislelizumab OR "bgb a317" OR bgba317 OR toripalimab OR "js 001" OR js001 OR "tab 001" OR tab001 OR cemiplimab OR REGN2810 OR "REGN 2810" OR "cemiplimab rwlc" OR libtayo OR "sar 439684" OR sar439684 OR avelumab OR "MSB-0010682" OR MSB0010682 OR bavencio OR MSB0010718C OR "MSB-0010718C" OR MSB10718C OR "MSB-10718C" OR "MSB-10682" OR MSB10682 OR "pf 6834635" OR pf6834635 OR "pf 06834635" OR pf06834635 OR durvalumab OR "MEDI-4736" OR MEDI4736 OR Imfinzi OR Envafolimab OR "asc 22" OR asc22 OR "kn 035" OR kn035 OR Sugemalimab OR "cs 1001" OR cs1001 OR "wbp 315" OR wbp315 OR "wbp 3155" OR wbp3155 OR GB226 OR "GLS-010")

#19. MH:exp=(Poly(ADP-ribose) Polymerase Inhibitors) OR TS=(PARP NEAR/1 Inhibitor* OR "Poly ADP-ribosylation" NEAR/1 Inhibitor* OR olaparib OR lynparza OR AZD2281 OR "azd 2281" OR "ku 0059436" OR "ku 59436" OR KU0059436 OR KU59436 OR niraparib OR zejula OR "mk 4827" OR mk4827 OR veliparib OR "abt 888" OR abt888)

#20. #5 OR #6 OR #7 OR #8 OR #9 OR #10 OR #11 OR #12 OR #13 OR #14 OR #15 OR #16 OR #17 OR #18 OR #19

#21. #4 AND #20 587

#22. MH:exp=(Controlled Clinical Trials as Topic OR Randomized Controlled Trials as Topic OR Double-Blind Method OR single-blind method OR Control Groups OR Random Allocation OR cross-over studies) OR TS=(random* OR placebo OR trial OR groups OR crossover OR "cross-over") OR DOCUMENT TYPES:(Controlled Clinical Trial OR Randomized Controlled Trial)

#23. MH:exp=(Animals) NOT MH:exp=(Humans AND Animals)

#24. #21 AND (#22 NOT #23) 329

## Embase

#1. 'breast cancer'/exp OR ((Breast OR Mamma*) NEAR/3 (Neoplasm* OR Tumor* OR Cancer* OR Carcinoma* OR sarcoma*)):ab,ti,kw

#2. triple*:ab,ti,kw

#3. 'neoadjuvant chemotherapy'/exp OR (Neoadjuvant OR "neo-adjuvant" OR "pre-operative" OR preoperative):ab,ti,kw

#4. #1 AND #2 AND #3 4396

#5. 'docetaxel'/exp OR (Docetaxel OR benzoate OR daxotel OR dexotel OR docefrez OR Docetaxol OR "lit 976" OR lit976 OR "n debenzoyl n tert butoxycarbonyl 10 deacetyltaxol" OR "n tert butoxycarbonyl 10 deacetyl n debenzoyltaxol" OR "nsc 628503" OR nsc628503 OR oncodocel OR "rp 56976" OR rp56976 OR taxespira OR taxoter OR taxotere OR texot OR "Taxoltere Metro" OR DOC):ab,ti,kw

#6. 'paclitaxel'/exp OR (benzoate OR "abi 007" OR abi007 OR abraxane OR paclitaxel OR anzatax OR apealea OR asotax OR biotax OR "bms 181339" OR bms181339 OR "bmy 45622" OR bmy45622 OR bristaxol OR britaxol OR coroxane OR "dts 301" OR dts301 OR "endotag-1" OR formoxol OR genexol OR hunxol OR ifaxol OR infinnium OR intaxel OR "mbt 0206" OR mbt0206 OR medixel OR mitotax OR "nsc 125973" OR nsc 673089 OR nsc125973 OR nsc673089 OR "oas pac 100" OR oaspac100 OR oncogel OR onxol OR pacitaxel OR pacxel OR padexol OR parexel OR paxceed OR paxene OR paxus OR pazenir OR praxel OR "sb 05 terpenoid" OR "sb05 terpenoid" OR taxocris OR taxol OR "taxus drug" OR taycovit OR yewtaxan):ab,ti,kw

#7. 'cisplatin'/exp OR 'platinum derivative'/exp OR (Cisplatin OR platinum OR "cis Platinum" OR "Platinum Diamminodichloride" OR "cis-Diamminedichloroplatinum" OR Dichlorodiammineplatinum OR "NSC-119875" OR Platino OR Platinol OR Biocisplatinum OR Platidiam OR "cis diammine dichloroplatinum" OR "cis diamminedichloroplatinum" OR platiran OR platistil OR platistin OR platosin OR pronto platamine OR randa OR romcis OR sicatem OR spi 077 OR tecnoplatin OR DDP):ab,ti,kw

#8. 'carboplatin'/exp OR (Carboplatin OR CBDCA OR Paraplatin* OR Platinwas OR Ribocarbo OR "cis-Diammine(cyclobutanedicarboxylato)platinum II" OR Carboplat OR Neocarbo OR Carbosin OR Carbotec OR Ercar OR "JM-8" OR JM8 OR Nealorin OR "NSC-241240" OR NSC241240 OR Blastocarb OR CBP):ab,ti,kw

#9. 'nedaplatin'/exp OR (nedaplatin OR "CGDP-II" OR "cis-diammine(glycolato)platinum II" OR "(glycolato-O,O)diammineplatinum II" OR "NSC 375101D" OR "254-S" OR aqupla OR 254s OR nsc375101d OR SHP OR "Sulfatodaiamino Cyclohxane Platinum" OR "Sulfatodiaminocyclohexane Platinu"):ab,ti,kw

#10. 'oxaliplatin'/exp OR (Oxaliplatin OR Oxalate NEAR/4 platinum OR L-OHP OR Oxaliplatine OR Eloxatine OR Eloxatin OR "ACT 078" OR ACT078 OR axiplatin OR bendaplatin OR crisapla OR croloxat OR dacotin OR dacplat OR ebeoxal OR elatofen OR eloxatin OR eloxatine OR elplat OR euroxaliplatin OR geneplatin OR gessedil OR heloxatin OR lipoxal OR mbp426 OR "mbp 426" OR medoxa OR oksaliplatin OR oksaliplatina OR oplat OR oxali* OR transplastin OR velminox OR xaliplat OR xoplan):ab,ti,kw

#11. 'lobaplatin'/exp OR (Lobaplatin OR "D 19466" OR D19466 OR "platinum 1, 2 bis (aminomethyl) cyclobutane lactate"):ab,ti,kw

#12. ("nab-paclitaxel" OR "130-nm albumin-bound paclitaxel"):ab,ti,kw

#13. 'fluorouracil'/exp OR (Fluorouracil OR "5 FU" OR "5 HU Hexal" OR "Fluoro-Uracile" OR Efudex OR Fluoroplex OR Flurodex OR Fluracedyl OR "Haemato-FU" OR Neofluor OR Onkofluor OR Ribofluor OR efurix OR fivoflu OR fluorouracile OR flurablastin OR fluroblastine):ab,ti,kw

#14. 'Tegafur'/exp OR (Tegafur OR FT-207 OR FT207 OR Utefos OR Futraful OR Sunfural S OR Uftoral OR Florafur OR Fluorofur OR Ftorafur):ab,ti,kw

#15. 'Capecitabine'/exp OR 'gemcitabine'/exp OR (Capecitabine OR Xeloda OR gemcitabin* OR gemcite OR gemtro OR gemzar):ab,ti,kw

#16. 'Doxorubicin'/exp OR 'Epirubicin'/exp OR (Farmiblastina OR Ribodoxo OR Rubex OR Adriamycin OR Adriblastin* OR Adriablastin* OR Adrimedac OR "DOXO-cell" OR Doxolem OR Doxorubicin* OR Doxotec OR Myocet OR Onkodox OR adrim OR caelix OR doxorubin OR evacet OR "mcc 465" OR rubidox OR Epirubicin OR Epidoxorubicin OR "Epi Doxorubicin" OR Epiadriamycin OR "Epi-DXR" OR "EPI cell" OR Epilem OR Farmorubicin* OR "IMI 28" OR epirubicin* OR pharmorubicin*):ab,ti,kw

#17. 'cyclophosphamide'/exp OR (cyclophosphamide OR Sendoxan OR "B 518" OR Cytophosphan* OR Cytoxan OR Endoxan OR Neosar OR NSC-26271 OR Procytox OR Cyclophosphan* OR ciclofosfamida OR cyclophosphamid* OR ledoxan OR ledoxina OR syklofosfamid):ab,ti,kw

#18. 'programmed death 1 receptor'/exp OR 'programmed death 1 ligand 2'/exp OR 'programmed death 1 ligand 1'/exp OR 'Nivolumab'/exp OR 'atezolizumab'/exp OR 'camrelizumab'/exp OR 'sintilimab'/exp OR 'pembrolizumab'/exp OR 'tislelizumab'/exp OR 'toripalimab'/exp OR 'cemiplimab'/exp OR 'avelumab'/exp OR 'durvalumab'/exp OR 'Envafolimab'/exp OR 'Sugemalimab'/exp OR ("programmed cell death protein-1" OR "programmed cell death-ligand 1" OR "PD-1" OR "PD-L1" OR "PD1" OR "PDL1" OR Nivolumab OR Opdivo OR "ONO-4538" OR ONO4538 OR "MDX-1106" OR MDX1106 OR "BMS-936558" OR BMS936558 OR atezolizumab OR "anti-PDL1" OR MPDL3280A OR "MPDL-3280A" OR Tecentriq OR "RG-7446" OR RG7446 OR camrelizumab OR "SHR-1210" OR SHR1210 OR sintilimab OR "IBI 308" OR IBI308 OR pembrolizumab OR "SCH-900475" OR Keytruda OR "MK-3475" OR lambrolizumab OR mk3475 OR sch900475 OR tislelizumab OR "bgb a317" OR bgba317 OR toripalimab OR "js 001" OR js001 OR "tab 001" OR tab001 OR cemiplimab OR REGN2810 OR "REGN 2810" OR "cemiplimab rwlc" OR libtayo OR "sar 439684" OR sar439684 OR avelumab OR "MSB-0010682" OR MSB0010682 OR bavencio OR MSB0010718C OR "MSB-0010718C" OR MSB10718C OR "MSB-10718C" OR "MSB-10682" OR MSB10682 OR "pf 6834635" OR pf6834635 OR "pf 06834635" OR pf06834635 OR durvalumab OR "MEDI-4736" OR MEDI4736 OR Imfinzi OR Envafolimab OR "asc 22" OR asc22 OR "kn 035" OR kn035 OR Sugemalimab OR "cs 1001" OR cs1001 OR "wbp 315" OR wbp315 OR "wbp 3155" OR wbp3155 OR GB226 OR "GLS-010"):ab,ti,kw

#19. 'nicotinamide adenine dinucleotide adenosine diphosphate ribosyltransferaseinhibitor'/exp OR 'olaparib'/exp OR 'niraparib'/exp OR 'veliparib'/exp OR (PARP NEAR/1 Inhibitor* OR "Poly ADP-ribosylation" NEAR/1 Inhibitor* OR olaparib OR lynparza OR AZD2281 OR "azd 2281" OR "ku 0059436" OR "ku 59436" OR KU0059436 OR KU59436 OR niraparib OR zejula OR "mk 4827" OR mk4827 OR veliparib OR "abt 888" OR abt888)

#20. #5 OR #6 OR #7 OR #8 OR #9 OR #10 OR #11 OR #12 OR #13 OR #14 OR #15 OR #16 OR #17 OR #18 OR #19

#21. #4 AND #20 2080

#22. (('controlled clinical trial'/exp OR 'Controlled Clinical Trial (Topic)'/exp OR 'double blind procedure'/de OR 'control group'/de OR 'crossover procedure'/de OR 'single blind procedure'/de OR 'triple blind procedure'/de OR 'placebo'/de OR 'randomization'/exp) OR (random* OR trial OR groups OR placebo* OR crossover OR "cross-over"):ab,ti,kw) AND 'human'/exp

#23. #21 AND #22 950

## Cochrane library

#1.MeSH descriptor: [Breast Neoplasms] explode all trees 12949

#2.((Breast OR Mamma*) NEAR/3 (Neoplasm* OR Tumor* OR Cancer* OR Carcinoma* OR sarcoma*)):ti,ab,kw 36606

#3.#1 OR #2 36607

#4.(triple*):ti,ab,kw 11881

#5.MeSH descriptor: [Neoadjuvant Therapy] explode all trees 1141

#6.(Neoadjuvant OR "neo-adjuvant" OR "pre-operative" OR preoperative):ti,ab,kw 42220

#7.#5 OR #6 42220

#8.#3 and #4 and #7 601

#9.MeSH descriptor: [Docetaxel] explode all trees 2075

#10.(Docetaxel OR benzoate OR daxotel OR dexotel OR docefrez OR Docetaxol OR "lit 976" OR lit976 OR "n debenzoyl n tert butoxycarbonyl 10 deacetyltaxol" OR "n tert butoxycarbonyl 10 deacetyl n debenzoyltaxol" OR "nsc 628503" OR nsc628503 OR oncodocel OR "rp 56976" OR rp56976 OR taxespira OR taxoter OR taxotere OR texot OR "Taxoltere Metro" OR DOC):ti,ab,kw 8174

#11.MeSH descriptor: [Paclitaxel] explode all trees 3435

#12.(benzoate OR "abi 007" OR abi007 OR abraxane OR paclitaxel OR anzatax OR apealea OR asotax OR biotax OR "bms 181339" OR bms181339 OR "bmy 45622" OR bmy45622 OR bristaxol OR britaxol OR coroxane OR "dts 301" OR dts301 OR "endotag-1" OR formoxol OR genexol OR hunxol OR ifaxol OR infinnium OR intaxel OR "mbt 0206" OR mbt0206 OR medixel OR mitotax OR "nsc 125973" OR nsc 673089 OR nsc125973 OR nsc673089 OR "oas pac 100" OR oaspac100 OR oncogel OR onxol OR pacitaxel OR pacxel OR padexol OR parexel OR paxceed OR paxene OR paxus OR pazenir OR praxel OR "sb 05 terpenoid" OR "sb05 terpenoid" OR taxocris OR taxol OR "taxus drug" OR taycovit OR yewtaxan):ti,ab,kw 10973

#13.MeSH descriptor: [Cisplatin] explode all trees 4977

#14.MeSH descriptor: [Platinum Compounds] explode all trees 5057

#15.(Platinum OR Cisplatin OR "cis Platinum" OR "Platinum Diamminodichloride" OR "cis-Diamminedichloroplatinum" OR Dichlorodiammineplatinum OR "NSC-119875" OR Platino OR Platinol OR Biocisplatinum OR Platidiam OR "cis diammine dichloroplatinum" OR "cis diamminedichloroplatinum" OR platiran OR platistil OR platistin OR platosin OR pronto platamine OR randa OR romcis OR sicatem OR spi 077 OR tecnoplatin OR DDP):ti,ab,kw 18926

#16.MeSH descriptor: [Carboplatin] explode all trees 1549

#17.(Carboplatin OR CBDCA OR Paraplatin* OR Platinwas OR Ribocarbo OR "cis-Diammine(cyclobutanedicarboxylato)platinum II" OR Carboplat OR Neocarbo OR Carbosin OR Carbotec OR Ercar OR "JM-8" OR JM8 OR Nealorin OR "NSC-241240" OR NSC241240 OR Blastocarb OR CBP):ti,ab,kw 7328

#18.(nedaplatin OR "CGDP-II" OR "cis-diammine(glycolato)platinum II" OR "(glycolato-O,O')diammineplatinum II" OR "NSC 375101D" OR "254-S" OR aqupla OR 254s OR nsc375101d OR SHP OR "Sulfatodaiamino Cyclohxane Platinum" OR "Sulfatodiaminocyclohexane Platinu"):ti,ab,kw 360

#19.MeSH descriptor: [Oxaliplatin] explode all trees 684

#20.(Oxaliplatin OR Oxalate NEAR/4 platinum OR L-OHP OR Oxaliplatine OR Eloxatine OR Eloxatin OR "ACT 078" OR ACT078 OR axiplatin OR bendaplatin OR crisapla OR croloxat OR dacotin OR dacplat OR ebeoxal OR elatofen OR eloxatin OR eloxatine OR elplat OR euroxaliplatin OR geneplatin OR gessedil OR heloxatin OR lipoxal OR mbp426 OR "mbp 426" OR medoxa OR oksaliplatin OR oksaliplatina OR oplat OR oxali* OR transplastin OR velminox OR xaliplat OR xoplan):ti,ab,kw 4650

#21.(Lobaplatin OR "D 19466" OR D19466 OR "platinum 1, 2 bis (aminomethyl) cyclobutane lactate" OR "nab-paclitaxel" OR "130-nm albumin-bound paclitaxel" OR Fluorouracil OR "5 FU" OR "5 HU Hexal" OR "Fluoro-Uracile" OR Efudex OR Fluoroplex OR Flurodex OR Fluracedyl OR "Haemato-FU" OR Neofluor OR Onkofluor OR Ribofluor OR efurix OR fivoflu OR fluorouracile OR flurablastin OR fluroblastine):ti,ab,kw 12695

#22.MeSH descriptor: [Fluorouracil] explode all trees 6017

#23.(Tegafur OR "FT-207" OR FT207 OR Utefos OR Futraful OR "Sunfural S" OR Uftoral OR Florafur OR Fluorofur OR Ftorafur OR Capecitabine OR Xeloda OR gemcitabin* OR gemcite OR gemtro OR gemzar):ti,ab,kw 10027

#24.MeSH descriptor: [Tegafur] explode all trees 589

#25.MeSH descriptor: [Capecitabine] explode all trees 1189

#26.MeSH descriptor: [Doxorubicin] explode all trees 4774

#27.MeSH descriptor: [Epirubicin] explode all trees 1191

#28.(Farmiblastina OR Ribodoxo OR Rubex OR Adriamycin OR Adriblastin* OR Adriablastin* OR Adrimedac OR "DOXO-cell" OR Doxolem OR Doxorubicin* OR Doxotec OR Myocet OR Onkodox OR adrim OR caelix OR doxorubin OR evacet OR "mcc 465" OR rubidox OR Epirubicin OR Epidoxorubicin OR "Epi Doxorubicin" OR Epiadriamycin OR "Epi-DXR" OR "EPI cell" OR Epilem OR Farmorubicin* OR "IMI 28" OR epirubicin* OR pharmorubicin*):ti,ab,kw 11166

#29.MeSH descriptor: [Cyclophosphamide] explode all trees 5455

#30.(cyclophosphamide OR Sendoxan OR "B 518" OR Cytophosphan* OR Cytoxan OR Endoxan OR Neosar OR "NSC-26271" OR Procytox OR Cyclophosphan* OR ciclofosfamida OR cyclophosphamid* OR ledoxan OR ledoxina OR syklofosfamid):ti,ab,kw 12099

#31.MeSH descriptor: [Programmed Cell Death 1 Receptor] explode all trees 59

#32.MeSH descriptor: [Programmed Cell Death 1 Ligand 2 Protein] explode all trees 2

#33.MeSH descriptor: [B7-H1 Antigen] explode all trees 57

#34.MeSH descriptor: [Nivolumab] explode all trees 422

#35.("programmed cell death protein-1" OR "programmed cell death-ligand 1" OR "PD-1" OR "PD-L1" OR "PD1" OR "PDL1" OR Nivolumab OR Opdivo OR "ONO-4538" OR ONO4538 OR "MDX-1106" OR MDX1106 OR "BMS-936558" OR BMS936558 OR atezolizumab OR "anti-PDL1" OR MPDL3280A OR "MPDL-3280A" OR Tecentriq OR "RG-7446" OR RG7446 OR camrelizumab OR "SHR-1210" OR SHR1210 OR sintilimab OR "IBI 308" OR IBI308 OR pembrolizumab OR "SCH-900475" OR Keytruda OR "MK-3475" OR lambrolizumab OR mk3475 OR sch900475 OR tislelizumab OR "bgb a317" OR bgba317 OR toripalimab OR "js 001" OR js001 OR "tab 001" OR tab001 OR cemiplimab OR REGN2810 OR "REGN 2810" OR "cemiplimab rwlc" OR libtayo OR "sar 439684" OR sar439684 OR avelumab OR "MSB-0010682" OR MSB0010682 OR bavencio OR MSB0010718C OR "MSB-0010718C" OR MSB10718C OR "MSB-10718C" OR "MSB-10682" OR MSB10682 OR "pf 6834635" OR pf6834635 OR "pf 06834635" OR pf06834635 OR durvalumab OR "MEDI-4736" OR MEDI4736 OR Imfinzi OR Envafolimab OR "asc 22" OR asc22 OR "kn 035" OR kn035 OR Sugemalimab OR "cs 1001" OR cs1001 OR "wbp 315" OR wbp315 OR "wbp 3155" OR wbp3155 OR GB226 OR "GLS-010"):ti,ab,kw 5285

#36.MeSH descriptor: [Poly(ADP-ribose) Polymerase Inhibitors] explode all trees 51

#37.(ARP NEAR/1 Inhibitor* OR "Poly ADP-ribosylation" NEAR/1 Inhibitor* OR olaparib OR lynparza OR AZD2281 OR "azd 2281" OR "ku 0059436" OR "ku 59436" OR KU0059436 OR KU59436 OR niraparib OR zejula OR "mk 4827" OR mk4827 OR veliparib OR "abt 888" OR abt888):ti,ab,kw 734

#38.#9 OR #10 OR #11 OR #12 OR #13 OR #14 OR #15 OR #16 OR #17 OR #18 OR #19 OR #20 OR #21 OR #22 OR #23 OR #24 OR #25 OR #26 OR #27 OR #28 OR #29 OR #30 OR #31 OR #32 OR #33 OR #34 OR #35 OR #36 OR #37 59531

#39.#8 AND #38 485

## Web of Science

#1. TS=((Breast OR Mamma*) NEAR/3 (Neoplasm* OR Tumor* OR Cancer* OR Carcinoma* OR sarcoma*))

#2. TS=(triple*)

#3. TS=(Neoadjuvant OR "neo-adjuvant" OR "pre-operative" OR preoperative)

#4. #1 and #2 and #3 2315

#5. TS=(Docetaxel OR benzoate OR daxotel OR dexotel OR docefrez OR Docetaxol OR "lit 976" OR lit976 OR "n debenzoyl n tert butoxycarbonyl 10 deacetyltaxol" OR "n tert butoxycarbonyl 10 deacetyl n debenzoyltaxol" OR "nsc 628503" OR nsc628503 OR oncodocel OR "rp 56976" OR rp56976 OR taxespira OR taxoter OR taxotere OR texot OR "Taxoltere Metro" OR DOC)

#6. TS=(benzoate OR "abi 007" OR abi007 OR abraxane OR paclitaxel OR anzatax OR apealea OR asotax OR biotax OR "bms 181339" OR bms181339 OR "bmy 45622" OR bmy45622 OR bristaxol OR britaxol OR coroxane OR "dts 301" OR dts301 OR "endotag-1" OR formoxol OR genexol OR hunxol OR ifaxol OR infinnium OR intaxel OR "mbt 0206" OR mbt0206 OR medixel OR mitotax OR "nsc 125973" OR nsc 673089 OR nsc125973 OR nsc673089 OR "oas pac 100" OR oaspac100 OR oncogel OR onxol OR pacitaxel OR pacxel OR padexol OR parexel OR paxceed OR paxene OR paxus OR pazenir OR praxel OR "sb 05 terpenoid" OR "sb05 terpenoid" OR taxocris OR taxol OR "taxus drug" OR taycovit OR yewtaxan)

#7. TS=(Cisplatin OR platinum OR "cis Platinum" OR "Platinum Diamminodichloride" OR "cis-Diamminedichloroplatinum" OR Dichlorodiammineplatinum OR "NSC-119875" OR Platino OR Platinol OR Biocisplatinum OR Platidiam OR "cis diammine dichloroplatinum" OR "cis diamminedichloroplatinum" OR platiran OR platistil OR platistin OR platosin OR pronto platamine OR randa OR romcis OR sicatem OR spi 077 OR tecnoplatin OR DDP)

#8. TS=(Carboplatin OR CBDCA OR Paraplatin* OR Platinwas OR Ribocarbo OR "cis-Diammine(cyclobutanedicarboxylato)platinum II" OR Carboplat OR Neocarbo OR Carbosin OR Carbotec OR Ercar OR "JM-8" OR JM8 OR Nealorin OR "NSC-241240" OR NSC241240 OR Blastocarb OR CBP)

#9. TS=(nedaplatin OR "CGDP-II" OR "cis-diammine(glycolato)platinum II" OR "(glycolato-O,O')diammineplatinum II" OR "NSC 375101D" OR "254-S" OR aqupla OR 254s OR nsc375101d OR SHP OR "Sulfatodaiamino Cyclohxane Platinum" OR "Sulfatodiaminocyclohexane Platinu")

#10. TS=(Oxaliplatin OR Oxalate NEAR/4 platinum OR L-OHP OR Oxaliplatine OR Eloxatine OR Eloxatin OR "ACT 078" OR ACT078 OR axiplatin OR bendaplatin OR crisapla OR croloxat OR dacotin OR dacplat OR ebeoxal OR elatofen OR eloxatin OR eloxatine OR elplat OR euroxaliplatin OR geneplatin OR gessedil OR heloxatin OR lipoxal OR mbp426 OR "mbp 426" OR medoxa OR oksaliplatin OR oksaliplatina OR oplat OR oxali* OR transplastin OR velminox OR xaliplat OR xoplan)

#11. TS=(Lobaplatin OR "D 19466" OR D19466 OR "platinum 1, 2 bis (aminomethyl) cyclobutane lactate")

#12. TS=("nab-paclitaxel" OR "130-nm albumin-bound paclitaxel")

#13. TS=(Fluorouracil OR "5 FU" OR "5 HU Hexal" OR "Fluoro-Uracile" OR Efudex OR Fluoroplex OR Flurodex OR Fluracedyl OR "Haemato-FU" OR Neofluor OR Onkofluor OR Ribofluor OR efurix OR fivoflu OR fluorouracile OR flurablastin OR fluroblastine)

#14. TS=(Tegafur OR "FT-207" OR FT207 OR Utefos OR Futraful OR "Sunfural S" OR Uftoral OR Florafur OR Fluorofur OR Ftorafur)

#15. TS=(Capecitabine OR Xeloda OR gemcitabin* OR gemcite OR gemtro OR gemzar)

#16. TS=(Farmiblastina OR Ribodoxo OR Rubex OR Adriamycin OR Adriblastin* OR Adriablastin* OR Adrimedac OR "DOXO-cell" OR Doxolem OR Doxorubicin* OR Doxotec OR Myocet OR Onkodox OR adrim OR caelix OR doxorubin OR evacet OR "mcc 465" OR rubidox OR Epirubicin OR Epidoxorubicin OR "Epi Doxorubicin" OR Epiadriamycin OR "Epi-DXR" OR "EPI cell" OR Epilem OR Farmorubicin* OR "IMI 28" OR epirubicin* OR pharmorubicin*)

#17. TS=(cyclophosphamide OR Sendoxan OR "B 518" OR Cytophosphan* OR Cytoxan OR Endoxan OR Neosar OR "NSC-26271" OR Procytox OR Cyclophosphan* OR ciclofosfamida OR cyclophosphamid* OR ledoxan OR ledoxina OR syklofosfamid)

#18. TS=("programmed cell death protein-1" OR "programmed cell death-ligand 1" OR "PD-1" OR "PD-L1" OR "PD1" OR "PDL1" OR Nivolumab OR Opdivo OR "ONO-4538" OR ONO4538 OR "MDX-1106" OR MDX1106 OR "BMS-936558" OR BMS936558 OR atezolizumab OR "anti-PDL1" OR MPDL3280A OR "MPDL-3280A" OR Tecentriq OR "RG-7446" OR RG7446 OR camrelizumab OR "SHR-1210" OR SHR1210 OR sintilimab OR "IBI 308" OR IBI308 OR pembrolizumab OR "SCH-900475" OR Keytruda OR "MK-3475" OR lambrolizumab OR mk3475 OR sch900475 OR tislelizumab OR "bgb a317" OR bgba317 OR toripalimab OR "js 001" OR js001 OR "tab 001" OR tab001 OR cemiplimab OR REGN2810 OR "REGN 2810" OR "cemiplimab rwlc" OR libtayo OR "sar 439684" OR sar439684 OR avelumab OR "MSB-0010682" OR MSB0010682 OR bavencio OR MSB0010718C OR "MSB-0010718C" OR MSB10718C OR "MSB-10718C" OR "MSB-10682" OR MSB10682 OR "pf 6834635" OR pf6834635 OR "pf 06834635" OR pf06834635 OR durvalumab OR "MEDI-4736" OR MEDI4736 OR Imfinzi OR Envafolimab OR "asc 22" OR asc22 OR "kn 035" OR kn035 OR Sugemalimab OR "cs 1001" OR cs1001 OR "wbp 315" OR wbp315 OR "wbp 3155" OR wbp3155 OR GB226 OR "GLS-010")

#19. TS=(PARP NEAR/1 Inhibitor* OR "Poly ADP-ribosylation" NEAR/1 Inhibitor* OR olaparib OR lynparza OR AZD2281 OR "azd 2281" OR "ku 0059436" OR "ku 59436" OR KU0059436 OR KU59436 OR niraparib OR zejula OR "mk 4827" OR mk4827 OR veliparib OR "abt 888" OR abt888)

#20. #5 OR #6 OR #7 OR #8 OR #9 OR #10 OR #11 OR #12 OR #13 OR #14 OR #15 OR #16 OR #17 OR #18 OR #19

#21. #4 AND #20 1014

#22. TS=(random* OR placebo OR trial OR groups OR crossover OR "cross-over")

#23. #21 AND #22 618

# Appendix 2 Characteristics of included RCTs

## Table S1 Characteristics of Study and Population

| **Study ID** | **Trial Number** | **Definition (HER2-)** | **Definition (HR-)** | **Diagnostic criteria** | **Sample size at randomization** | **Sample size for analysis** | **Female** | **ECOG PS** | **BRCA mutated (BRCA-1; BRCA-2)** |
| --- | --- | --- | --- | --- | --- | --- | --- | --- | --- |
| Alba 2012 | GEICAM/2006-03 NCT00432172 | NR | NR | Histologically confirmed (by surgical or core biopsy) basal-like breast cancer, defined as ER negative, PgR negative, HER2 negative, and cytokeratin 5/6 or epidermal growth factor receptor (EGFR) positive by immunohistochemistry (IHC), were included. | 94 | 93 | 94 | 0-1 (eligibility requirement) 0: n=82 1: n=11 unknown: n=1 | NR |
| Ando 2014 | UMIN000003355 | HER2-negative disease was defined as a score of 0 or 1 + by immuno- histochemistry (IHC) or HER2 gene copy: chromosome 17 ratio of ＜2.0 by fluorescence in situ hybridization (FISH). The HER2 status of CNB specimens was determined by IHC and/or FISH performed at each institution before study enrollment, and was not subject to central review. | HR status [estrogen receptor (ER) and progesterone receptor (PgR)] of CNB specimens was assessed by IHC, for which C10 % staining of cancer cell nuclei was diagnosed as positive. | NR | 75 | 75 | 75 | 0-2 (eligibility requirement). | NR |
| Bear 2012 | NCT00408408 | NR | NR | NR | 490 | 490 | 490 | 0-1 (eligibility requirement) | NR |
| Chen 2016 | NATT | NR | NR | Triple negative breast cancer (TNBC) was defined as ER-, PR-, and HER2- | 102 | 96 patients were eligible for safety analysis; 95 patients for efficacy analysis. | 49 | NR | NR |
| Earl 2015 | ARTemis NCT01093235 ISRCTN(68502941) EudraCT(2008-002322-11) | HER2 negative was defined as immunohistochemistry of 0/1+, or if 2+, fluorescence in- situ hybridisation showed no evidence of amplification of the HER2 gene. | We regarded hormone oestrogen receptor (ER) status as negative when Allred score was 0–2/8; ER weakly positive was 3–5/8; and ER strongly positive was 6–8/8. These ER categories were based on those already used by our group in the tAnGo trial and subsequently confirmed by Petit and colleagues to be predictive of neoadjuvant chemotherapy response (pathological complete response) in ER-positive tumours. | Here, we took patients who were categorized as ER negative in this HER2 negative population as our TNBC group. | 248 | 241 | 248 | 0-2 (eligibility requirement). | NR |
| Fasching 2019 | GeparOLA NCT02789332 | NR | NR | NR | 77 | 77 | NR | NR | NR |
| Gerber 2013 | GeparQuinto NCT00567554 | Dako-HercepTest: score 0 or 1+, in score 2 no gene amplification by in situ hybridization | estrogen and progesterone receptor <10% | TNBC was defined as no HER2 overexpression (Dako-HercepTest: score 0 or 1+, in score 2 no gene amplification by in situ hybridization) and hormone receptor (HR) expression (estrogen and progesterone receptor <10%) by immunohistochemistry as assessed by the local pathology. Tumors without any HR expression (estrogen and progesterone receptor 0%) were defined as strictly triple-negative. | 678 | 663 | 663 | NR | BRCA1/2 mutation (18.3%) 1) BRCA1 mutation (15.0%): intervention 1-34 (14.6); intervention 2-40 (15.4) 1) BRCA2 mutation (3.2%): intervention 1-5 (2.1); intervention 2-11 (4.2) |
| Geyer 2017 | NCT02032277 | NR | NR | NR | 634 | 634 | 634 | 0-1 (eligibility requirement) | NR |
| Gianni 2018 | NCT01822314 | NR | NR | ER and progesterone receptor (PgR)<1%; ERBB2/HER2 0/1 positive, or 2 positive and in situ hydridization (ISH) negative. | 219 | 219 | 219 | 0-1 (eligibility requirement) | NR |
| Gigolaeva 2019 | NR | NR | NR | NR | 192 | 192 | NR | NR | 23 patients (12%) have BRCA1 gene mutation. |
| Gluz 2018 | WSG-ADAPT-TN NCT01815242 | HER2-negative, as defined by current guidelines | NR | estrogen receptor/progesterone receptor < 1%, human epidermal growth factor receptor 2–negative, cT1c-cT4c, cN0/+ | 336 | 336 | 336 | < 1 (eligibility requirement) | NR |
| Gonzalez-Angulo 2014 | NCT01426880 | NR | NR | Triple-negative breast cancers (TNBC) lack expression of estrogen receptor, progesterone receptor, and HER2 | 62 | 50 | 50 | NR | NR |
| Harbeck 2020 | IMpassion031 NCT03197935 | NR | NR | NR | 333 | 333 | NR | 0: n=309 1: n=22 Missing: n=2 | NR |
| Ishikawa 2016 | KBOG1101 UMIN000002215 | NR | NR | NR | 66 | 66 | 66 | 0: n=33 1: n=0 | NR |
| Jovanovic 2017 | NCT00930930 | NR | NR | TNBC lacks expression of estrogen, progesterone, and HER2 receptors | 145 | 145 | NR | 0-1 (eligibility requirement) | BRCA1 or BRCA2 Mutation: 1) present: 6 (2 in everolimus group, 4 in placebo group) 2) absent: 20 (17 in everolimus group, 3 in placebo group) 3) unknow: 119 (77 in everolimus group, 42 in placebo group) |
| Kummel 2017 | GENEVIEVE NCT01779479 | NR | NR | Triple negativity was defined as oestrogen (ER)- and progesterone (PgR)-negative, <1% positive stained cells by immunohistochemistry (IHC), HER2 negativity, IHC 0þ, 1þ or IHC 2þ and FISH/SISH/CISH (ratio < 2.0) negative. | 131 | 131 | 131 | 0-1 (eligibility requirement) | NR |
| Llombart-Cussac 2015 | NCT01204125 | HER2-negative defined as IHC: 0 or 1+; or IHC: 2+ and FISH-negative. | ER and PgR negative defined as <10 % tumor staining by immuno- histochemistry (IHC) or Allred <5. | NR | 141 | safety: 140 pCR: 141 (ITT) | 141 | 0: n=134 1: n=7 | NR |
| Loi 2019 | NCT02622074 | HER2-negative by immunohistochemistry (0 or 1þ) or fluorescence in situ hybridization. | estrogen receptor (ER)/progesterone receptor (PR)-negative by immunohistochemistry (<1% of tumor cells positive for ER/PR irrespective of staining intensity). | NR | 60 | 60 | 60 | 0-1 (eligibility requirement) | NR |
| Loibl 2018 | BrighTNess  NCT02032277 | NR | NR | Triple-negative breast cancer was defined following the American Society of Clinical Oncology and College of American Pathologists guidelines18 as less than 1% positivity for oestrogen receptor and progesterone receptor expression by immunohistochemical staining, and either a score of 0–1+ in HER2 staining by immunohistochemistry or no HER2 gene amplification by fluorescent or chromogenic in- situ hybridisation | 634 | 634 | 634 | 0-1 (eligibility requirement) | 1) Deleterious mutation (14.7%)： intervention 1-45 (14%); intervention 2-25 (16%); intervention 3-23 (15%) 2) No deleterious mutation:  intervention 1-271; intervention 2-135; intervention 3-135 |
| Loibl 2019 | GeparNuevo NCT02685059 | HER2 negativity defined as either IHC 0/1 or IHC 2+ and in situ hybridisation of either ratio <2.0 or <6 copies of HER2 per tumour cell. | NR | NR | 174 | 174 | 174 | 0-1 (eligibility requirement) | NR |
| Martinez 2015 | NR | NR | NR | NR | 61 | 61 | NR | NR | NR |
| Mayer 2019 | TBCRC 030 | NR | ER/PR negative (<= 5%) | invasive breast cancer; clinical stage I (T1 > 1.5 cm), or Stage II-III. | 140 | 138 | NR | NR | NR (ppt有对BRCA的解读，但是没有数据） |
| Nahleh 2016 | SWOG S0800 NCI CDR0000636131 | HER-2 status was determined locally according to the 2007 American Society of Clinical Oncology/College of American Pathology guidelines. | ER/PgR+ disease (defined as >=1 % expression by immunohistochemistry (IHC) stain). | NR | 67 | 67 | 67 | NR | NR |
| Nanda 2020 | I-SPY2 NCT01042379 | NR | NR | NR | NR | 88 | 88 | 0-1 (eligibility requirement) | NR |
| Rugo 2016 | I-SPY2 NCT01042379 | NR | NR | HER2-negative, estrogen-receptor–negative, and progesterone-receptor–negative | NR | 60 | 60 | 0-1 (eligibility requirement) | NR |
| Schmid 2020 | KEYNOTE-522 NCT03036488 | NR | NR | triple-negativebreastcancerinallfoci(as defined by the guidelines of the American Society of Clinical Oncology–College of American Patholo- gists) | 1174 | pCR: 602 safety: 1170 | NR | 0: n=1019 1: n=155 | NR |
| Schneeweiss 2019 | GeparOcto-GBG 84 NCT02125344 | HER2-positivity as immunohistochemical staining of 3+ or in case of 2+ by a HER2 to chromosome 17 (HER2:- CEP17) ratio ≥2.0 analysed by dual-probe in-situ hybridisation. | Hormone receptor (HR)-negativity was defined as <1% stained cells. | NR | 403 | 403 | 403 | NR | NR |
| Sharma 2019 | NCT02413320 | HER2 negativity was defined per ASCO/CAP guidelines. | ER and PgR negative defined as immunohistochemical (IHC) nuclear staining of <= 10%. | NR | 100 | 100 | 100 | 0-1 (eligibility requirement) | 17 carried aBRCA1/2 mutation (17%) |
| Sikov 2015 | CALGB 40603 NCT00861705 | HER2 negativity defined by immunohistochemical (IHC) staining 0 to 1+ or fluorescence in situ hybridization ratio＜2.0 if IHC 2+ or IHC not performed. | NR | Eligible patients had operable, biopsy-confirmed, previously untreated, clinicalstage II to III noninflammatory invasive breast cancer, with ER and PgR expression 10% and HER2 negativity, defined by immunohistochemical (IHC) staining 0 to 1+ or fluorescence in situ hybridization ratio＜2.0 if IHC 2+ or IHC not performed. | 454 | 454 | NR | NR | NR |
| Tung 2020 | INFORM NCT01670500 | HER2-negative invasive breast cancer, defined by immunohistochemical (IHC) staining 0 to 1+ or fluorescence in situ hybridization ratio , 2.0 if IHC 2+ or IHC was not performed. | NR | NR | 83 | 83 | NR | 0-1 (eligibility requirement) | NR |
| Untch 2016 | GeparSepto NCT01583426 | NR | NR | cT1c and ER-neg and PR-neg | 276 | 276 | 276 | NR | NR |
| Von Minckwitz 2014 | GeparSixto NCT01426880 | HercepTest [Dako] score 0 or 1+ or gene amplification ratio <2.2 by in-situ hybridisation | oestrogen and progesterone receptor levels of less than 1% | Triple-negative status was defined as oestrogen and progesterone receptor levels of less than 1% and HER2-negative (HercepTest [Dako] score 0 or 1+ or gene amplification ratio <2·2 by in-situ hybridisation) | NR | 315 | 315 | NR | BRCA1 and BRCA2 mutation: intervention 1-26; intervention 2-24 |
| Wu 2018 | ChiCTR-TRC-14005019 | HER2-negative (IHC 0-1 + or FISH ratio < 2.0) | ER- and PR-negative (defined as ER and PR expression < 10% nuclei staining, according to the 2010 American Society of Clinical Oncology/College of American Pathologists guidelines) | NR | 128 | 125 | NR | NR | NR |
| Zhang 2016 | NCT01276769 | Her-2 was considered negative if IHC scoring was 0/1+, or 2+ but FISH negative. | ER and PR were defined as negative when < 10% of nuclei were positively stained in ten high-power fields. | The clinical evaluation of tumors included physical examination, mammography, ultrasonography of the breast and regional lymph nodes, and breast magnetic resonance imaging (MRI). The clinical or pathological stages were confirmed in accordance with the American Joint Committee on Cancer manual (AJCC, the 6th edition). | 91 | safety: 91 (ITT) pCR: 87 | 91 | 0-1 (eligibility requirement) | NR |
| Zhang 2020 | NeoCART | NR | NR | NR | 93 | 86 | NR | 0-1 (eligibility requirement) | Deleterious mutation: total=5 (12.2%), DCb=3, EC-D=2 No deleterious mutation: total=36, DCb=16, EC-D=20 Not detected: total=47, DCb=25, EC-D=22 |

## Table S2 Inclusion and Exclusion of included RCTs

| **Study ID** | **Inclusion Criteria** | **Exclusion Criteria** |
| --- | --- | --- |
| Alba 2012 | Patients >18-years old and with histologically confirmed by surgical or core biopsy) basal-like breast cancer, defined as ER negative, PgR negative, HER2 negative, and cytokeratin 5/6 or epidermal growth factor receptor EGFR) positive by immunohistochemistry IHC), were included. Tumor size had to be >2 cm or less if there was axillary involvement pathologically confirmed). Patients were required to have ECOG performance status <=1, normal cardiac function, and adequate bone marrow reserve and liver and renal functions. | Patients were excluded if they had received previous treatment for the present disease, previous anthracycline and/or taxane administration, have concurrent treatment with corticosteroids, selective estrogen-receptor modulators or hormonal replacement therapy, had inflammatory, bilateral invasive, or metastatic breast cancer, or if they had any other severe or uncontrolled systemic disease. Adequate contraception and a negative pregnancy test were required for women with child-bearing potential. Patients with a previous history of cancer other than skin no-melanoma), or cervix tumors adequately treated and other cancers treated more than 10 years before the study entry, were also excluded. |
| Ando 2014 | 1) Eligible patients had previously untreated, unilateral, histologically confirmed, invasive, non-inflammatory, breast carcinoma. 2) Patients with a tumor [2.0 cm at the largest dimension by ultrasonography, or B2.0 cm with axillary lymph node metastasis clinically diagnosed as positive, were eligible clinical stage II and IIIA) . 3) Other requirements included age 18–70 years, ECOG performance status 0–2, adequate bone marrow function absolute granulocyte count C1,500/mm3 and platelet count C100,000/mm3) , liver function total bilirubin B1.5 mg/dL and liver transaminase [aspartate amino- transferase AST) and alanine aminotransferase ALT) ] B60 IU/L) , and renal function serum creatinine B1.5 mg/dL) , and written informed consent. | Patients with T4, N3, supraclavicular lymph node), or distant metastatic disease M1) were excluded from this study. Patients with a history of ischemic cardiac disease were excluded. |
| Bear 2012 | Women with primary operable HER2-negative breast cancer diagnosed by means of a core needle biopsy were eligible for participation in the study. Patients were required to have a palpable primary tumor at least 2.0 cm in diameter in the breast, as assessed by physical examination, and to be classi- fied as having tumor stage T1c to T3, nodal stage N0 to N2a, and metastasis stage M0. Other key eligibility criteria were an Eastern Cooperative Oncology Group ECOG) performance status of 0 or 1 with 0 indicating that the patient is fully active and able to carry on all predisease activities without restriction and 1 indicating that the patient is restricted in physically strenuous activity but is ambulatory and able to carry out work of a light or sedentary nature, such as light housework or office work) and normal left ventricular ejection fraction, determined by multiple-gated acquisition scanning or echocardiography within 3 months before study entry. | NR |
| Chen 2016 | Patients between the ages of 18 and 70 years with histological confirmed primary invasive breast cancer stages IIB or III according to the American Joint Committee on Cancer staging system version 6) were considered eligible. | Prior chemotherapy, radiotherapy or hormone treatment was forbidden. |
| Earl 2015 | 1) We enrolled women aged 18 years or older with a histological diagnosis of early invasive breast cancer, and a radiological tumour size of more than 20 mm with or without axillary involvement. 2) Patients with inflammatory cancer, T4 tumours with direct extension to the chest wall or skin, and ipsilateral supraclavicular lymph node involvement were eligible with any size of primary tumour. 3) All patients were HER2 negative. 4) Other eligibility criteria were adequate cardiac function left ventricular ejection fraction within the normal institutional range, as assessed by multiple- gated acquisition scan or echocardiogram), adequate bone marrow, hepatic, and renal function, and appropriate Eastern Cooperative Oncology Group ECOG) performance status 0–2). In view of potential side-effects from bevacizumab, patients had to have no previous diagnosis of ischaemic heart disease, cerebrovascular disease, peripheral vascular disease, arterial or venous thromboembolic disease, cardiac failure, gastroduodenal ulcer, symptomatic diverticulitis, or inflammatory bowel disease. Additionally, no uncontrolled hypertension, defined by a systolic pressure greater than 150 mm Hg or diastolic pressure greater than 90 mm Hg, with or without antihypertensive medication was allowed. Patients with initial increases in blood pressure were eligible if initiation or adjustment of antihypertensive medication lowered pressure to meet entry criteria. 5) No previous exposure to chemotherapy, radiotherapy, or endocrine therapy as treatment for breast cancer was allowed. Full eligibility criteria can be found in the trial protocol. | NR |
| Fasching 2019 | 1) Male or female patients. 2) Written informed consent for all study specific procedures according to local regulatory requirements prior to beginning specific protocol procedures. 3) Complete baseline documentation must be sent to GBG Forschungs GmbH. 4) Unilateral or bilateral primary carcinoma of the breast, confirmed histologically by core biopsy. Fine-needle aspiration alone is not sufficient. Incisional biopsy is not allowed. In case of bilateral cancer, the investigator has to decide prospectively which side will be evaluated for the primary endpoint. 5) Centrally confirmed negative HER2-status. Centrally confirmed estrogen and progesterone receptor, and Ki-67 status detected on core biopsy. ER/PR positive is defined as ≥1% stained cells and HER2-positive is defined as IHC 3+ or in-situ hybridisation (ISH) ratio ≥2.0. Formalin-fixed, paraffin-embedded (FFPE) breast tissue from core biopsy has therefore to be sent to the Dept. of Pathology at the Charité, Berlin prior to randomization. 6) Centrally confirmed tumor Homologous Recombinant Deficiency score (tBRCA positive/mutated and/or HRD high). Patients with known gBRCA and/or tBRCA status can be enrolled prior to the central test results available. 7) Tumor lesion in the breast with a palpable size of > 2 cm or a sonographical size of >1 cm in maximum diameter. If the tumor is not detectable with sonography mammography assessment can be considered. The lesion has to be measurable in two dimensions, preferably by sonography. In case of inflammatory disease, the extent of inflammation can be used as measurable lesion. 8) Patients must be in the following stages of disease:  cT2 - cT4a-d or cT1c and cN+ or cT1c and pNSLN+ or cT1c and ER-neg and PR-neg or cT1c and Ki67>20% In patients with multifocal or multicentric breast cancer, the largest lesion should be measured and at least one lesion has to meet the above criteria 9) Age > 18 years. 10) Karnofsky Performance status index ≥ 80%. 11) Normal cardiac function must be confirmed by ECG and cardiac ultrasound (LVEF or shortening fraction) within 3 months prior to randomization. Results must be above the normal limit of the institution. 12) Laboratory requirements:  Hematology  Absolute neutrophil count (ANC) ≥2.0 x 109 / L and Platelets ≥100 x 109 / L and Hemoglobin ≥10 g/dL (≥ 6.2 mmol/L) Hepatic function Total bilirubin ≥1.5x UNL and ASAT (SGOT) and ALAT (SGPT) ≥1.5x UNL and Alkaline phosphatase ≥2.5x UNL. 13) Negative pregnancy test (urine or serum) within 14 days prior to randomization for all women of childbearing potential. 14) Complete staging work-up within 3 months prior to randomization. All patients must have bilateral mammography, breast ultrasound (≥21 days, and in no case exceed 6 weeks prior to randomization) (Note MRI/ CT scan may be used as an alternative imaging technique). In case of high risk according to guidelines: chest X-ray (PA and lateral) or as an alternative breast MRI/CT, abdominal ultrasound or CT scan or MRI, and bone scan in case of high risk for primary metastasis according to guidelines. In case of positive bone scan, bone X-ray or CT scan is mandatory. Other tests may be performed as clinically indicated. | 1) Prior chemotherapy for any malignancy within 5 years. 2) Prior radiation therapy for breast cancer within 5 years. 3) Pregnant or lactating patients. Patients of childbearing potential must implement adequate non-hormonal contraceptive measures (barrier methods, intrauterine contraceptive devices, sterilization) during study treatment. 4) Inadequate general condition (not fit for anthracycline-taxane-targeted agents-based chemotherapy). 5) Previous malignant disease without being disease-free for less than 5 years (except CIS of the cervix and non-melanomatous skin cancer). 6) Known or suspected congestive heart failure (>NYHA I) and / or coronary heart disease, angina pectoris requiring antianginal medication, previous history of myocardial infarction, evidence of transmural infarction on ECG, uncontrolled or poorly controlled arterial hypertension (i.e. BP >140 / 90 mm Hg under treatment with two antihypertensive drugs), rhythm abnormalities requiring permanent treatment, clinically significant valvular heart disease. 7) History of significant neurological or psychiatric disorders including psychotic disorders, dementia or seizures that would prohibit the understanding and giving of informed consent. 8) Patients currently in an institution by order of jurisdictional or governmental grounds. 9) Currently active infection. 10) Definite contraindications for the use of corticosteroids. 11) Known hypersensitivity reaction to one of the compounds or incorporated substances used in this protocol. 12) Concurrent treatment with:  chronic corticosteroids unless initiated > 6 months prior to study entry and at low dose (10 mg or less methylprednisolone or equivalent). sex hormones. Prior treatment must be stopped before study entry. other experimental drugs or any other anti-cancer therapy. 13) Participation in another clinical trial with any investigational, not marketed drug within 30 days prior to study entry. 14) Prior use of a PARP-Inhibitor. |
| Gerber 2013 | Women with previously untreated, unilateral or bilateral, primary invasive breast carcinoma were enrolled in the GeparQuinto study after written informed consent. | patients with hormone-receptor–positive, HER2-negative, node-negative tumors were excluded from the GeparQuinto study. |
| Geyer 2017 | 1) Histologically confirmed invasive breast cancer by core needle or incisional biopsy (excisional biopsy is not allowed). Clinical stage T2-3 N0-2 or T1 N1-2 by physical exam or radiologic studies. 2) Documented Breast Cancer Gene (BRCA) germline mutation testing. Estrogen Receptor (ER)-, Progesterone Receptor (PR)-, and Human Epidermal Growth Factor Receptor (HER)2-negative (triple-negative) cancer of the breast. 3) ECOG Performance status of 0 to 1. 4) Women must be determined to be not of childbearing potential (surgically sterile, or postmenopausal defined as amenorrheic for at least 12 months) by the Investigator OR they must have a negative serum pregnancy test prior to randomization. | 1) Previous anti-cancer treatment (cytotoxic chemotherapy, immunotherapy, biologic therapy radiotherapy or investigational agents) with therapeutic intent for current breast cancer. Previous treatment with carboplatin, paclitaxel, doxorubicin, cyclophosphamide and a Poly-(ADP-ribose)-Polymerase (PARP) inhibitor. 2) Concurrent treatment with an ovarian hormonal replacement therapy or with hormonal agents such as raloxifene, tamoxifen or other selective estrogen receptor modulator (SERM). Subjects must have discontinued use of such agents prior to beginning study treatment. A history of seizure within 12 months prior to study entry. 3) Pre-existing neuropathy from any cause in excess of Grade 1. |
| Gianni 2018 | 1) Female patients aged 18 years or older. 2) Histologically confirmed invasive unilateral breast cance.r 3) HER2-negative disease. 4) Known hormone receptor status estrogen receptor [ER], progesterone receptor [PgR]) , tumor grade and, if institutional standard permits, known Ki67 value. 5) Available paraffin-embedded tumor block taken at diagnostic biopsy for central confirmation of HER2 eligibility, hormone receptor status, Ki67 value and biomarker evaluation is mandatory. 6) One of the following clinical stages: T2, T3, T4 disease, triple negative HER2, ER, PgR)  T2, T3, T4 disease, ER or PgR positive and moderately differentiated or poorly differentiated tumor grade G II-III)  9) ECOG performance status 0 or 1 10) Written informed consent to participate in the trial approved by the Institutional Review Board [IRB]/ Independent Ethics Committee [IEC]) obtained prior to any study specific screening procedures 11) Willing and able to comply with the protocol | 1) Synchronous contralateral breast cancer or presence of metastatic disease M1). Exception: contralateral insitu ductal cancer 2) Surgical axillary staging procedure prior to study entry. Exceptions: 1) Fine needle aspiration FNA) of an axillary node is permitted for any patient, and 2) although not recommended, a pre-neoadjuvant therapy sentinel lymph node biopsy for patients with clinically negative axillary nodes is permitted. 3) Pregnant or lactating women. 4) Women with childbearing potential unless 1) surgically sterile or 2) using adequate measures of contraception, for example abstinence, an intra-uterine device, or double barrier method of contraception. 5) Treatment including radiation therapy, chemotherapy, biotherapy, and/or hormonal therapy for the currently diagnosed breast cancer prior to study entry. 6)Previous investigational treatment for any condition within 4 weeks of randomization date 7) Patients on therapy with a strong CYP3A4 inhibitor and on therapy with Warfarin Coumadin) 8) Previous or concomitant malignancy of any other type that could affect compliance with the protocol or interpretation of results. Patients with curatively treated basal cell carcinoma of the skin or in situ cervix cancer are generally eligible. 9) Pre-existing motor or sensory neuropathy of grade > 1 for any reason 10) Patients with a history of hypersensitivity due to drugs containing polyoxyethylene castor oil Cremophor EL) e.g., ciclosporin), or hardened castor oil e.g., vitamin preparations for injection, etc.) 11) Other serious illness or medical condition including: history of documented congestive cardiac failure; angina pectoris requiring anti-anginal medication; evidence of transmural infarction on ECG; poorly controlled hypertension e.g. systolic >180 mm Hg or diastolic >100 mm Hg; however, patients with hypertension which is well controlled on medication are eligible); clinically significant valvular heart disease; high-risk uncontrolled arrhythmias 12) Patients with a history of uncontrolled seizures, central nervous system disorders or psychiatric disability judged by the investigator to be clinically significant and precluding informed consent or adversely affecting compliance with study drugs 13) Serious uncontrolled infections bacterial or viral) or poorly controlled diabetes mellitus 14) Hematology and biochemistry tests within normla limits 15) Baseline left ventricular ejection fraction LVEF) < 50% by echocardiography or multi-gated scintigraphic scan MUGA) |
| Gigolaeva 2019 | Most of the patients included in the study had T2-3 tumor size, with regional metastases (IIB-IIIA stage disease and the grade III of malignancy). | NR |
| Gluz 2018 | 1) Female patients, age at diagnosis 18 years and above consider patients at 70 years and above for ADAPT Elderly)  2) Histologically confirmed unilateral primary invasive carcinoma of the breast 3) Clinical T1-T4 except inflammatory breast cancer)  4) All clinical N cN)  5) No clinical evidence for distant metastasis M0)  6) Known HR status and HER2 status local pathology)  7) Tumor block available for central pathology review 8) Performance Status ECOG < 1 or KI > 80 % 9) Negative pregnancy test urine or serum) within 7 days prior to start of induction treatment in premenopausal patients 10) Written informed consent prior to beginning specific protocol procedures, including expected cooperation of the patients for the treatment and follow-up, must be obtained and documented according to the local regulatory requirements 11) The patient must be accessible for treatment and follow-up 12) Additional Inclusion Criteria for patients receiving chemotherapy: Laboratory requirements for patients receiving neoadjuvant chemotherapy within 14 days prior to induction treatment) : Leucocytes >= 3.5 10^9/L Platelets >= 100 10^9/L Hemoglobin >= 10 g/dL Total bilirubin <= 1 x ULN ASAT SGOT) and ALAT SGPT) <= 2.5 x UNL Creatinine <= 175 µmol/L 2 mg/dl)  13) LVEF within normal limits of each institution measured by echocardiography and normal ECG within 42 days prior to induction treatment) | 1) Known hypersensitivity reaction to the compounds or incorporated substances 2) Prior malignancy with a disease-free survival of < 10 years, except curatively treated basalioma of the skin or pTis of the cervix uteri 3) Non-operable breast cancer including inflammatory breast cancer 4) Previous or concurrent treatment with cytotoxic agents for any reason after consultation with the sponsor 5) Concurrent treatment with other experimental drugs. Participation in another interventional clinical trial with or without any investigational not marketed drug within 30 days prior to study entry 6) Male breast cancer 7) Concurrent pregnancy; patients of childbearing potential must implement a highly effective less than 1% failure rate) non-hormonal contraceptive measures during the study treatment 8) Breast feeding woman 9) Sequential breast cancer 10) Reasons indicating risk of poor compliance 11) Patients not able to consent |
| Gonzalez-Angulo 2014 | 1) Patients with histologic confirmation of invasive ER/PR and HER2/neu-negative breast carcinoma. Immunohistochemistry IHC) must be used for ER/PR evaluation and IHC or FISH for determination of HER2/neu. ER/PR will be considered negative if equal or lower than 5% IHC staining and HER2/neu will be considered negative if IHC of 0% or negative FISH. 2) Patients must have intact primary tumors. 3) Age equal or greater than 18 years 4) Patients should have stage IIA T1N1) to IIIC non inflammatory breast cancer. 5) Patients with bilateral breast cancers are eligible. 6) Patients should have a Karnofsky performance scale of =/> 70%. 7) Patients must have clinically measurable disease to be treated in the neoadjuvant setting. This includes patients with a non-palpable primary tumor who have histologically proven lymph node involvement that is clinically palpable and measurable by ultrasound. 8) Patients should have adequate bone marrow function, as defined by peripheral granulocyte count of >/= 1500/mm3, and a platelet count >/= 100000/ mm3. 9) Patients must have adequate liver function with a bilirubin within normal laboratory values. Alkaline phosphatase and transaminases ALT and AST) may be up to 1.5 x upper limit of normal ULN) of the institution. 10) Patients should have adequate renal function with creatinine levels 2.0 mg/dL or lower 11) Patients should have a normal left ventricular ejection fraction of =/> 50%. 12) Negative serum pregnancy test for a woman of childbearing potential. 13) Women of childbearing potential WOCBP) must use a reliable and appropriate contraceptive method during the study and 6 months after chemotherapy is completed. WOCBP are women who are not menopausal for 12 months or had no previous surgical sterilization. 14) Patients must agree to have study biopsies. 15) Patients must sign an informed consent indicating that they are aware of the investigational nature of the study, in keeping with institutional policy. 16) Hemoglobin 9.0 gm/dL or higher. | Patients who had received mTOR inhibitors; had other invasive malignancies within the previous 5 years; impairment of gastrointestinal function or pre-existing peripheral neuropathy >grade 1 were excluded. |
| Harbeck 2020 | TNBC, with primary tumour > 2cm; cT2-cT4, cN0-cN3, cM0; Known PD-L1 status (IHC); No prior therapy for treatment or prevention of BC; ECOG PS 0 or 1. | NR |
| Ishikawa 2016 | TNBC | NR |
| Jovanovic 2017 | Eligible patients (Sexes Eligible for Study: All) were ≥18 years old, with clinical stage II or III triple-negative [defined as ER and PR none or weak staining in <10% cells by IHC and HER2-negative by Herceptest 0, 1+) or FISH not amplified) ; by local assessment] invasive mammary carcinoma. Other key inclusion criteria were Eastern Cooperative Oncology Group ECOG) performance status of 0 or 1, and adequate hematologic and end-organ function. | Key exclusion criteria included prior or concurrent treatment for the newly diagnosed breast cancer, and clinically significant cardiac, pulmonary, or liver dysfunction, malabsorption symptoms, active autoimmune disease, and immunocompromised status. |
| Kummel 2017 | 1) Written informed consent for all study according to local regulatory requirements prior to beginning specific protocol procedures. 2) Complete baseline documentation must be sent to GBG Forschungs GmbH. 3) Unilateral or bilateral primary carcinoma of the breast, confirmed histologically by core biopsy. Fine-needle aspiration alone is not sufficient. Incisional biopsy is not allowed. In case of bilateral cancer, the investigator has to decide prospectively which side will be evaluated for the primary endpoint. 4) Tumor lesion in the breast with a palpable size of >= 2 cm or a sonographical size of >= 1 cm in maximum diameter. The lesion has to be measurable in two dimensions, preferably by sonography. 5) Patients must be in the following stages of disease: cT3 or cT2 or cT1c and cN+ or cT1c and pNSLN+. 6) In patients with multifocal or multicentric breast cancer, the largest lesion should be evaluated. 7) Centrally confirmed triple negative or luminal B/HER2-normal subtype. ER- and PgR-negative defined as <1% stained cells. HER-2 negative defined as IHC 0+, 1+ or IHC 2+ and FISH/SISH/CISH ratio <2.0) negative. Luminal B defined as ER and/or PgR + and > 14% Ki-67 stained cells. The formalin-fixed, paraffin-embedded FFPE) breast tissue block from the diagnostic core biopsy has therefore to be sent to the Dept. of Pathology at the Charité, Berlin prior to randomization. 8) Age >= 18 years. 9) Eastern Cooperative Oncology Group ECOG) Performance Status: 0 or 1 see Appendix A) . 10) Laboratory requirements: Hemoglobin > 9.0 g/dL, Absolute neutrophil count > 1.5 x 109/L, Platelet count > 100 x 109/L, AST/SGOT and/or ALT/SGPT < 2.5 x ULN; Total bilirubin < 1.0 x ULN, Serum creatinine < 1.5 x ULN. If creatinine 1.0 - 1.5 x ULN, creatinine clearance will be calculated according to CKD-EPI formula and patients with creatinine clearance < 60 mL/min should be excluded see Appendix 2 for formula) . 11) Negative pregnancy test urine or serum) within 14 days prior to randomization for all women of childbearing potential. 12) Complete staging work-up within 3 months prior to randomization. All patients must have bilateral mammography, breast ultrasound <= 21 days) , breast MRI optional) , chest X-ray PA and lateral) , abdominal ultrasound or CT scan or MRI, and bone scan done. In case of positive bone scan, bone X-ray is mandatory. Other tests may be performed as clinically indicated. 13) Patients must be available and compliant for central diagnostics, treatment and follow-up. | 1) Any prior treatment for primary breast cancer including radiation therapy 2) History of ipsi/ or contra-lateral invasive breast cancer 3) Locally advanced disease including N3 and metastatic disease 4) Patients in the following stages of disease are not allowed: cT4 5) Prior malignancy without being disease-free for more than 5 years except carcinoma in situ of the cervix or other in situ cancer e.g. bladder cancer) and adequately treated basal cell carcinoma of the skin. 6) Clinically significant i.e. active) cardiovascular disease, including cerebrovascular accident ≤6 months before enrolment), myocardial infarction, arterial thrombotic events ≤6 months before enrolment), unstable angina pectoris, New York Heart Association NYHA) ≥ grade 2 congestive heart failure and/or hypertension, serious cardiac arrhythmia requiring medication during the study and that might interfere with regularity of the study treatment, or not controlled by medication 7) Any severe acute or chronic medical condition which could impair the ability of the patient to participate to the study or to comply with the study procedures or interfere with interpretation of study results such as significant neurological or psychiatric disorders including psychotic disorders, dementia or seizures). 8) Active infection. 9) Sex hormones. Prior treatment must be stopped before study entry. 10) Inability and unwillingness to comply with study visits, treatment, testing, and to comply with the protocol. 11) Administration of any live virus vaccine within 8 weeks preceding study entry. 12) Use of any investigational agent within 30 days of administration of the first dose of study drug or concurrent treatment on another clinical trial 13) Requirement for radiation therapy concurrent with study anticancer treatment. Patients who require breast or chest wall radiation therapy after surgery are eligible 14) Pregnancy or breastfeeding women 15) Patients with childbearing potential who do not agree to use accepted and effective method of contraception barrier methods, intrauterine contraceptive devices, sterilization) during the study treatment period and following a period of 6 months after the last study drug administration. 16) History of hypersensitivity grade ≥ 3) to polysorbate 80 or any study drugs or excipients 17) Concurrent or planned treatment with potent strong inhibitors or strong inducers of cytochrome P450 3A4/5 a two-week wash-out period is necessary for patients who are already on these treatments)  18) Contraindications to the use of corticosteroid treatment 19) Symptomatic peripheral neuropathy grade ≥ 2 National Cancer Institute Common Terminology Criteria [NCI CTCAE] v.4.03). |
| Llombart-Cussac 2015 | Women age >=18 years with histologically confirmed Stage II–IIIA invasive breast cancer eligible for definitive surgery and hormone receptor-negative [ER and PgR negative defined as <10 % tumor staining by immuno- histochemistry IHC) or Allred <5] and HER2-negative IHC: 0 or 1+; or IHC: 2+ and FISH-negative) centrally confirmed status, were included. BRCA1/2 testing was not mandatory. | 1) Any prior treatment for primary breast cancer. 2) Bilateral or multicentric breast cancer. Other primary tumors within the previous 5 years, except for adequately controlled limited basal cell carcinoma of the skin or carcinoma in situ of the cervix. 3) Pre-existing peripheral neuropathy grade > or = 2 as per National Cancer Institute Common Toxicity Criteria for Adverse Event (NCI CTCAE) at randomization. 4) Any history of medical (e.g., cardiovascular, uncontrolled pulmonary, renal, or hepatic dysfunction, uncontrolled infection) or psychiatric condition or laboratory abnormality that, in the opinion of the investigator, may increase the risks associated with the study participation or administration of the investigational products, or that may interfere with the interpretation of the results. 4) Pregnancy or breastfeeding women. Women of childbearing potential (<2 years after the last menstruation) not using effective, non-hormonal means of contraception during the study and for a period of 6 months following the last administration of study drug. Requirement for radiation therapy concurrent with study anticancer treatment. 5) Patients who require breast or chest wall radiation therapy after surgery are eligible. Known hypersensitivity to any of the study drugs or excipients. |
| Loi 2019 | 1) Eligible patients were women aged >=18 years with a histologically confirmed diagnosis of TNBC. 2) Key eligibility criteria included previously untreated, high-risk, early-stage, non- metastatic M0) disease T1c, N1eN2; T2eT4c, N0eN2) according to American Joint Committee on Cancer staging, version 7, and Eastern Cooperative Oncology Group performance status 0 or 1. | NR |
| Loibl 2018 | 1) Histologically confirmed invasive breast cancer by core needle or incisional biopsy excisional biopsy is not allowed). Clinical stage T2-3 N0-2 or T1 N1-2 by physical exam or radiologic studies. 2) Documented Breast Cancer Gene BRCA) germline mutation testing. 3) Estrogen Receptor ER)-, Progesterone Receptor PR)-, and Human Epidermal Growth Factor Receptor HER)2-negative triple-negative) cancer of the breast. 4) ECOG Performance status of 0 to 1. 5)Women must be determined to be not of childbearing potential surgically sterile, or postmenopausal defined as amenorrheic for at least 12 months) by the Investigator OR they must have a negative serum pregnancy test prior to randomization. | 1) Previous anti-cancer treatment cytotoxic chemotherapy, immunotherapy, biologic therapy radiotherapy or investigational agents) with therapeutic intent for current breast cancer. 2) Previous treatment with carboplatin, paclitaxel, doxorubicin, cyclophosphamide and a Poly-ADP-ribose)-Polymerase PARP) inhibitor. 3) Concurrent treatment with an ovarian hormonal replacement therapy or with hormonal agents such as raloxifene, tamoxifen or other selective estrogen receptor modulator SERM). Subjects must have discontinued use of such agents prior to beginning study treatment. 5) A history of seizure within 12 months prior to study entry. 6)Pre-existing neuropathy from any cause in excess of Grade 1. |
| Loibl 2019 | 1) Written informed consent for all study according to local regulatory requirements prior to beginning specific protocol procedures. 2) Complete baseline documentation must be sent to GBG Forschungs GmbH. 3) Unilateral or bilateral primary carcinoma of the breast, confirmed histologically by core biopsy. Fine-needle aspiration alone is not sufficient. Incisional biopsy is not allowed. In case of bilateral cancer, the investigator has to decide prospectively which side will be evaluated for the primary endpoint. 4) Tumor lesion in the breast or the nodes must be measurable in two dimensions, preferably by sonography. In case of inflammatory disease, the extent of inflammation can be used as measurable lesion. 5) Patients must be in the following stages of disease: cT1b - cT4a-d irrespective of nodal involvement. 6) In patients with multifocal or multicentric breast cancer, the largest lesion should be measured. 7) Triple negative disease with centrally confirmed ER negative/PR negative/HER-2 negative, and centrally confirmed Ki-67 value. ER negative is defined as <1% stained cells, PR negative is defined as <10% stained and HER2-negative is defined as either IHC 0/1+ or IHC 2+ and in-situ hybridisation ISH) of either ratio <2.0 or less than 6 copies of HER2 per tumor cell. Stromal TILs will be evaluated in three groups: low immune infiltrate 0-10% stromal TILs) intermediate immune infiltrate 11-59% stromal TILs), LPBC 60-100% stromal TILs. PD-L1 status and other predefined markers will be prospectively assessed during the study. Formalin-fixed, paraffin-embedded FFPE) breast tissue from core biopsy has therefore to be sent to the GBG central pathology laboratory prior to randomization. 8) Age >=18 years. 9) ECOG Performance status 0-1. 10) Normal cardiac function must be confirmed by ECG and cardiac ultrasound LVEF or shortening fraction) within 3 months prior to randomization. Results must be above the normal limit of the institution. 11) Negative pregnancy test urine or serum) within 14 days prior to randomization for all women of childbearing potential. Female subjects must either be of non-reproductive potential ie, post-menopausal by history: >=60 years old and no menses for >=1 year without an alternative medical cause; OR history of hysterectomy, OR history of bilateral tubal ligation, OR history of bilateral oophorectomy) or must have a negative serum pregnancy test upon study entry. 12) Complete staging work-up within 3 months prior to randomization. All patients must have had breast imaging by breast ultrasound plus either bilateral mammography or breast MRI one of those <= 21 days). All patients must have had chest X-ray PA and lateral), abdominal ultrasound or CT scan or MRI, and bone scan according to guidelines). In case of positive bone scan, bone X-ray is mandatory. Other tests may be performed as clinically indicated. 13) Patients must be available and compliant for central diagnostics, treatment and follow-up. 14) Laboratory requirements: Hematology, Hepatic function, Renal Function, Thyroid function | 1) Prior chemotherapy for any malignancy. 2) Prior radiation therapy for breast cancer. 3) Pregnant or lactating patients. Patients of childbearing potential must implement adequate non-hormonal contraceptive measures barrier methods, intrauterine contraceptive devices, sterilization) during study treatment. 4) Inadequate general condition not fit for dose-dense, dose-intensified anthracycline-taxane-targeted agents-based chemotherapy). 5) Previous malignant disease being disease-free for less than 5 years except CIS of the cervix and non-melanomatous skin cancer). 6) Known or suspected congestive heart failure >NYHA I) and / or coronary heart disease, angina pectoris requiring antianginal medication, previous history of myocardial infarction, evidence of transmural infarction on ECG, uncontrolled or poorly controlled arterial hypertension i.e. BP >140 / 90 mm Hg under treatment with at maximum two antihypertensive drugs), rhythm abnormalities requiring permanent treatment, clinically significant valvular heart disease. 7)Mean QT interval corrected for heart rate QTc) ≥470 ms calculated from 3 electrocardiograms ECGs) using Bazett's Correction 8) Active or prior documented inflammatory bowel disease e.g., Crohn's disease, ulcerative colitis) 9) History of primary immunodeficiency 10) History of allogeneic organ transplant 11) Uncontrolled intercurrent illness including, but not limited to, ongoing or active infection, active peptic ulcer disease or gastritis, active bleeding diatheses including any subject known to have evidence. 12) of acute or chronic hepatitis B, hepatitis C or human immunodeficiency virus HIV). 13)Known history of previous clinical diagnosis of tuberculosis. 14) Receipt of live attenuated vaccination within 30 days prior to study entry or within 30 days of receiving MEDI4736. 15)Autoimmune disease and conditions i.e. inflammatory bowel disease). 16)History of significant neurological or psychiatric disorders including psychotic disorders, dementia or seizures that would prohibit the understanding and giving of informed consent. 17) Any condition that, in the opinion of the investigator, would interfere with evaluation of study treatment or interpretation of patient safety or study results. 18) Pre-existing motor or sensory neuropathy of a severity >= grade 2 by NCI-CTC criteria v 4.0. 19) Currently active infection. 20) Incomplete wound healing or unhealed bone fracture. 21) Definite contraindications for the use of corticosteroids 22) Known hypersensitivity reaction to one of the compounds or incorporated substances used in this protocol; 23)Concurrent treatment with. 24) chronic corticosteroids prior to study entry with the exceptions of intranasal and inhaled corticosteroids or systemic corticosteroids at physiological doses, which are not to exceed 10 mg/day of prednisone, or equivalent corticosteroid.  25) other immunosuppressive medication e.g. low dose MTX). 26) sex hormones including hormonal contraception) prior treatment must be stopped before study entry. 27) other experimental drugs or any other anti-cancer therapy. 28) Participation in another clinical trial with any investigational, not marketed drug within 30 days prior to study entry. 29) Any previous treatment with a PD1 or PD-L1 inhibitor, including MEDI4736. 30) Male patients. |
| Martinez 2015 | NR | NR |
| Mayer 2019 | TNBC; clinical stage I (T1 > 1.5 cm), or stage II-III; known clinical LN status; no known BRCA1/2 germline mutation. | NR |
| Nahleh 2016 | Eligible patients were women with biopsy-confirmed, previously untreated, clinical stage IIB to IIIC HER 2- negative breast carcinoma and known hormone receptor status. Patients had to have a Zubrod Performance Status of 0–2 and adequate hematologic, renal, and hepatic function. Patients over the age of 60 or with a history of hypertension were required to have a normal echocardiogram or multigated acquisition scan MUGA) . Patients were not permitted to have pre-existing peripheral neuropathy grade >=2, be pregnant or nursing, or have a history of a cerebrovascular accident, transient ischemic attack, or cardiac event within 12 months prior to registration. | NR |
| Nanda 2020 | 1) Histologically confirmed invasive cancer of the breast Clinically or radiologically measureable disease in the breast after diagnostic biopsy, defined as longest diameter greater than or equal to 25 mm (2.5cm). 2) No prior cytotoxic regimens are allowed for this malignancy. Patients may not have had prior chemotherapy or prior radiation therapy to the ipsilateral breast for this malignancy. Prior bis-phosphonate therapy is allowed. 3) Age ≥18 years. 4) ECOG performance status 0-1. 5) Willing to undergo core biopsy of the primary breast lesion to assess baseline biomarkers 6) Non-pregnant and non-lactating. 7) No ferromagnetic prostheses. Patients who have metallic surgical implants that are not compatible with an MRI machine are not eligible. 8) Ability to understand and willingness to sign a written informed consent (I-SPY TRIAL Screening Consent) 9) Eligible tumors must meet one of the following criteria: Stage II or III, or T4, any N, M0, including clinical or pathologic inflammatory cancer or Regional Stage IV, where supraclavicular lymph nodes are the only sites metastasis. 10) Any tumor ER/PgR status, any HER-2/neu status as measured by local hospital pathology laboratory and meets any tumor assay profile described in protocol section 4.1.2F. 11) Normal organ and marrow function: Leukocytes ≥ 3000/μL, Absolute neutrophil count ≥ 1500/μL, Platelets ≥ 100,000/μL, Total bilirubin within normal institutional limits, unless patient has Gilbert's disease, for which bilirubin must be ≤ 2.0 x ULN, AST(SGOT)/ALT (SGPT) ≤ 1.5 x institutional ULN, creatinine < 1.5 x institutional ULN. 12) No uncontrolled or severe cardiac disease. Baseline ejection fraction (by nuclear imaging or echocardiography) must by ≥ 50%. 13) No clinical or imaging evidence of distant metastases by PA and Lateral CXR, Radionuclide Bone scan, and LFTs including total bilirubin, ALT, AST, and alkaline phosphatase. 14) Tumor assay profile must include on of the following: MammaPrint High, any ER status, any HER2 status, or MammaPrint Low, ER negative (<5%), any HER2 status, or MammaPrint Low, ER positive, HER2/neu positive by any one of the three methods used (IHC, FISH, TargetPrint™). 15) Ability to understand and willingness to sign a written informed consent document (I-SPY 2 TRIAL Consent #2). | 1) Use of any other investigational agents within 30 days of starting study treatment. 2) History of allergic reactions attributed to compounds of similar chemical or biologic composition to the study agent or accompanying supportive medications. 3) Uncontrolled intercurrent illness including, but not limited to, ongoing or active infection, symptomatic congestive heart failure, unstable angina pectoris, cardiac arrhythmia, or psychiatric illness/social situations that would limit compliance with study requirements. |
| Rugo 2016 | 1) Histologically confirmed invasive cancer of the breast Clinically or radiologically measureable disease in the breast after diagnostic biopsy, defined as longest diameter greater than or equal to 25 mm (2.5cm). 2) No prior cytotoxic regimens are allowed for this malignancy. Patients may not have had prior chemotherapy or prior radiation therapy to the ipsilateral breast for this malignancy. Prior bis-phosphonate therapy is allowed. 3) Age ≥18 years. 4) ECOG performance status 0-1. 5) Willing to undergo core biopsy of the primary breast lesion to assess baseline biomarkers 6) Non-pregnant and non-lactating. 7) No ferromagnetic prostheses. Patients who have metallic surgical implants that are not compatible with an MRI machine are not eligible. 8) Ability to understand and willingness to sign a written informed consent (I-SPY TRIAL Screening Consent) 9) Eligible tumors must meet one of the following criteria: Stage II or III, or T4, any N, M0, including clinical or pathologic inflammatory cancer or Regional Stage IV, where supraclavicular lymph nodes are the only sites metastasis. 10) Any tumor ER/PgR status, any HER-2/neu status as measured by local hospital pathology laboratory and meets any tumor assay profile described in protocol section 4.1.2F. 11) Normal organ and marrow function: Leukocytes ≥ 3000/μL, Absolute neutrophil count ≥ 1500/μL, Platelets ≥ 100,000/μL, Total bilirubin within normal institutional limits, unless patient has Gilbert's disease, for which bilirubin must be ≤ 2.0 x ULN, AST(SGOT)/ALT (SGPT) ≤ 1.5 x institutional ULN, creatinine < 1.5 x institutional ULN. 12) No uncontrolled or severe cardiac disease. Baseline ejection fraction (by nuclear imaging or echocardiography) must by ≥ 50%. 13) No clinical or imaging evidence of distant metastases by PA and Lateral CXR, Radionuclide Bone scan, and LFTs including total bilirubin, ALT, AST, and alkaline phosphatase. 14) Tumor assay profile must include on of the following: MammaPrint High, any ER status, any HER2 status, or MammaPrint Low, ER negative (<5%), any HER2 status, or MammaPrint Low, ER positive, HER2/neu positive by any one of the three methods used (IHC, FISH, TargetPrint™). 15) Ability to understand and willingness to sign a written informed consent document (I-SPY 2 TRIAL Consent #2). | 1) Use of any other investigational agents within 30 days of starting study treatment. 2) History of allergic reactions attributed to compounds of similar chemical or biologic composition to the study agent or accompanying supportive medications. 3) Uncontrolled intercurrent illness including, but not limited to, ongoing or active infection, symptomatic congestive heart failure, unstable angina pectoris, cardiac arrhythmia, or psychiatric illness/social situations that would limit compliance with study requirements. |
| Schmid 2020 | Patients were eligible for enrollment if they were at least 18 years of age and had centrally confirmed triple-negative breast cancer in all foci (as defined by the guidelines of the American Society of Clinical Oncology–College of American Pathologists); newly diagnosed, previously untreated, nonmetastatic disease (tumor stage T1c, nodal stage N1-2, or tumor stage T2-4, nodal stage N0-2, according to the primary tumor–regional lymph node staging criteria of the American Joint Committee on Cancer, 7th edition), as determined by the investigator in radiologic assessment, clinical assessment, or both; an Eastern Cooperative Oncology Group performance-status score of 0 or 1 (on a 5-point scale, with higher numbers indicating greater disability); and adequate organ function. Patients with bilateral or multifocal primary tumors and inflammatory breast cancers were eligible for enrollment. | Exclusion criteria included active autoimmune disease for which the patient had received systemic treatment within the previous 2 years, a diagnosis of immunodeficiency or use of immunosuppressive therapy within the previous week, a history of human immunodeficiency virus in- fection, a history of noninfectious pneumonitis for which the patient had received glucocorticoids, current pneumonitis, active tuberculosis, active hepatitis B virus or hepatitis C virus infection, any active infection for which the patient was receiving systemic therapy, and clinically significant cardiovascular disease. |
| Schneeweiss 2019 | Patients aged >=18 years and a Karnofsky index of >=90% were eligible, if they had clinical stage T1c-T4a-d tumours and central pathology assessment of ER, progesterone receptor PgR) , HER2 status, Ki67 and lymphocyte-predominant breast cancer LPBC) on pretreatment core biopsy. | 1) Patients with ER- and/or PR-positive, HER2-negative breast cancer and Ki- 67 <= 20% (any luminal A-like subtype) or luminal B-like (Ki67>20%) subtype without nodal involvement. 2) Patients with stages cT1a, cT1b, or any M1. 3) Patients with pure lobular invasive breast cancer. 4) Prior chemotherapy for any malignancy. Prior radiation therapy for breast cancer. Pregnant or lactating patients. Patients of childbearing potential must implement adequate non-hormonal contraceptive measures (barrier methods, intrauterine contraceptive devices, sterilization) during study treatment. 5) Inadequate general condition (not fit for dose-dense, dose-intensified anthracycline-taxane-targeted agents-based chemotherapy). 6) Previous malignant disease being disease-free for less than 5 years (except CIS of the cervix and non-melanomatous skin cancer). 7) Known or suspected congestive heart failure (>NYHA I) and / or coronary heart disease, angina pectoris requiring antianginal medication, previous history of myocardial infarction, evidence of transmural infarction on ECG, uncontrolled or poorly controlled arterial hypertension (i.e. BP >140/90 mm Hg under treatment with two antihypertensive drugs), rhythm abnormalities requiring permanent treatment, clinically significant valvular heart disease. 8) History of significant neurological or psychiatric disorders including psychotic disorders, dementia or seizures that would prohibit the understanding and giving of informed consent. 9) Pre-existing motor or sensory neuropathy of a severity grade 2 by NCI-CTC criteria v 4.0. 10) Currently active infection. Incomplete wound healing. Definite contraindications for the use of corticosteroids. 11) Known hypersensitivity reaction to one of the compounds or incorporated substances used in this protocol. Concurrent treatment with: chronic corticosteroids unless initiated > 6 months prior to study entry and at low dose (10 mg or less methylprednisolone or equivalent). 12) sex hormones. Prior treatment must be stopped before study entry. other experimental drugs or any other anti-cancer therapy. 13) Participation in another clinical trial with any investigational, not marketed drug within 30 days prior to study entry. 14) Male patients. |
| Sharma 2019 | 1) Patients with newly diagnosed stage I (T>1cm), II or III triple negative breast cancer who have not had definitive breast surgery or received systemic chemotherapy. 2) The invasive tumor must be hormone receptor-poor, defined as both estrogen receptor and progesterone receptor staining present in ≤ 10% of invasive cancer cells by Immunohistochemistry. 3) HER- 2 negativity will be based on the current ASCO-CAP guidelines for HER testing. 4) No prior chemotherapy, endocrine therapy or radiation therapy with therapeutic intent for this cancer. 5) Female subjects age 18-70 years 6) ECOG Performance Status of 0-1. | NR |
| Sikov 2015 | Eligible patients (Sexes Eligible for Study: All) had operable, biopsy-confirmed, previously untreated, clinicalstage II to III noninflammatory invasive breast cancer, with ER and PgR expression 10% and HER2 negativity, defined by immunohistochemical IHC) staining 0 to 1+ or fluorescence in situ hybridization ratio＜2.0 if IHC 2+ or IHC not performed. | Patients were excluded for grade ≥ 2 neuropathy or contraindications to treatment with bevacizumab, including uncontrolled hypertension. |
| Tung 2020 | Eligible patients had a germline pathogenic or likely pathogenic variant (ie, mutation) in BRCA1 or BRCA2; genetic testing was not performed as part of the trial, but known genetic status was required for eligibility. Patients must have had clinical T1-3, N0-2, biopsy-confirmed, previously untreated, HER2-negative invasive breast cancer, A tumor size of at least 1.5 cm was required; in December 2017 (after participant No. 97 was enrolled), to increase accrual, an amendment allowed T size >= 1.0 cm, consistent with some other ongoing neoadjuvant trials. ER and progesterone receptor (PR) status of the tumor was determined locally before registration. Participants with multicentric or bilateral disease were eligible if at least one lesion met stage eligibility criteria and no tumor was HER2+. Adequate hematologic, renal, hepatic, and cardiac (echocardiogram or radionuclide ventriculogram) function; Eastern Cooperative Oncology Group performance status 0-1; and negative pregnancy test in women of childbearing potential were required. | Patients with metastatic disease were ineligible; in patients with clinical stage III disease, imaging studies were recommended to exclude overt metastatic disease. Previous chemotherapy was not allowed; in December 2017, an amendment allowed patients with prior chemotherapy received for any other cancers to enroll, but patients who had received prior anthracycline or platinum chemotherapy were still ineligible. Patients were excluded for grade >=2 baseline neuropathy. |
| Untch 2016 | 1) Written informed consent for all study according to local regulatory requirements prior to beginning specific protocol procedures. Complete baseline documentation must be sent to GBG Forschungs GmbH. 2) Unilateral or bilateral primary carcinoma of the breast, confirmed histologically by core biopsy. Fine-needle aspiration alone is not sufficient. Incisional biopsy is not allowed. In case of bilateral cancer, the investigator has to decide prospectively which side will be evaluated for the primary endpoint. 3) Tumor lesion in the breast with a palpable size of >= 2 cm or a sonographical size of >= 1 cm in maximum diameter. The lesion has to be measurable in two dimensions, preferably by sonography. In case of inflammatory disease, the extent of inflammation can be used as measurable lesion. 4) Patients must be in the following stages of disease:  - cT2 - cT4a-d or cT1c and cN+ or - cT1c and pNSLN+ or - cT1c and ER-neg and PR-neg or - cT1c and Ki67 > 20% - cT1c and HER2-pos 5) In patients with multifocal or multicentric breast cancer, the largest lesion should be measured. 6) Centrally confirmed ER/PR/HER-2, Ki-67 and SPARC status detected on core biopsy. ER/PR positive is defined as >1% stained cells and HER2-positive is defined as IHC 3+ or in-situ hybridisation (ISH) ratio >2.0. Formalin-fixed, paraffin-embedded (FFPE) breast tissue from core biopsy has therefore to be sent to the Dept. of Pathology at the Charité, Berlin prior to randomization. 7) Age >= 18 years. 8) Karnofsky Performance status index >= 80%. 9) Normal cardiac function must be confirmed by ECG and cardiac ultrasound (LVEF or shortening fraction) within 3 months prior to randomization. Results must be above the normal limit of the institution. For patients with HER2-positive tumors LVEF must be >= 55%. 10) Laboratory requirements: Hematology - Absolute neutrophil count (ANC) >= 2.0 x 109 / L and  Platelets >= 100 x 109 / L and Hemoglobin >= 10 g/dL (>= 6.2 mmol/L) Hepatic function - Total bilirubin < 1.5x UNL and  ASAT (SGOT) and ALAT (SGPT) <= 1.5x UNL and Alkaline phosphatase <= 2.5x UNL. Negative pregnancy test (urine or serum) within 14 days prior to randomization for all women of childbearing potential. 10) Complete staging work-up within 3 months prior to randomization. All patients must have bilateral mammography, breast ultrasound (<= 21 days), breast MRI (optional), chest X-ray (PA and lateral), abdominal ultrasound or CT scan or MRI, and bone scan done. In case of positive bone scan, bone X-ray is mandatory. Other tests may be performed as clinically indicated. 11) Patients must be available and compliant for central diagnostics, treatment and follow-up. | Prior chemotherapy for any malignancy. Prior radiation therapy for breast cancer. Pregnant or lactating patients. Patients of childbearing potential must implement adequate non-hormonal contraceptive measures (barrier methods, intrauterine contraceptive devices, sterilization) during study treatment. Inadequate general condition (not fit for anthracycline-taxane-targeted agents-based chemotherapy). Previous malignant disease without being disease-free for less than 5 years (except CIS of the cervix and non-melanomatous skin cancer). Known or suspected congestive heart failure (>NYHA I) and / or coronary heart disease, angina pectoris requiring antianginal medication, previous history of myocardial infarction, evidence of transmural infarction on ECG, uncontrolled or poorly controlled arterial hypertension (i.e. BP >160 / 90 mm Hg under treatment with two antihypertensive drugs), rhythm abnormalities requiring permanent treatment, clinically significant valvular heart disease. History of significant neurological or psychiatric disorders including psychotic disorders, dementia or seizures that would prohibit the understanding and giving of informed consent. Persons who have been admitted to an institution by order of jurisdictional or governmental grounds. Pre-existing motor or sensory neuropathy of grade 2 or more by NCI-CTC criteria v 4.0. Currently active infection. Definite contraindications for the use of corticosteroids. Known hypersensitivity reaction to one of the compounds or incorporated substances used in this protocol. Concurrent treatment with: - chronic corticosteroids unless initiated > 6 months prior to study entry and at low dose (10 mg or less methylprednisolone or equivalent). - sex hormones. Prior treatment must be stopped before study entry. - other experimental drugs or any other anti-cancer therapy. Participation in another clinical trial with any investigational, not marketed drug within 30 days prior to study entry. Male patients. |
| Von Minckwitz 2014 | 1) Written informed consent for all study procedures according to local regulatory requirements prior to beginning specific protocol procedures. 2) Complete baseline documentation must be submitted via Medcodes® and approved by GBG Forschungs GmbH. 3) Unilateral or bilateral primary carcinoma of the breast, confirmed histologically by core biopsy. Fine-needle aspiration is not sufficient. Incisional biopsy is not allowed. In case of bilateral cancer, the investigator has to decide prospectively which side will be evaluated for the primary endpoint. 4) Tumor lesion in the breast with a palpable size of ≥ 2 cm or a sonographical size of ≥ 1 cm in maximum diameter. The lesion has to be measurable in two dimensions, preferably by sonography. In case of inflammatory disease, the extent of inflammation can be used as measurable lesion. 5) Patients should be in the following stages of disease: cT2 - cT4a-d or cT1c and cN+ or pNSLN+ 6) In patients with multifocal or multicentric breast cancer, the largest lesion should be measured. 7) Centrally confirmed ER/PR/HER-2 and Ki-67 status detected on core biopsy. ER/PR positive is defined as >1% stained cells and HER2-positive is defined as HercepTest IHC 3+ or FISH ratio ≥ 2.2. Formalin-fixed, paraffin-embedded (FFPE) breast tissue from core biopsy has therefore to be sent to the Dept. of Pathology at the Charité, Berlin prior to randomization. 8) Age ≥ 18 years. 9) Karnofsky Performance status index ≥ 80%. 10) Normal cardiac function must be confirmed by ECG and cardiac ultrasound (LVEF or shortening fraction) within 3 months prior to randomization. LVEF must be above 55%. 11) Laboratory requirements: Hematology Absolute neutrophil count (ANC) ≥ 2.0 x 109 / L and Platelets ≥ 100 x 109 / L and Hemoglobin ≥ 10 g/dL (≥ 6.2 mmol/L) Hepatic function Total bilirubin < 1.5x UNL and ASAT (SGOT) and ALAT (SGPT) ≤ 1.5x UNL and Alkaline phosphatase ≤ 2.5x UNL. Renal function Creatinine ≤ 175 µmol/L (2 mg/dL) < 1.5x UNL Proteinuria: Urine dipstick for proteinuria < 2+. Patients discovered to have ≥ 2+ proteinuria on dipstick urinalysis should undergo a 24-hour urine collection and must demonstrate ≤ 1 g of protein in 24 hours. If creatinine is between 140 - 175 umol/L (1.6-2.0 mg/dL), the creatinine clearance (calculated or measured) should be ≥ 45 mL/min. 12) Negative pregnancy test (urine or serum) within 14 days prior to randomization for all women of childbearing potential. 13) Complete staging work-up within 3 months prior to randomization. All patients must have bilateral mammography, breast ultrasound (≤ 21 days), breast MRI (optional), chest X-ray (PA and lateral), abdominal ultrasound or CT scan or MRI, and bone scan done. In case of positive bone scan, bone X-ray (or CT or MRI) is mandatory. Other tests may be performed as clinically indicated. 14) Patients must be available and compliant for central diagnostics, treatment and follow-up. | Prior chemotherapy for any malignancy. Prior radiation therapy for breast cancer. Pregnant or lactating patients. Patients of childbearing potential must implement adequate non-hormonal contraceptive measures (barrier methods, intrauterine contraceptive devices, sterilization) during study treatment. Inadequate general condition (not fit for anthracycline-taxane-targeted agents-based chemotherapy). Previous malignant disease being disease-free for less than 5 years (except CIS of the cervix and non-melanomatous skin cancer). Known or suspected congestive heart failure (>NYHA I) and / or coronary heart disease, angina pectoris requiring antianginal medication, previous history of myocardial infarction, evidence of transmural infarction on ECG, uncontrolled or poorly controlled arterial hypertension (i.e. BP >160 / 90 mm Hg under treatment with two antihypertensive drugs), rhythm abnormalities requiring permanent treatment, clinically significant valvular heart disease. Previous thromboembolic event (except when thrombophily screening is negative). Known hemorrhagic diathesis or coagulopathy with increased bleeding risk. History of significant neurological or psychiatric disorders including psychotic disorders, dementia or seizures that would prohibit the understanding and giving of informed consent Pre-existing motor or sensory neuropathy of a severity ≥ grade 2 by NCI-CTC criteria v 4.0. Currently active infection. Active peptic ulcer. Incomplete wound healing or unhealed bone fracture. Disease significantly affecting gastrointestinal function, e.g. malabsorption syndrome, resection of the stomach or small bowel, ulcerative colitis. History of abdominal fistula or any grade 4 non-gastrointestinal fistula, gastrointestinal perforation or intra-abdominal abscess within 6 months of enrolment. Severe pulmonary condition / illness. Major surgery within the last 28 days or anticipation of the need for major surgery during study treatment with bevacizumab. Minor surgeries including insertion of an indwelling catheter or sentinel lymph node biopsy within 24 h prior to chemotherapy. Definite contraindications for the use of corticosteroids except inhalative corticoids. Known hypersensitivity reaction to one of the compounds or incorporated substances used in this protocol; Concurrent treatment with:  chronic corticosteroids unless initiated > 6 months prior to study entry and at low dose (≤ 10 mg methylprednisolone or equivalent). sex hormones. Prior treatment must be stopped before study entry. virostatic agents like sorivudine or analogs like brivudine, concurrent treatment with aminoglycosides. anticoagulants: heparin, warfarin as well as acetylic acid (e.g. Aspirin®) at a dose of > 325 mg/day or clopidogrel at a dose of > 75 mg/day. other experimental drugs or any other anti-cancer therapy. drugs recognized as being strong inhibitors or inducers of the isoenzyme CYP3A, e.g. Rifabutin, Rifampicin, Clarithromycin, Ketoconazole, Itraconazole, Ritonavir, Telithromycin, Erythromycin, Verapamil, Diltiazem within the last 5 days or the expected need for these treatments during study participation. Participation in another clinical trial with any investigational, not marketed drug within 30 days prior to study entry. Male patients. |
| Wu 2018 | The inclusion criteria were as follows: (1) patients with operable, previously untreated, clinical stage I to III (T1b-1c, N1-3, M0 or T2-4, N0-3, M0), ER- and PR-negative (defined as ER and PR expression < 10% nuclei staining, according to the 2010 American Society of Clinical Oncology/College of American Pathologists guidelines), HER2-negative (IHC 0-1 + or FISH ratio < 2.0), non- inflammatory invasive breast cancer confirmed by biopsy; (2) patients aged at most 70 years with a Karnofsky score of at least 70 points; (3) patients with adequate hematologic, renal, and hepatic function (defined as an absolute neutrophil count ≥ 1.5 × 10^{9}1^{-1}, a hemoglobin count ≥ 80 g1^{-1},, a platelet count ≥ 75 × 10^{9}1^{-1}, creatinine ≤ 1.5 × the upper limit of the normal range (ULN), total bilirubin ≤ 1.5 × ULN, or alanine aminotransferase or aspartate aminotransferase ≤ 3 × ULN), normal cardiopulmonary and cardiac function as assessed by echocardiography, and no surgical indication or contraindication for chemoradiotherapy; and (4) women with childbearing potential who showed a negative result on a pregnancy test. | Patients with grade 2 or higher neuropathy and patients with contraindications for treatment with platinum, including uncontrolled thrombocytopenia or other malignant tumors, were excluded. |
| Zhang 2016 | Major eligibility criteria included: 1) women aged 18-75 years; 2) ECOG score 0-1; 3) pathologically confirmed breast invasive ductal cancer by core needle biopsy, ER/PR/Her-2 negative by immunohistochemistry IHC) , 4) clinical stage IIA-IIIC with NAC indication; 5) measurable lesions; 6) normal cardiac, hepatic and marrow function. | Patients were excluded if they had a history of invasive cancer or prior exposure to chemotherapy/ radiotherapy. |
| Zhang 2020 | Age >=18 years. Newly diagnosed TNBC of either T1cN1-2 or T2-4N0-2. ECOG PS 0-1. | NR |

## Table S3 Characteristics of Interventions

| **Study ID** | **Intervention** | **Administration of Intervention** | **Treatment other than Intervention** | **Duration (weeks)** | **paclitaxel** |
| --- | --- | --- | --- | --- | --- |
| Alba 2012 | (epirubicin+cyclophosphamide)->docetaxel | 1) segment 1 treatment: epirubicin 90 mg/m2 plus cyclophosphamide 600 mg/m2 once every 21 days for 4 cycles. 2) segment 2 treatment: docetaxel 100 mg/m2 once every 21 days for 4 cycles. | After the neoadjuvant therapy, patients underwent mastectomy or conservative surgery plus axillary lymph node dissection (unless previous negative sentinel lymph node biopsy). Postoperative treatment was left under the investigator’s criteria | 24 | NA |
| Alba 2012 | (epirubicin+cyclophosphamide)->(carboplatin+docetaxel) | 1) segment 1 treatment: epirubicin 90 mg/m2 plus cyclophosphamide 600 mg/m2 once every 21 days for 4 cycles. 2) segment 2 treatment: docetaxel 75 mg/m2 plus carboplatin AUC 6 mg/ml/min once every 21 days for 4 cycles. | After the neoadjuvant therapy, patients underwent mastectomy or conservative surgery plus axillary lymph node dissection (unless previous negative sentinel lymph node biopsy). Postoperative treatment was left under the investigator’s criteria | 24 | NA |
| Ando 2014 | (carboplatin+weekly paclitaxel )->(cyclophosphamide+epirubicin+5-fluorouracil ) (CP–CEF) | 1) segment 1 treatment: four 3-week cycles of CBDCA (carboplatin) [area under the curve, 5 mg/mL/min, day1] and wPTX (weekly paclitaxel) [80 mg/m2, day 1, 8, 15]. 2) segment 2 treatment: four 3-week cycles of CEF (cyclophosphamide, epirubicin, 5-fluorouracil) [500/100/500 mg/ m2]. | 1) If a patient developed grade C3 febrile neutropenia, thrombocytopenia \25,000/mm3, or grade C3 non-hematologic toxicity while receiving CP or CEF, the doses of carboplatin and epirubicin were reduced by 20 and 25 %, respectively, in subsequent cycles. The doses of paclitaxel during CP and P were reduced by 25 % in subsequent cycles if a patient developed grade 3 neurotoxicity. Before administration of the following cycle of CP, P, or CEF, patients were required to have a granulocyte count C1,500/mm3, platelet count C75,000/mm3, and no nonhematologic toxicity of grade B2 (excluding alopecia). Before administration of CP on day 8 and 15, patients were required to have a granulocyte count C500/mm3, platelet count C75,000/mm3, and peripheral neuropathy of grade B2. If toxicity did not improve within 2 weeks on the P or CP regimen, chemotherapy was discontinued and initiation of CEF was recommended. If toxicity did not improve within 2 weeks on CEF, chemotherapy was discontinued and surgery was recommended. 2) Patients who were considered candidates for breast-conserving therapy (BCT) were offered lumpectomy. Axillary lymph node dissection (AxLND) was mandatory, except in patients diagnosed as having no metastases by SLNB before neoadjuvant chemotherapy. Surgery was performed within 8 weeks after completion of preoperative chemotherapy. All patients who underwent BCT received wholebreast irradiation. | 24 | weekly |
| Ando 2014 | weekly paclitaxel->(cyclophosphamide+epirubicin+5-fluorouraci l) (P–CEF) | 1) segment 1 treatment: four cycles of wPTX (weekly paclitaxel) every 3 weeks 2) segment 2 treatment: four cycles of CEF (cyclophosphamide, epirubicin, 5-fluorouracil) [500/100/500 mg/m2] every 3 weeks. | 1) If a patient developed grade C3 febrile neutropenia, thrombocytopenia \25,000/mm3, or grade C3 non-hematologic toxicity while receiving CP or CEF, the doses of carboplatin and epirubicin were reduced by 20 and 25 %, respectively, in subsequent cycles. The doses of paclitaxel during CP and P were reduced by 25 % in subsequent cycles if a patient developed grade 3 neurotoxicity. Before administration of the following cycle of CP, P, or CEF, patients were required to have a granulocyte count C1,500/mm3, platelet count C75,000/mm3, and no nonhematologic toxicity of grade B2 (excluding alopecia). Before administration of CP on day 8 and 15, patients were required to have a granulocyte count C500/mm3, platelet count C75,000/mm3, and peripheral neuropathy of grade B2. If toxicity did not improve within 2 weeks on the P or CP regimen, chemotherapy was discontinued and initiation of CEF was recommended. If toxicity did not improve within 2 weeks on CEF, chemotherapy was discontinued and surgery was recommended. 2) Patients who were considered candidates for breast-conserving therapy (BCT) were offered lumpectomy. Axillary lymph node dissection (AxLND) was mandatory, except in patients diagnosed as having no metastases by SLNB before neoadjuvant chemotherapy. Surgery was performed within 8 weeks after completion of preoperative chemotherapy. All patients who underwent BCT received wholebreast irradiation. | 24 | weekly |
| Bear 2012 | bevacizumab+[docetaxel or docetaxel+capecitabine or docetaxel+gemcitabine)->(cyclophosphamide+doxorubicin)] | bevacizumab (15 mg/kg) added to first 6 cycles of chemotherapy, definied as following: 1) segment 1 treatment: 4 cycles of docetaxel (100 mg/m2, IV) once every 3 weeks. OR capecitabine (825 mg/m2 twice a day on days 1-14, orally) plus 4 cycles of docetaxel (75 mg/m2, IV) once every 3 weeks. OR gemcitabine (1000 mg/m2 on day 1 and day 8, IV) plus 4 cycles of docetaxel (75 mg/m2, IV) once every 3 weeks. 2) segment 2 treatment: 4 cycles of doxorubicin-cyclophosphamide (60 and 600 mg/m2 respectively, IV) once every 3 weeks. | For patients receiving bevacizumab who underwent all four cycles of doxorubicin– cyclophosphamide, surgery was performed at least 9 weeks after the last dose of bevacizumab. If chemotherapy was stopped before completion of the planned therapy, surgery was performed at least 4 weeks, and preferably 6 weeks, after the last dose of bevacizumab. The type of surgery that was performed was left to the discretion of the patient and surgeon. For patients undergoing breast reconstruction, tissue expansion could not be performed within the 2 weeks before the first postoperative dose of bevacizumab. Expansion or any surgical procedure (e.g., exchanging tissue expanders for permanent implants) was prohibited throughout the course of bevacizumab therapy an a minimum period of 6 weeks after the last dose of bevacizumab. | 24 | NA |
| Bear 2012 | docetaxel or docetaxel+capecitabine or docetaxel+gemcitabine)->(cyclophosphamide+doxorubicin) | 1) segment 1 treatment: 4 cycles of docetaxel (100 mg/m2, IV) once every 3 weeks. OR capecitabine (825 mg/m2 twice a day on days 1-14, orally) plus 4 cycles of docetaxel (75 mg/m2, IV) once every 3 weeks. OR gemcitabine (1000 mg/m2 on day 1 and day 8, IV) plus 4 cycles of docetaxel (75 mg/m2, IV) once every 3 weeks. 2) segment 2 treatment: 4 cycles of doxorubicin-cyclophosphamide (60 and 600 mg/m2 respectively, IV) once every 3 weeks. | For patients receiving bevacizumab who underwent all four cycles of doxorubicin– cyclophosphamide, surgery was performed at least 9 weeks after the last dose of bevacizumab. If chemotherapy was stopped before completion of the planned therapy, surgery was performed at least 4 weeks, and preferably 6 weeks, after the last dose of bevacizumab. The type of surgery that was performed was left to the discretion of the patient and surgeon. For patients undergoing breast reconstruction, tissue expansion could not be performed within the 2 weeks before the first postoperative dose of bevacizumab. Expansion or any surgical procedure (e.g., exchanging tissue expanders for permanent implants) was prohibited throughout the course of bevacizumab therapy an a minimum period of 6 weeks after the last dose of bevacizumab. | 24 | NA |
| Chen 2016 | docetaxel+cyclophosphamide | 6 cycles of docetaxel 75 mg/m2 plus cyclophosphamide 600 mg/m2 (TC) on day 1 every 21 days | In both arms, docetaxel was given with routine dexamethasone 8 mg orally twice per day premedication for 3 days, starting the day before docetaxel administration. Primary prophylaxis with granulocyte colony-stimulating factor (G-CSF) from the first cycle was required in TEC arm and was recommended for secondary prophylaxis after an episode of febrile neutropenia or grade 4 neutropenia. In TC arm, prophylaxis G-CSF was also allowed to be used between cycles by investigator’s discretion. Dose reduction was required for severe hematologic and/or non-hematologic toxicities, and dose re-escalation in subsequent cycles was not allowed except for transient transaminase elevations. Patients with operable disease after chemotherapy should receive surgery within 6 weeks after the last scheduled chemotherapy cycle. Additional adjuvant chemotherapy was not allowed. All patients were required to receive adjuvant radiotherapy. | 18 | NA |
| Chen 2016 | docetaxel+anthracycline+cyclophosphamide | 6 cycles of docetaxel 75 mg/m2, anthracycline, and cyclophosphamide 500 mg/m2 (TAC) on day 1 every 21 days | In both arms, docetaxel was given with routine dexamethasone 8 mg orally twice per day premedication for 3 days, starting the day before docetaxel administration. Primary prophylaxis with granulocyte colony-stimulating factor (G-CSF) from the first cycle was required in TEC arm and was recommended for secondary prophylaxis after an episode of febrile neutropenia or grade 4 neutropenia. In TC arm, prophylaxis G-CSF was also allowed to be used between cycles by investigator’s discretion. Dose reduction was required for severe hematologic and/or non-hematologic toxicities, and dose re-escalation in subsequent cycles was not allowed except for transient transaminase elevations. Patients with operable disease after chemotherapy should receive surgery within 6 weeks after the last scheduled chemotherapy cycle. Additional adjuvant chemotherapy was not allowed. All patients were required to receive adjuvant radiotherapy. | 18 | NA |
| Earl 2015 | docetaxel->(fluorouracil+epirubicin+cyclophosphamide) | 1) segment 1 treatment: three cycles of docetaxel (100 mg/m2 once every 21 days) 2) segment 2 treatment: three cycles of fluorouracil (500 mg/m2), epirubicin (100 mg/m2), and cyclophosphamide (500 mg/m2) once every 21 days | Surgery (breast and axillary), radiotherapy, and adjuvant endocrine treatment were done according to local protocols. | 18 | NA |
| Earl 2015 | bevacizumab+[docetaxel->(fluorouracil+epirubicin+cyclophosphamide)] | 1) segment 1 treatment: three cycles of docetaxel (100 mg/m2) plus bevacizumab (15mg/kg) once every 21 days.  2) segment 2 treatment: one cycle of bevacizumab (15 mg/kg) once every 21 days plus three cycles of fluorouracil (500 mg/m2), epirubicin (100 mg/m2), and cyclophosphamide (500 mg/m2) once every 21 days | Surgery (breast and axillary), radiotherapy, and adjuvant endocrine treatment were done according to local protocols. | 18 | NA |
| Fasching 2019 | weekly paclitaxel+olaparib->(epirubicin+cyclophosphamide) | 1) segment 1 treatment: 12 x Paclitaxel weekly 80mg/m+Olaparib tablets 100mg twice daily (PO). 2) segment 2 treatment: 4 cycles of epirubicin 90 mg/m² and cyclophosphamide 600 mg/m² (EC) either every 3 or every 2 weeks. | Procedure: Surgery after neoadjuvant Therapy In both study arms, treatment will be given until surgery, disease progression, unacceptable toxicity, or withdrawal of consent of the patients. | 20-24 | weekly |
| Fasching 2019 | (weekly paclitaxel+carboplatin)->(epirubicin+cyclophosphamide) | 1) segment 1 treatment: 12x Paclitaxel weekly 80mg/m2+Carboplatin AUC 2 (PCb). 2) segment 2 treatment: 4 cycles of epirubicin 90 mg/m² and cyclophosphamide 600 mg/m² (EC) either every 3 or every 2 weeks. | Procedure: Surgery after neoadjuvant Therapy In both study arms, treatment will be given until surgery, disease progression, unacceptable toxicity, or withdrawal of consent of the patients. | 20-24 | weekly |
| Gerber 2013 | [(epirubicin+cyclophosphamide)->docetaxel]+ bevacizumab | 1) segment 1 treatment: epirubicin (E, 90 mg/m2) plus cyclophosphamide (C, 600 mg/m2), both administered on day 1, every 3 weeks for four cycles. And receive four cycles of bevacizumab (B, 15 mg/kg body weight) intravenously every 3 weeks starting on day 1 of the first EC cycle.  2) segment 2 treatment: followed by four cycles of docetaxel (D, 100 mg/m2) on day 1, every 3 weeks. And receive four cycles of bevacizumab (B, 15 mg/kg body weight) intravenously every 3 weeks starting on day 1 of the first docetaxel cycle. | In cases of tumor progression, the study treatment was discontinued and further local or systemic treatment was permitted at the investigator’s discretion. Patients could undergo surgery at least 28 days after the last chemotherapy ± bevacizumab. | 24 | NA |
| Gerber 2013 | (epirubicin+cyclophosphamide)->docetaxel | 1) segment 1 treatment: epirubicin (E, 90 mg/m2) plus cyclophosphamide (C, 600 mg/m2), both administered on day 1, every 3 weeks for four cycles.  2) segment 2 treatment: followed by four cycles of docetaxel (D, 100 mg/m2) on day 1, every 3 weeks. | In cases of tumor progression, the study treatment was discontinued and further local or systemic treatment was permitted at the investigator’s discretion. Patients could undergo surgery at least 28 days after the last chemotherapy ± bevacizumab. | 24 | NA |
| Geyer 2017 | (weekly paclitaxel+veliparib+carboplatin)->(doxorubicin+cyclophosphamide) | 1) segment 1 treatment: paclitaxel 80 mg/m2 weekly + carboplatin area under curve 6 mg/mL/min once every 3 weeks + veliparib 50 mg PO BID, for 12 weeks. 2) segment 2 treatment: doxorubicin + cyclophosphamide (60 mg/m2or 600 mg/m2q2 or 3 weeks) × 4. | None | 20-24 | weekly |
| Geyer 2017 | (weekly paclitaxel+carboplatin)->(doxorubicin+cyclophosphamide) | 1) segment 1 treatment: paclitaxel 80 mg/m2 weekly + carboplatin area under curve 6 mg/mL/min once every 3 weeks. 2) segment 2 treatment: doxorubicin + cyclophosphamide (60 mg/m2or 600 mg/m2q2 or 3 weeks) × 4. | None | 20-24 | weekly |
| Gianni 2018 | weekly paclitaxel->(doxorubicin+cyclophosphamide) or (epirubicin+cyclophosphamide) or (fluorouracil+epirubicin+cyclophosphamide) | 1) segment 1 treatment: paclitaxel (90 mg/m²) intravenously on weeks 1, 2, and 3, followed by a 1-week rest, for 4 cycles. 2) segment 2 treatment: AC or EC (adriamycin or epirubicin and cyclophosphamide) on day 1 every 3 weeks for 4 cycles or FEC (fluorouracil, epirubicin, and cyclophosphamide) on day 1 every three weeks for 4 cycles. | In the presence of severe toxic effects, investigators decided whether to discontinue neoadjuvant therapy and perform surgery immediately. After neoadjuvant chemotherapy and surgery, patients with hormone receptor–positive tumors had to receive endocrine therapy according to local guidelines. Postsurgery irradiation was recommended in accordance with international and local guidelines | 28 | weekly |
| Gianni 2018 | weekly Noneb-paclitaxel->(doxorubicin+cyclophosphamide) or (epirubicin+cyclophosphamide) or (fluorouracil+epirubicin+cyclophosphamide) | 1) segment 1 treatment: Noneb-paclitaxel (125mg/m²), intravenously on weeks 1, 2, and 3, followed by a 1-week rest, for 4 cycles. 2) segment 2 treatment: AC or EC (adriamycin or epirubicin and cyclophosphamide) on day 1 every 3 weeks for 4 cycles or FEC (fluorouracil, epirubicin, and cyclophosphamide) on day 1 every three weeks for 4 cycles. | In the presence of severe toxic effects, investigators decided whether to discontinue neoadjuvant therapy and perform surgery immediately. After neoadjuvant chemotherapy and surgery, patients with hormone receptor–positive tumors had to receive endocrine therapy according to local guidelines. Postsurgery irradiation was recommended in accordance with international and local guidelines | 28 | weekly |
| Gigolaeva 2019 | (doxorubicin+cyclophosphamide)->weekly paclitaxel | 1) segment 1 treatment: doxorubicin (60 mg/m2) plus cyclophosphamide (600 mg/m2), every 3 weeks, for 4 cycles. 2) segment 2 treatment: paclitaxel (80 mg/m2), weekly for 12 cycles. | None | 24 | weekly |
| Gigolaeva 2019 | (doxorubicin+cyclophosphamide)->(carboplatin+q3w paclitaxel) or (carboplatin+eribulin) | 1) segment 1 treatment: doxorubicin (60 mg/m2) plus cyclophosphamide (600 mg/m2), every 3 weeks, for 4 cycles. 2) segment 2 treatment: carboplatin (AUC2)×12+eribulin (1,4 mg/m2) (30 patients) or carboplatin AUC2×12 + paclitaxel 175 mg/m2 (32 patients) every 3 weeks | None | 24 | 3-weekly |
| Gluz 2018 | gemcitabine+q3w nab-paclitaxel | nab-paclitaxel (Abraxane, Celgene Corporation, Summit, NJ) 125 mg/m2 d1, 8 for four three-week cycles combined with gemcitabine 1000 mg/m2 d1, 8. | None | 12 | 3-weekly |
| Gluz 2018 | carboplatin+q3w Noneb-paclitaxel | Noneb-paclitaxel (Abraxane, Celgene Corporation, Summit, NJ) 125 mg/m2 d1, 8 for four three-week cycles combined with carboplatin (area under curve [AUC]= 2) d1, 8. | None | 12 | 3-weekly |
| Gonzalez-Angulo 2014 | weekly paclitaxe->(5-fluorouracil+epirubicin+cyclophosphamide)（P-FEC） | 1) segment 1 treatment: paclitaxel (80 mg/m², intravenously, weekly, for 12 weeks).  2) segment 2 treatment: 5-fluorouracil (500 mg/m²) plus epirubicin (100 mg/m²) plus cyclophosphamide 500 mg/m², every 3 weeks for four cycles. | All patients underwent surgery and adjuvant radiotherapy if indicated. Chemotherapy dose reductions were permitted as per protocol. Everolimus was interrupted for grade 2 nonhematological toxicity or grade 2 thrombocytopenia and reintroduced at the initial dose after recovery to grade ≤1. If grade 2 pneumonitis, grade 3 nonhematologic toxicity, thrombocytopenia, or neutropenia occurred, treatment was interrupted and then resumed at 15 mg p.o. weekly after recovery to grade <1. Treatment was discontinued for any grade 4 toxicity, if treatment was interrupted for >21 days, or if toxicity reoccurred after dose reduction. | 24 | weekly |
| Gonzalez-Angulo 2014 | (everolimus+weekly paclitaxe)->(5-fluorouracil+epirubicin+cyclophosphamide)（PR-FEC ） | 1) segment 1 treatment: paclitaxel (80 mg/m², intravenously) plus everolimus (30 mg orally), weekly for 12 weeks. 2) segment 2 treatment: 5-fluorouracil (500 mg/m²) plus epirubicin (100 mg/m²) plus cyclophosphamide 500 mg/m², every 3 weeks for four cycles. | All patients underwent surgery and adjuvant radiotherapy if indicated. Chemotherapy dose reductions were permitted as per protocol. Everolimus was interrupted for grade 2 nonhematological toxicity or grade 2 thrombocytopenia and reintroduced at the initial dose after recovery to grade ≤1. If grade 2 pneumonitis, grade 3 nonhematologic toxicity, thrombocytopenia, or neutropenia occurred, treatment was interrupted and then resumed at 15 mg p.o. weekly after recovery to grade <1. Treatment was discontinued for any grade 4 toxicity, if treatment was interrupted for >21 days, or if toxicity reoccurred after dose reduction. | 24 | weekly |
| Harbeck 2020 | (atezolizumab+weekly nab-paclitaxel)->(cyclophosphamide+doxorubicin) | 1) segment 1 treatment: atezolizumab 840 mg IV q2w + nab-paclitaxel 125 mg/m2 IV qw. 2) segment 2 treatment: atezolizumab 840 mg IV q2w + doxorubicin 60 mg/m2 IV q2w plus cyclophosphamide 600 mg/m2 IV q2w | Drug: Filgrastim Filgrastim will be administered according to local prescribing information as determined by the Investigator for 4 doses after completion of initial 12 weeks. Drug: Pegfilgrastim Pegfilgrastim will be administered according to local prescribing information as determined by the Investigator for 4 doses after completion of initial 12 weeks. Participants will continue to receive unblinded atezolizumab post-surgery at a fixed dose of 1200 mg by IV infusion every 3 weeks for 11 doses, for a total of approximately 12 months of atezolizumab therapy. | 20 | weekly |
| Harbeck 2020 | weekly Noneb-paclitaxel->(cyclophosphamide+doxorubicin) | 1) segment 1 treatment: Noneb-paclitaxel 125 mg/m2 IV qw. 2)segment 2 treatment: atezolizumab 840 mg IV q2w + doxorubicin 60 mg/m2 IV q2w plus cyclophosphamide 600 mg/m2 IV q2w | Drug: Filgrastim Filgrastim will be administered according to local prescribing information as determined by the Investigator for 4 doses after completion of initial 12 weeks. Drug: Pegfilgrastim Pegfilgrastim will be administered according to local prescribing information as determined by the Investigator for 4 doses after completion of initial 12 weeks. Participants will be unblinded post-surgery and will continue to be followed. | 20 | weekly |
| Ishikawa 2016 | docetaxel+cyclophosphamide | Patients received either TC6 or FEC-D every 3 weeks. docetaxel (75 mg/m²) plus cyclophosphamide (600 mg/m²), for 6 cycles. | None | 18 | NA |
| Ishikawa 2016 | (5-fluorouracil+epirubicin+cyclophosphamide)->docetaxel | 1) segment 1 treatment: 5-fluorouracil (500 mg/m²) plus epirubicin (100mg/m²) plus cyclophosphamide (500mg/m²), for three cycles. 2) segment 2 treatment: docetaxel (100 mg/m2), intravously, on day 1 of each cycle, for three cycles.  Patients received either TC6 or FEC-D every 3 weeks. | None | 18 | NA |
| Jovanovic 2017 | weekly paclitaxel+cisplatin+everolimus | cisplatin (25 mg/m², intravenously, weekly, for 12 weeks) plus everolimus (5 mg, orally, daily, for 12 weeks) plus paclitaxel (80 mg/m², intravenously, weekly, for 11 weeks, starting 1 week after cisplatin initiation) | Definitive surgery was scheduled 3–6 weeks after treatment completion. Postoperative adjuvant treatment was offered at the discretion of the treating team (not part of protocol procedures), but it is of note that over 90% of all patients in the trial (in both arms), regardless of pathologic response, received an anthracycline-containing regimen postoperatively | 12 | weekly |
| Jovanovic 2017 | weekly paclitaxel+cisplatin | cisplatin (25 mg/m², intravenously, weekly, for 12 weeks) plus placebo (orally, daily, for 12 weeks) plus paclitaxel (80 mg/m², intravenously, weekly, for 11 weeks, starting 1 week after cisplatin initiation) | Definitive surgery was scheduled 3–6 weeks after treatment completion. Postoperative adjuvant treatment was offered at the discretion of the treating team (not part of protocol procedures), but it is of note that over 90% of all patients in the trial (in both arms), regardless of pathologic response, received an anthracycline-containing regimen postoperatively | 12 | weekly |
| Kummel 2017 | cabazitaxel | cabazitaxel (25 mg/m² on day 1q 3 weeks for a total of four cycles) | Followed by surgery and adjuvant epirubicin and cyclophosphamide (EC) as per investigator discretion | 12 | NA |
| Kummel 2017 | weekly paclitaxel | paclitaxel (80mg/m², weekly, for 12 weeks) | Followed by surgery and adjuvant epirubicin and cyclophosphamide (EC) as per investigator discretion | 12 | weekly |
| Llombart-Cussac 2015 | weekly paclitaxel | paclitaxel (80 mg/m2, d1, weekly, for 12 weeks) | None | 12 | weekly |
| Llombart-Cussac 2015 | weekly paclitaxel+one-weekly iniparib | paclitaxel (80 mg/m2, d1, weekly) plus iniparib (11.2 mg/kg, d1 on a once-weekly schedule), for 12 weeks | None | 12 | weekly |
| Llombart-Cussac 2015 | weekly paclitaxel+twice-weekly iniparib | paclitaxel (80 mg/m2, d1, weekly) plus iniparib (5.6 mg/kg, d1, 4, twice-weekly), for 12 weeks | None | 12 | weekly |
| Loi 2019 | [weekly nab-paclitaxel->(cyclophosphamide+doxorubicin)]+pembrolizumab | 1) segment 1 treatment: pembrolizumab (200 mg, intravenously, day 1, every 3 weeks, in cycle 1-9) plus nab-paclitaxel (125 mg/m², days 1, 8, and 15, weekly, in cycle 2-5)  2) segment 2 treatment: doxorubicin (60 mg/m², intravenously, day 1, every 3 weeks, in cycle 6-9) plus cyclophosphamide (600 mg/m², intravenously, day 1, every 3 weeks, in cycle 6-9) | Patients received a standard dose of 200 mg pembrolizumab every 3 weeks for a total of nine doses before surgery or until unacceptable toxicity or withdrawal of consent. Dose modifications for pembrolizumab were not allowed; however, interruptions for up to 6 weeks were permitted in case of adverse events, as judged by the investigator. Chemotherapy dose modifications followed the label inserts and/or institutional or local guidelines. Specific guidance was given for options after discontinuation of one or more chemotherapeutic agents, as described in the Supplementary Information, available at Annals of Oncology online. | 27 | weekly |
| Loi 2019 | [(carboplatin (AUC 6)+weekly Noneb-paclitaxel (100 mg/m²))->(cyclophosphamide+doxorubicin)]+pembrolizumab | 1) segment 1 treatment: pembrolizumab (200 mg, intravenously, day 1, every 3 weeks, in cycle 1-9) plus Noneb-paclitaxel 100 mg/m², intravenously, days 1, 8, and 15, weekly, in cycle 2-5) plus carboplatin (AUC 6, day 1, every 3 weeks, in ycle 2-5) 2) segment 2 treatment: doxorubicin (60 mg/m², intravenously, day 1, every 3 weeks, in cycle 6-9) plus cyclophosphamide (600 mg/m², intravenously, day 1, every 3 weeks, in cycle 6-9) | Patients received a standard dose of 200 mg pembrolizumab every 3 weeks for a total of nine doses before surgery or until unacceptable toxicity or withdrawal of consent. Dose modifications for pembrolizumab were not allowed; however, interruptions for up to 6 weeks were permitted in case of adverse events, as judged by the investigator. Chemotherapy dose modifications followed the label inserts and/or institutional or local guidelines. Specific guidance was given for options after discontinuation of one or more chemotherapeutic agents, as described in the Supplementary Information, available at Annals of Oncology online. | 27 | weekly |
| Loi 2019 | [(carboplatin (AUC 5) +weekly nab-paclitaxel (125 mg/m²))->(cyclophosphamide+doxorubicin)]+pembrolizumab | 1) segment 1 treatment: pembrolizumab (200 mg, intravenously, day 1, every 3 weeks, in cycle 1-9) plus Nab-paclitaxel 125 mg/m², intravenously, days 1, 8, and 15, weekly, in cycle 2-5) plus carboplatin (AUC 5, day 1, every 3 weeks, in ycle 2-5) 2) segment 2 treatment: doxorubicin (60 mg/m², intravenously, day 1, every 3 weeks, in cycle 6-9) plus cyclophosphamide (600 mg/m², intravenously, day 1, every 3 weeks, in cycle 6-9) | Patients received a standard dose of 200 mg pembrolizumab every 3 weeks for a total of nine doses before surgery or until unacceptable toxicity or withdrawal of consent. Dose modifications for pembrolizumab were not allowed; however, interruptions for up to 6 weeks were permitted in case of adverse events, as judged by the investigator. Chemotherapy dose modifications followed the label inserts and/or institutional or local guidelines. Specific guidance was given for options after discontinuation of one or more chemotherapeutic agents, as described in the Supplementary Information, available at Annals of Oncology online. | 27 | weekly |
| Loi 2019 | [(carboplatin (AUC 2)+weekly nab-paclitaxel (125 mg/m²))-> (cyclophosphamide+doxorubicin)]+pembrolizumab | 1) segment 1 treatment: pembrolizumab (200 mg, intravenously, day 1, every 3 weeks, in cycle 1-9) plus Nab-paclitaxel 125 mg/m², intravenously, days 1, 8, and 15, weekly, in cycle 2-5) plus carboplatin (AUC 2 days 1, 8, and 15, weekly, in cycle 2-5) 2) segment 2 treatment: doxorubicin (60 mg/m², intravenously, day 1, every 3 weeks, in cycle 6-9) plus cyclophosphamide (600 mg/m², intravenously, day 1, every 3 weeks, in cycle 6-9) | Patients received a standard dose of 200 mg pembrolizumab every 3 weeks for a total of nine doses before surgery or until unacceptable toxicity or withdrawal of consent. Dose modifications for pembrolizumab were not allowed; however, interruptions for up to 6 weeks were permitted in case of adverse events, as judged by the investigator. Chemotherapy dose modifications followed the label inserts and/or institutional or local guidelines. Specific guidance was given for options after discontinuation of one or more chemotherapeutic agents, as described in the Supplementary Information, available at Annals of Oncology online. | 27 | weekly |
| Loi 2019 | [(carboplatin ( (AUC 5)+weekly paclitaxel (80 mg/m²))->(cyclophosphamide+doxorubicin)]+pembrolizumab | 1) segment 1 treatment: pembrolizumab (200 mg, intravenously, day 1, every 3 weeks, in cycle 1-9) plus paclitaxel 80 mg/m², intravenously, days 1, 8, and 15, weekly, in cycle 2-5) plus carboplatin (AUC 5, day 1, every 3 weeks, in cycle 2-5) 2) segment 2 treatment: doxorubicin (60 mg/m², intravenously, day 1, every 3 weeks, in cycle 6-9) plus cyclophosphamide (600 mg/m², intravenously, day 1, every 3 weeks, in cycle 6-9) | Patients received a standard dose of 200 mg pembrolizumab every 3 weeks for a total of nine doses before surgery or until unacceptable toxicity or withdrawal of consent. Dose modifications for pembrolizumab were not allowed; however, interruptions for up to 6 weeks were permitted in case of adverse events, as judged by the investigator. Chemotherapy dose modifications followed the label inserts and/or institutional or local guidelines. Specific guidance was given for options after discontinuation of one or more chemotherapeutic agents, as described in the Supplementary Information, available at Annals of Oncology online. | 27 | weekly |
| Loi 2019 | [(carboplatin (AUC 2)+weekly paclitaxel (80 mg/m²))->(cyclophosphamide+doxorubicin)]+pembrolizumab | 1) segment 1 treatment: pembrolizumab (200 mg, intravenously, day 1, every 3 weeks, in cycle 1-9) plus paclitaxel 80 mg/m², intravenously, days 1, 8, and 15, weekly, in cycle 2-5) plus carboplatin (AUC 2 days 1, 8, and 15, weekly, in cycle 2-5) 2) segment 2 treatment: doxorubicin (60 mg/m², intravenously, day 1, every 3 weeks, in cycle 6-9) plus cyclophosphamide (600 mg/m², intravenously, day 1, every 3 weeks, in cycle 6-9) | Patients received a standard dose of 200 mg pembrolizumab every 3 weeks for a total of nine doses before surgery or until unacceptable toxicity or withdrawal of consent. Dose modifications for pembrolizumab were not allowed; however, interruptions for up to 6 weeks were permitted in case of adverse events, as judged by the investigator. Chemotherapy dose modifications followed the label inserts and/or institutional or local guidelines. Specific guidance was given for options after discontinuation of one or more chemotherapeutic agents, as described in the Supplementary Information, available at Annals of Oncology online. | 27 | weekly |
| Loibl 2018 | carboplatin+weekly paclitaxel+veliparib->doxorubicin+cyclophosphamide | 1) segment 1 treatment: paclitaxel (80 mg/m² intravenously weekly for 12 doses) plus carboplatin (area under the curve [AUC] 6 mg/mL per min, intravenously every 3 weeks, for four cycles) plus veliparib (50 mg orally twice a day). 2) segment 2 treatment: doxorubicin (60 mg/m²) and cyclophosphamide (600 mg/m²) every 2 or 3 weeks for four cycles to complete the neoadjuvant regimen. | Administration of granulocyte colony-stimulating factor (G-CSF), locally sourced by investigators according to institutional practices, was recommended according to guidelines depending on the schedule of doxorubicin and cyclophosphamide. G-CSF generic formulations manufactured in multiple locations globally were used. Patients stopped treatment if they had clinical disease progression, required alternative anti-cancer agents, surgery, or radiotherapy for primary or metastatic disease, became pregnant, or for other medical reasons at the investigators’ discretion. | 20-24 | weekly |
| Loibl 2018 | carboplatin+weekly paclitaxel->doxorubicin+cyclophosphamide | 1) segment 1 treatment: paclitaxel (80 mg/m² weekly for 12 doses) plus carboplatin (AUC 6 mg/mL per min, every 3 weeks, for four cycles) plus veliparib placebo (capsules matching 50 mg veliparib capsules, twice a day). 2) segment 2 treatment: doxorubicin (60 mg/m²) and cyclophosphamide (600 mg/m²) every 2 or 3 weeks for four cycles to complete the neoadjuvant regimen. | Administration of granulocyte colony-stimulating factor (G-CSF), locally sourced by investigators according to institutional practices, was recommended according to guidelines depending on the schedule of doxorubicin and cyclophosphamide. G-CSF generic formulations manufactured in multiple locations globally were used. Patients stopped treatment if they had clinical disease progression, required alternative anti-cancer agents, surgery, or radiotherapy for primary or metastatic disease, became pregnant, or for other medical reasons at the investigators’ discretion. | 20-24 | weekly |
| Loibl 2018 | weekly paclitaxel->doxorubicin+cyclophosphamide | 1）segment 1 treatment: paclitaxel (80 mg/m² weekly for 12 doses) plus carboplatin placebo (0·9% sodium chloride injection every 3 weeks for four cycles) plus veliparib placebo (capsules matching 50 mg veliparib capsules, twice a day). 2) segment 2 treatment: doxorubicin (60 mg/m²) and cyclophosphamide (600 mg/m²) every 2 or 3 weeks for four cycles to complete the neoadjuvant regimen. | Administration of granulocyte colony-stimulating factor (G-CSF), locally sourced by investigators according to institutional practices, was recommended according to guidelines depending on the schedule of doxorubicin and cyclophosphamide. G-CSF generic formulations manufactured in multiple locations globally were used. Patients stopped treatment if they had clinical disease progression, required alternative anti-cancer agents, surgery, or radiotherapy for primary or metastatic disease, became pregnant, or for other medical reasons at the investigators’ discretion. | 20-24 | weekly |
| Loibl 2019 | durvalumab->[weekly nab-paclitaxel->(epirubicin+cyclophosphamide)]+durvalumab | 1) segment 1 treatment: patients received one injection durvalumab 0.75 g i.v. monotherapy 2 weeks before start of chemotherapy (window-phase). 2) segment 2 treatment: followed by durvalumab 1.5 g i.v. every 4 weeks (q4 wks) plus nab-paclitaxel 125 mg/m2 weekly for 12 weeks.  3) segment 3 treatment: followed by durvalumab 1.5 g i.v./placebo q4 wks plus epirubicin/cyclophosphamide q2 wks for 4 cycles. | None | 22 | weekly |
| Loibl 2019 | weekly Noneb-paclitaxel->(epirubicin+cyclophosphamide) | 1) segment 1 treatment: patients received one injection durvalumab placebo 0.75 g i.v. monotherapy 2 weeks before start of chemotherapy (window-phase). 2) segment 2 treatment: followed by durvalumab placebo 1.5 g i.v. every 4 weeks (q4 wks) plus Noneb-paclitaxel 125 mg/m2 weekly for 12 weeks.  3) segment 3 treatment: followed by durvalumab placebo 1.5 g i.v./placebo q4 wks plus epirubicin/cyclophosphamide q2 wks for 4 cycles. | None | 22 | weekly |
| Martinez 2015 | weekly paclitaxel->(5FU+doxorubicin+cyclophosphamide) | 1) segment 1 treatment: weekly paclitaxel 80 mg/m2 by 12. 2) segment 2 treatment: 5FU, doxorubicin and cyclophosphamide 500/50/500 mg/m2 every 3 weeks times 4. | None | 24 | weekly |
| Martinez 2015 | cisplatin+(weekly paclitaxel->doxorubicin) | 1) segment 1 treatment: cisplatin 30 mg/m2 plus weekly paclitaxel 80 mg/m2 by 12. 2) segment 2 treatment: cisplatin 75 mg/m2 plus doxorubicin 50 mg/m2 every 3 weeks for 4 cycles. | None | 24 | weekly |
| Mayer 2019 | cisplatin | cisplatin 75 mg/m2 once every 3 weeks for 4 cycles. | Non-responders at 12 wks could crossover to alternative CT. In response-evaluable pts, 87 (63.0%) had surgery at 12 wks, and 51 (37.0%) crossed over. | 12 | NA |
| Mayer 2019 | weekly paclitaxel | paclitaxel 80 mg/m2 weekly for 12 weeks. | Non-responders at 12 wks could crossover to alterNonetive CT. In response-evaluable pts, 87 (63.0%) had surgery at 12 wks, and 51 (37.0%) crossed over. | 12 | weekly |
| Nahleh 2016 | bevacizumab+weekly nab-paclitaxel-> (doxorubicin+cyclophosphamide) (Bev+nabp-ddAC) | 1) segment 1 treatment: intravenous (IV) administration of nab-paclitaxel 100 mg/m2 IV weekly for 12 weeks with IV bevacizumab 10 mg/kg every 2 weeks (six doses). 2) segment 2 treatment: doxorubicin 60 mg/m2 and cyclophosphamide 600 mg/m2 with pegfilgrastim 6 mg subcutaneously every 2 weeks for six cycles. | SURGERY performed 3-6 weeks after completion of neoadjuvant chemotherapy. | 24 | weekly |
| Nahleh 2016 | weekly Noneb-paclitaxel->( doxorubicin+cyclophosphamide)（Nonebp-ddAC ) (doxorubicin+cyclophosphamide)->weekly Noneb-paclitaxel（ddAC-Nonebp ） | 1) segment 1 treatment: intravenous (IV) administration of Noneb-paclitaxel 100 mg/m2 IV weekly for 12 weeks. 2) segment 2 treatment: IV doxorubicin 60 mg/m2 and cyclophosphamide 600 mg/m2 with pegfilgrastim 6 mg subcutaneously every 2 weeks for six cycles. OR 1) segment 1 treatment: IV doxorubicin 60 mg/m2 and cyclophosphamide 600 mg/m2 with pegfilgrastim 6 mg subcutaneously every 2 weeks for six cycles. 2) segment 2 treatment: IV administration of Noneb-paclitaxel 100 mg/m2 IV weekly for 12 weeks. | SURGERY performed 3-6 weeks after completion of neoadjuvant chemotherapy. | 24 | weekly |
| Nanda 2020 | weekly paclitaxel->(doxorubicin+cyclophosphamide) | 1) segment 1 treatment: 80 mg/m2 intravenous paclitaxel weekly for 12 weeks, 1) segment 2 treatment: 4 cycles of 60 mg/m2 doxorubicin plus 600 mg/m2 intravenous cyclophosphamide every 2 to 3 weeks. | None | 20-24 | weekly |
| Nanda 2020 | [weekly paclitaxel->(doxorubicin+cyclophosphamide)]+pembrolizumab | 1) segment 1 treatment: 80 mg/m2 intravenous paclitaxel weekly for 12 weeks, And 200 mg intravenous pembrolizumab every 3 weeks for 4 cycles (weeks 1, 4, 7, and 10) concurrently with paclitaxel. 1) segment 2 treatment: 4 cycles of 60 mg/m2 doxorubicin plus 600 mg/m2 intravenous cyclophosphamide every 2 to 3 weeks. | None | 20-24 | weekly |
| Rugo 2016 | weekly paclitaxel->(doxorubicin+cyclophosphamide) | 1) segment 1 treatment: weekly paclitaxel at a dose of 80 mg per square meter of body-surface area intra venously for 12 doses. 2) segment 2 treatment: doxorubicin (60 mg per square meter) and cyclophosphamide (600 mg per square meter) intravenously every 2 to 3 weeks for four doses. | None | 20-24 | weekly |
| Rugo 2016 | carboplatin+veliparib+weekly paclitaxel->(doxorubicin+cyclophosphamide) | 1) segment 1 treatment: weekly paclitaxel at a dose of 80 mg per square meter of body-surface area intra venously for 12 doses. And received 50 mg of veliparib by mouth twice daily for 12 weeks and carboplatin at a dose aimed to achieve a pharmacologic area under the concentration-versus-time curve of 6 mg·hr per liter on weeks 1, 4, 7, and 10, concurrent with weekly paclitaxel. 2) segment 2 treatment: doxorubicin (60 mg per square meter) and cyclophosphamide (600 mg per square meter) intravenously every 2 to 3 weeks for four doses. | None | 20-24 | weekly |
| Schmid 2020 | pembrolizumab+[carboplatin+weekly paclitaxel->(doxorubicin+cyclophosphamide) or (epirubicin+cyclophosphamide)] | 1) segment 1 treatment: four cycles of an intravenous infusion of pembrolizumab (200mg) once every 3 weeks plus paclitaxel (80 mg/m2 of body-surface area once weekly) plus carboplatin (area under the curve, 5 mg/mL/min once every 3 weeks, or 1.5 mg/mL/min once weekly in the first 12 weeks). 2) segment 2 treatment: four cycles of pembrolizumab plus doxorubicin (60 mg/m2), or epirubicin (90 mg/m2) plus cyclophosphamide (600 mg/m2 once every 3 weeks in the subsequent 12 weeks). | Patients who either completed or discontinued the first neoadjuvant treatment could start the second neoadjuvant treatment or undergo surgery, and those who completed or discontinued the second neoadjuvant treatment could undergo surgery. Patients underwent definitive surgery (breast conservation or mastectomy with sentinel lymph-node evaluation or axillary dissection) 3 to 6 weeks after the last cycle of the neoadjuvant phase. Patients underwent definitive surgery (breast conservation or mastectomy with sentinel lymphnode evaluation or axillary dissection) 3 to 6 weeks after the last cycle of the neoadjuvant phase. In the adjuvant phase, patients received radiation therapy as indicated and pembrolizumab or placebo once every 3 weeks for up to nine cycles. Adjuvant capecitabine was not allowed according to the protocol. Trial treatment was discontinued in patients with disease progression or recurrence or unacceptable toxic effects. | 24 | weekly |
| Schmid 2020 | (carboplatin+weekly paclitaxel)->(doxorubicin+cyclophosphamide) or (epirubicin+cyclophosphamide) | 1) segment 1 treatment: four cycles of an intravenous infusion of placebo once every 3 weeks plus paclitaxel (80 mg/m2 of body-surface area once weekly) plus carboplatin (area under the curve, 5 mg/mL/min once every 3 weeks, 1.5 mg/mL/min once weekly in the first 12 weeks). 2) segment 2 treatment: four cycles of placebo plus doxorubicin (60 mg/m2), or epirubicin (90 mg/m2) plus cyclophosphamide (600 mg/m2 once every 3 weeks in the subsequent 12 weeks). | Patients who either completed or discontinued the first neoadjuvant treatment could start the second neoadjuvant treatment or undergo surgery, and those who completed or discontinued the second neoadjuvant treatment could undergo surgery. Patients underwent definitive surgery (breast conservation or mastectomy with sentinel lymph-node evaluation or axillary dissection) 3 to 6 weeks after the last cycle of the neoadjuvant phase. Patients underwent definitive surgery (breast conservation or mastectomy with sentinel lymphnode evaluation or axillary dissection) 3 to 6 weeks after the last cycle of the neoadjuvant phase. In the adjuvant phase, patients received radiation therapy as indicated and pembrolizumab or placebo once every 3 weeks for up to nine cycles. Adjuvant capecitabine was not allowed according to the protocol. Trial treatment was discontinued in patients with disease progression or recurrence or unacceptable toxic effects. | 24 | weekly |
| Schneeweiss 2019 | epirubicin->q2w paclitaxel->cyclophosphamide | 1) segment 1 treatment: epirubicin 150 mg/ m2 every 2 weeks (q2w) for three cycles. 2) segment 2 treatment: paclitaxel 225 mg/m2 q2w for three cycles. 3) segment 3 treatment: cyclophosphamide 2000 mg/m2 q2w for three cycles. | Treatment continued until surgery, disease progression, unacceptable toxicity, or withdrawal of consent. | 18 | 2-weekly |
| Schneeweiss 2019 | (weekly paclitaxel+non-pegylated liposomal doxorubicin)+carboplatin | paclitaxel 80 mg/m2 weekly plus non-pegylated liposomal doxorubicin (M) 20 mg/m2 weekly and carboplatin area under curve 1.5 weekly for 18 weeks. | Treatment continued until surgery, disease progression, unacceptable toxicity, or withdrawal of consent. | 18 | weekly |
| Sharma 2019 | (carboplatin+weekly paclitaxel)->(doxorubicin+cyclophosphamide) | 1) segment 1 treatment: paclitaxel 80 mg/m2 every week for 12 weeks plus carboplatin (AUC 6) every 3 weeks for 4 cycles 2) segment 2 treatment: doxorubicin 60 mg/m2 plus cyclophosphamide 600 mg/m2 every 2 weeks for 4 cycles | None | 20 | weekly |
| Sharma 2019 | carboplatin+docetaxel | carboplatin (AUC 6)+docetaxel (75 mg/m2) every 21 days X 6 cycles. | None | 18 | NA |
| Sikov 2015 | weekly paclitaxel->(doxorubicin+cyclophosphamide) | 1) segment 1 treatment: paclitaxel 80mg/m2 once per week (wP) for 12weeks. 2) segment 2 treatment: doxorubicin 60mg/m2 and cyclophosphamide 600 mg/m2 once every 2 weeks with myeloid growth factorsupport (ddAC) for four cycles. | After completing NACT, patients underwent repeat cardiac evaluation and reassessment of eligibility for BCS, followed by surgery, 4 to 8 weeks after cycle four of ddAC, thus at least 6 weeks after the last dose of bevacizumab. | 20 | weekly |
| Sikov 2015 | [weekly paclitaxel->(doxorubicin+cyclophosphamide)]+bevacizumab | 1) segment 1 treatment: paclitaxel 80mg/m2 onceperweek (wP)for 12 weeks. And bevacizumab 10 mg/kg once every 2 weeks for six cycles during administration of wP. 2) segment 2 treatment: doxorubicin 60mg/m2 and cyclophosphamide 600 mg/m2 once every 2 weeks with myeloid growth factorsupport (ddAC) for four cycles. And bevacizumab 10 mg/kg once every 2 weeks for three cycles during the first three cycles of ddAC. | After completing NACT, patients underwent repeat cardiac evaluation and reassessment of eligibility for BCS, followed by surgery, 4 to 8 weeks after cycle four of ddAC, thus at least 6 weeks after the last dose of bevacizumab. | 20 | weekly |
| Sikov 2015 | q4w carboplatin+weekly paclitaxel ->(doxorubicin+cyclophosphamide) | 1) segment 1 treatment: paclitaxel 80mg/m2 once per week (wP) for 12 weeks, Carboplatin AUC 6 once every 3 weeks × 4. 2) segment 2 treatment: doxorubicin 60mg/m2 and cyclophosphamide 600 mg/m2 once every 2 weeks with myeloid growth factorsupport (ddAC) for four cycles. | After completing NACT, patients underwent repeat cardiac evaluation and reassessment of eligibility for BCS, followed by surgery, 4 to 8 weeks after cycle four of ddAC, thus at least 6 weeks after the last dose of bevacizumab. | 20 | weekly |
| Sikov 2015 | [q4w carboplatin+weekly paclitaxel ->(doxorubicin+cyclophosphamide)]+bevacizumab | 1) segment 1 treatment: paclitaxel 80mg/m2 once per week (wP)for 12weeks, Carboplatin AUC 6 once every 3 weeks × 4. bevacizumab 10 mg/kg once every 2 weeks for six cycles during administration of wP. 2) segment 2 treatment: doxorubicin 60mg/m2 and cyclophosphamide 600 mg/m2 once every 2 weeks with myeloid growth factorsupport (ddAC) for four cycles. bevacizumab 10 mg/kg once every 2 weeks for three cycles during administration of the first three cycles of ddAC. | After completing NACT, patients underwent repeat cardiac evaluation and reassessment of eligibility for BCS, followed by surgery, 4 to 8 weeks after cycle four of ddAC, thus at least 6 weeks after the last dose of bevacizumab. | 20 | weekly |
| Tung 2020 | cisplatin | cisplatin 75 mg/m2 intravenously (IV) once every 3 weeks for 4 cycles. | None | 12 | NA |
| Tung 2020 | doxorubicin+cyclophosphamide | doxorubicin 60 mg/m2 with cyclophosphamide 600 mg/m2 once every 2-3 weeks for 4 cycles. | None | 8-12 | NA |
| Untch 2016 | weekly nab-paclitaxel->( epirubicin+cyclophosphamide ) | 1) segment 1 treatment: nab-paclitaxel (Abraxane; Celgene Corporation, Summit, NJ, USA) was given intravenously on days 1, 8, and 15, for four 3-week cycles initially at 150 mg/m². The dose was later reduced to 125 mg/m² based on a recommendation of the independent data monitoring committee after recruitment of 464 patients (protocol version March 28, 2013). 2) segment 2 treatment: received epirubicin 90 mg/m² intravenously plus cyclophosphamide 600 mg/m² intravenously on day 1 for four 3-week cycles. | None | 24 | weekly |
| Untch 2016 | weekly paclitaxel->(epirubicin+cyclophosphamide ) | 1) segment 1 treatment: solvent-based paclitaxel 80 mg/m² intravenously on days 1, 8, and 15, for four 3-week cycles.  2) segment 2 treatment: received epirubicin 90 mg/m² intravenously plus cyclophosphamide 600 mg/m² intravenously on day 1 for four 3-week cycles. | None | 24 | weekly |
| Von Minckwitz 2014 | bevacizumab+weekly paclitaxel+weekly non-pegylated liposomal doxorubicin+weekly carboplatin | paclitaxel 80 mg/m² plus non-pegylated liposomal doxorubicin 20 mg/m², carboplatin received the drug at a dose of 2·0 area under curve (AUC), given once a week for 18 weeks; bevacizumab 15 mg/kg intravenously every 3 weeks simultaneously with all cycles. The dose was reduced to AUC 1·5 after an interim safety analysis; 329 patients had been accrued by this point. The dose of carboplatin could be reduced to AUC 1·1 in case of intolerable toxic effects. | Permitted supportive treatments were dexamethasone (2–4 mg), 5HT3 inhibitors, clemastine, ranitidine, and loperamide as standby medication for patients receiving lapatinib, but no primary prophylaxis with G-CSF was recommended. In cases of tumour progression, the study treatment was discontinued and further local or systemic treatment was permitted at the discretion of the investi gator. Patients were scheduled for surgery within 21 days after last receipt of chemotherapy or after at least 28 days after the last bevacizumab infusion | 18 | weekly |
| Von Minckwitz 2014 | bevacizumab+weekly paclitaxel+non-pegylated liposomal doxorubicin | paclitaxel 80 mg/m² plus non-pegylated liposomal doxorubicin 20 mg/m², given once a week for 18 weeks; bevacizumab 15 mg/kg intravenously every 3 weeks simultaneously with all cycles. | Permitted supportive treatments were dexamethasone (2–4 mg), 5HT3 inhibitors, clemastine, ranitidine, and loperamide as standby medication for patients receiving lapatinib, but no primary prophylaxis with G-CSF was recommended. In cases of tumour progression, the study treatment was discontinued and further local or systemic treatment was permitted at the discretion of the investi gator. Patients were scheduled for surgery within 21 days after last receipt of chemotherapy or after at least 28 days after the last bevacizumab infusion | 18 | weekly |
| Wu 2018 | docetaxel+epirubicin | Epirubicin (E) 80 mg/m2 and Docetaxel (T) 75 mg/m2 once every 3 weeks×4. | None | 12 | NA |
| Wu 2018 | docetaxel+epirubicin+lobaplatin | Epirubicin (E) 80 mg/m2, Docetaxel (T) 75 mg/m2 and Lobaplatin (L) 30 mg/m2 once every 3 weeks×4 | None | 12 | NA |
| Zhang 2016 | carboplatin+q3w paclitaxel | paclitaxel 175 mg/m2 on day 1 plus carboplatin Area Under the Curve (AUC) = 5 on day 2, both administered via intravenous infusion (IV), every 3 weeks for 4-6 cycles. | 87 patients underwent surgery and had tumor responses evaluated pathologically. 75 patients underwent modified radical mastectomy and 12 patients had breastconserving surgery. 79 patients received at least 4 cycles of NAC, and 38 patients completed six cycles of NAC. 4 patients in PC arm switched to anthracycline-based adjuvant chemotherapy, and 13 patients in EP arm switched to platinum-based regimen after surgery due to lack of objective response in NAC. 23 patients in the PC arm and 21 patients in the EP arm received post-operative radiotherapy. All the patients who underwent breastconserving surgery received radiotherapy. | 12-18 | 3-weekly |
| Zhang 2016 | epirubicin+q3w paclitaxel | epirubicin 75 mg/m2 on day 1 and paclitaxel 175 mg/m2 on day 2, both IV, every 3 weeks for 4-6 cycles. | 87 patients underwent surgery and had tumor responses evaluated pathologically. 75 patients underwent modified radical mastectomy and 12 patients had breastconserving surgery. 79 patients received at least 4 cycles of NAC, and 38 patients completed six cycles of NAC. 4 patients in PC arm switched to anthracycline-based adjuvant chemotherapy, and 13 patients in EP arm switched to platinum-based regimen after surgery due to lack of objective response in NAC. 23 patients in the PC arm and 21 patients in the EP arm received post-operative radiotherapy. All the patients who underwent breastconserving surgery received radiotherapy. | 12-18 | 3-weekly |
| Zhang 2020 | docetaxel+carboplatin | docetaxel 75 mg/m2 plus carboplatin AUC 6 once every 3 weeks for 6 cycles (DCb) | None | 18 | NA |
| Zhang 2020 | (epirubicin+cyclophosphamide)->docetaxel | 1) segment 1 treatment: epirubicin 90 mg/m2 plus cyclophosphamide 600 mg/m2 once every 3 weeks for 4 cycles. 2) segment 2 treatment: docetaxel 75 mg/m2, once every 3 weeks for 4 cycles. (EC-D) | None | 24 | NA |

## Table S4 Characteristics of Outcomes

| **Study ID** | **Outcome** | **Definition/Measurement of Outcome** | **Length of Follow-up (Timepoint reported)** | **Is data in NMA** | **Data extracted from subgroup analysis of RCT** |
| --- | --- | --- | --- | --- | --- |
| Alba 2012 | pCR;  AE (grade 3-4) | 1) pCR was assessed at surgery following the Miller and Payne criteria. "Grade 5 was a complete pathological response (cPR) (ypT0/is ypN0). Residual ductal carcinoma in situ only was classified as a complete response" (Ogston, 2003). Clinical response was evaluated according to the RECIST criteria after the first sequence and before surgery. 2) Adverse events were graded following the National Cancer Institute Common Terminology Criteria for Adverse Events (NCI-CTCAE) version 3.0; the worst grade for each patient was reported. Patients with any grade 3-4 AE, excluding irregular menses. | 1) pCR: after the first sequence and before surgery (up to 24 weeks). 2) AE: at the time of surgery (up to 24 weeks). | Yes | No |
| Ando 2014 | pCR | A pCR was defined as the absence of viable invasive tumor in both the breast and axillary nodes. Patients with residual ductal carcinoma in situ (DCIS) in the breast and no viable invasive tumor in the axillary nodes were also classified as having a pCR (ypT0/is ypN0). Clinical response was evaluated by palpa- tion and caliper after each cycle according to the Response Evaluation Criteria in Solid Tumors version 1.1. | Clinical response was evaluated by palpation and caliper after each cycle. long-term: The median follow-up time is 12.0 months. | Yes | Yes |
| Bear 2012 | pCR | The primary end point was the rate of pathological complete response in the breast. A pathological complete response in the breast and nodes was defined as the absence of histologic evidence of invasive tumor cells in the surgical breast specimen, axillary nodes, and nonaxillary sentinel nodes identified after neo- adjuvant chemotherapy (ypT0/is ypN0). | pCR: [ Time Frame: Time of surgery, on average 6 or 13 months] after completion of the entire sequential chemotherapy program | No | Yes |
| Chen 2016 | pCR | pCR was defined as the absence of invasive tumor in the final surgical breast and axillary lymph nodes sample (ypT0/is ypN0). Standard Response Evaluation Criteria in Solid Tumors (RECIST) guidelines were used to evaluate clinical and pathological response | Physical examination and ultrasound were repeated every two cycles and performed before surgery. | Yes | Yes |
| Earl 2015 | pCR | Include pCR in all breast tumours and absence of disease in all removed axillary lymph nodes (ypT0/Tis ypN0) as a primary endpoint and pCR in all breast tumours (ypT0/Tis) as a secondary endpoint. | after neoadjuvant chemotherapy (18 weeks) | Yes | Yes |
| Fasching 2019 | pCR | 1) pCR rate (ypT0/is ypN0). 2) pCR rate (ypT0 ypN0). | Time Frame: 24 weeks | Yes | Yes |
| Gerber 2013 | pCR | pCR rates defined as no invasive and no noninvasive residuals in breast and lymph nodes; ypT0 ypN0. | post-chemotherapy (24 weeks) The breast tumor and regional lymph nodes were examined by palpation at every cycle and by sonographic examination after every second cycle; breast ultrasound, clinical examination and mammography were carried out before breast surgery. | Yes | Yes |
| Geyer 2017 | pCR;  AE (grade 3-4) | 1) Pathologic complete response (pCR) in breast and nodes. 2) Adverse events (AEs) were assessed with NCI CTCAE V4.0. | 1) Pathological Complete Response (pCR). [ Time Frame: At the time of definitive surgery (approximately 24-36 weeks from first dose of study drug). ] 2) AE follow-up was not specific on trial registrition. | Yes | No |
| Gianni 2018 | pCR | A pCR defined as absence of invasive cells in the breast and axillary nodes (ie, ypT0/is ypN0) at surgery after paclitaxel vs nab-paclitaxel, both followed by an anthracycline-containing regimen given as neoadjuvant therapy. | after neoadjuvant therapy (28 weeks) | Yes | Yes |
| Gigolaeva 2019 | pCR | pCR = ypT0/ypN0 or RCB 0-1 | After 2–3 cycles of chemotherapy patients underwent repeated examination: mammography, ultrasound, SPECT-CT and 2-nd biopsy and evaluation HR, Ki67 | Yes | No |
| Gluz 2018 | pCR;  serious AE | 1) pCR-defined as absence of invasive tumor cells in the breast and lymph nodes (ypT0/is ypN0), assessed by local pathologists—constituted the primary end point of the trial (in all patients and by “early response status”).  pCR (ypT0/ypN0). 2) Serious adverse events | 1) Time Frame: After 12 weeks of therapy  2) During treatment | No | No |
| Gonzalez-Angulo 2014 | pCR | Complete disappearance of the invasive carcinoma in the breast and axillary nodes at surgery was considered a pCR.  Tumor measurements were obtained by ultrasound at baseline, 12 weeks, and before surgery. | Time Frame: 24 weeks (time window -3 weeks) | Yes | No |
| Harbeck 2020 | pCR;  AE (grade 3-5) | 1) pathologic complete response (pCR, yp T0/is ypN0) 2) Grade 3-4 | 1) pCR: 21 weeks; 2) AE: baseling up to approximately 66 months. | Yes | No |
| Ishikawa 2016 | pCR | pCR was defined as necrosis and/or disappearance of all tumor cells and/or replacement of cancer cells by granulation and/or fibrosis. The pathological effect was determined using the definitions of pCR for primary tumors and lymph nodes (ypT0ypN0) | Time Frame: Time from start of treatment to 18 weeks | Yes | Yes |
| Jovanovic 2017 | pCR;  AE (grade 3-5) | 1) pCR defined as absence of invasive carcinoma in the breast and axillary lymph node. 2) Toxicity were categorized according to the National Cancer Institute CommonTerminology Criteria for Adverse Events (NCI-CTCAE) version 4. | Time Frame: at time of surgery, 15-18 weeks | No | No |
| Kummel 2017 | pCR | pCR rate defined as the complete absence of invasive carcinoma on histological examination of the breast irrespective of lymph node involvement (ypT0/is, ypN0/+) after the taxane treatment. | after neoadjuvant therapy (12 weeks) | No | Yes |
| Llombart-Cussac 2015 | pCR;  treatment-related AE (grade 3-4) | 1) pCR rate in the breast defined as the complete absence of invasive carcinoma on histological examination at the time of definitive surgery by an independent, blinded central pathology review in each arm of treatment. (The primary objective). pCR rate in the breast and axilla is the secondary objectives). 2) Safety assessments were performed weekly and included adverse event (AE) monitoring, hematology and biochemistry, vital signs, and physical examination. AEs were assessed according to the National Cancer Institute Common Ter- minology Criteria for Adverse Events (CTCAE) version 3.0. | 1) The cut-off date for the primary analysis was 30 days after the date of last patient surgery. 2) AE: Safety assessments were performed weekly and included adverse event (AE) monitoring, hematology and biochemistry, vital signs, and physical examination. Up to a maximum of 5 years after definitive surgery. | No | No |
| Loi 2019 | pCR;  serious AE | 1) pCR defined as no invasive residual disease in breast or nodes, non-invasive breast residuals allowed (pCRypT0/Tis ypN0) or no invasive or non-invasive residual disease in breast or nodes (pCRypT0 ypN0)]. 2) Adverse events were graded according to National Cancer Institute Common Terminology Criteria for Adverse Events version 4.0. The dose-limiting toxicity evaluation period comprised treatment cycle 1 through the first two cycles of combination regimen 1 (i.e. from cycle 1, day 1 through the end of cycle 3) and the first two cycles of combination regimen 2 (i.e. from cycle 6, day 1 through the end of cycle 7). Adverse events were monitored throughout the study and for up to 30 days after definitive surgery and 90 days for serious adverse events (or 30 days after definitive surgery if the patient initiated new anticancer therapy, whichever occurred first). | 1) after neoadjuvant chemotherapy 2) up to approximately 28 months | No | No |
| Loibl 2018 | pCR;  AE (grade 3-4) | 1) Pathological complete response was defined as the absence of residual invasive disease on evaluation of the resected breast specimen and resected lymph nodes following completion of neoadjuvant systemic therapy (ie, ypT0/is ypN0 per the American Joint Committee on Cancer staging system). 2) grade 3 or 4 treatment emergent adverse events. | 1) Time Frame: At the time of definitive surgery (approximately 24-36 weeks from first dose of study drug) 2) Adverse event monitoring was done from the time of administration of study drugs until 30 days following discontinuation of protocol treatment. Serious adverse events were collected from the time the patient signed the informed consent form. Laboratory monitoring (haematology, clinical chemistry, urinalysis, and coagulation) was done from baseline (28 days or less before first dose of study drug) to a final visit up to 30 days after the last dose of study drug. Laboratory assessments were done every 3 weeks during segment 1 and every 2- or 3-weeks during segment 2. | Yes | No |
| Loibl 2019 | pCR;  serious AE | 1) pCR defined as no invasive and no non-invasive tumour residuals in breast and in axillary lymph nodes (ypT0 ypN0) after neoadjuvant therapy. 2) Toxicity reported as adverse events (AEs) irrespective of relatedness to study treatment were based on NCI-CTC criteria v4.0. | 1) Time Frame: 22 weeks 2) During treatment | Yes | No |
| Martinez 2015 | pCR | pCR was defined by the absence of tumor cells in the breast and lymph nodes. | NR | Yes | No |
| Mayer 2019 | pCR | NR | after neoadjuvant chemotherapy (12 weeks) | No | No |
| Nahleh 2016 | pCR | pCR was defined as the absence of residual invasive disease with or without ductal carcinoma in situ (ypT0/isN0) in breast and axilla. | pCR evaluation was assessed at surgery, which was performed 3-6 weeks after completion of neoadjuvant chemotherapy. | Yes | Yes |
| Nanda 2020 | pCR | Pathologic complete response (pCR), defined as the absence of invasive tumor in breast and regional nodes at the time of surgery. | Time Frame: Post surgery based on up to 24-week treatment. | Yes | Yes |
| Rugo 2016 | pCR | Pathologic complete response (pCR), defined as the absence of invasive tumor in breast and regional nodes at the time of surgery. | Time Frame: Post surgery based on up to 24-week treatment. | Yes | Yes |
| Schmid 2020 | pCR;  AE (grade ≥ 3) | 1) In primary end point, pathological complete response is defined as pathological stage ypT0/Tis ypN0 at the time of definitive surgery.  In secondary end point, pathological complete response is defined as ypT0 ypN0 and ypT0/Tis in all patients. According to the current staging criteria of the American Joint Committee on Cancer and assessment by the local pathologist at the time of definitive surgery after completion of neoadjuvant systemic therapy, patients with pathological stage ypT0/Tis ypN0 have no residual invasive cancer in the complete resected breast specimen and all sampled regional lymph nodes, those with stage ypT0 ypN0 have no residual invasive and in situ cancer in the complete resected breast specimen and all sampled regional lymph nodes, and those with stage ypT0/Tis have no invasive cancer in the breast, irrespective of ductal carcinoma in situ or nodal involvement. 2) Adverse events were monitored throughout the trial and for 30 days after discontinuation of treat- ment (90 days for serious adverse events) and graded according to the Common Terminology Criteria for Adverse Events, version 4.0, of the National Cancer Institute. Immune-related ad- verse events were determined from a prespecified list of terms from the Medical Dictionary for Regula- tory Activities (MedDRA), which was updated with each new version of MedDRA. 2) Adverse events were monitored throughout the trial and for 30 days after discontinuation of treatment (90 days for serious adverse events) and graded according to the Common Terminology Criteria for Adverse Events, version 4.0, of the National Cancer Institute. | 1) pCR: [ Time Frame: Up to approximately 27-30 weeks]  2) Adverse events were monitored throughout the trial and for 30 days after discontinuation of treatment (90 days for serious adverse events). | Yes | No |
| Schneeweiss 2019 | pCR | The primary efficacy end-point was pCR defined as no residual invasive tumour cells in any resected specimens of the breast and axillary nodes (ypT0/is ypN0). Secondary short-term efficacy end-points included other pCR definitions (ypT0 ypN0; ypT0 ypN0/þ; ypT0/is ypN0/þ; ypT[any] ypN0). | pathological complete response (pCR= ypT0/is ypN0) [ Time Frame: 18 weeks (time window + 3 weeks)] | Yes | Yes |
| Sharma 2019 | pCR;  AE (grade 3-4) | 1) pCR is defined as no evidence of invasive tumor in the breast and axilla. 2) Grade 3-4 | 20 weeks | Yes | No |
| Sikov 2015 | pCR;  serious AE | 1) pCR breast was defined as the absence of residual invasive disease with or without ductal carcinoma insitu (ypT0/is).  pCR breast/axilla was defined as pCR breast and the absence of any tumor deposit 0.2 mm in sampled axillary nodes (ypT0/isN0).  2) serious AEs: any unexpected grade 3 toxicity or toxicity requiring hospitalization or surgical intervention. (as defined by Common Toxicity Criteria for Adverse Events, version 4.0) | 1) Time Frame: At the time of definitive surgical removal, up to 28 weeks 2) During treatment | Yes | No |
| Tung 2020 | pCR | Pathologic complete response (pCR) breast/axilla (ypT0/is, N0). Specific procedures were provided for evaluation of surgical specimens following neoadjuvant therapy (Appendix). | after neoadjuvant chemotherapy (8-12 weeks) | No | Yes |
| Untch 2016 | pCR | 1) pathological complete response, defined as no invasive or non-invasive tumour residuals in breast and axillary lymph nodes (ypT0 ypN0) after neoadjuvant therapy.  2) response assessment by other definitions for pathological complete response (no invasive tumour residuals in breast and axillary lymph nodes [ypT0/is ypN0], no invasive tumour residuals in breast [ypT0/is ypN0/+], and no invasive tumour residuals in axillary lymph nodes [ypN0]) and by clinical and imaging assessment after neoadjuvant therapy. | Time Frame: 24 weeks (time window + 3 weeks)  Evaluation of the primary tumour was done every second cycle and before surgery by ultrasound and if appropriate by mammography, MRI, or both. Haematology assessments (white blood cell, absolute neutrophil count, haemoglobin, and platelet count) were done every week, and biochemistry tests (including alkaline phosphatase, aspartate aminotransferase, alanine aminotransferase, bilirubin, and serum creatinine) done every 3 weeks and before surgery. | Yes | Yes |
| Von Minckwitz 2014 | pCR | 1) pathological complete response (ypT0 ypN0) after neoadjuvant treatment. 2) pathological complete response (ypT0/is ypN0) after neoadjuvant treatment. | Time Frame: 24 weeks (time window -3 weeks) | No | No |
| Wu 2018 | pCR | Pathologic CR in the breast and the axilla (pCR) was defined as pathologic CR in the breast with the absence of any tumor deposit ≥ 0.2 mm in sampled axillary nodes or with negative pretreatment sentinel lymph nodes. Pathologic CR in the breast was regarded as the complete disappearance of residual invasive disease with or without ductal carcinoma in situ by histopathologic examination. | after neoadjuvant chemotherapy (12 weeks) | No | No |
| Zhang 2016 | pCR | Pathologic complete response (pCR) rate is defined as no residual invasive cancer in both excised breast tissue and axillary lymph nodes, or only carcinoma in situ. Clinical responses were assessed every two cycles according to the Response Evaluation Criteria in Solid Tumors (RECIST version 1.0). | pCR: one week after surgery. The cut-off date for survival analysis was November 9th, 2015. | No | No |
| Zhang 2020 | pCR | pCR (yp T0/is ypN0) | NR | Yes | No |

# Appendix 3 Risk of bias in the included studies

Figure S1 presents the overall risk of bias summary for all included RCTs. Less than 15% of included trials reported adequate sequence generation and allocation concealment, which were rated as low risk of selection bias, and insufficient information was provided on other trials, which were rated as unclear risk on these two domains. Although almost half of included trials were open-label RCTs, risk of performance bias (blinding of patients and personnel) was rated as low, because pCR and aggregated AE were considered as objective outcomes. Insufficient information was provided on blinding of outcomes assessment in 71% of included trials, and was rated as unclear risk of detection bias. In terms of incomplete outcome data, drop-out rate in 40% of included trials were identified more than 20%, and attrition bias were rated as high risk; another 46% of included trials reported all complete data or drop-out rate less than 10% and reasons of drop-out were balance in compared groups, which were rated as low risk of attrition bias; insufficient information was provided on other trials, which were rated as unclear risk on this domain. All trials were rated as low risk of selective reporting bias except for two trials. 77% of included trials were funded by industry, and were rated as unclear risk of other bias; the other trials were not identified other risk of bias.

## Figure S1 Risk of Bias Summary


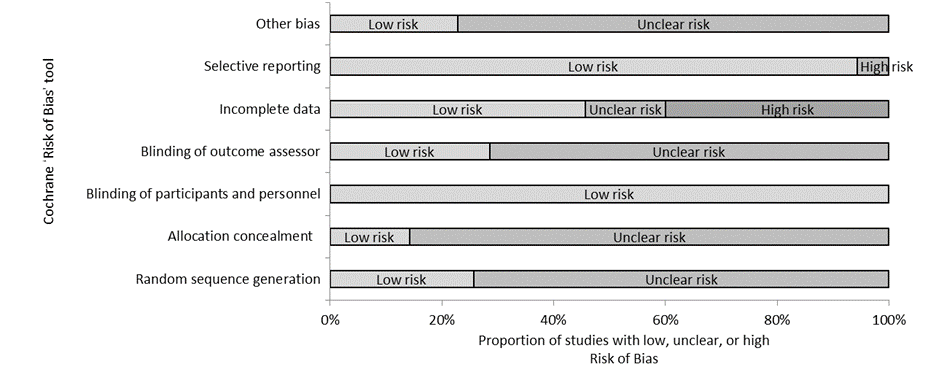


# Appendix 4 Citations for studies in Table 1

**Alba 2012**

Alba, E., et al., A randomized phase II trial of platinum salts in basal-like breast cancer patients in the neoadjuvant setting. Results from the GEICAM/2006-03, multicenter study. Breast Cancer Research and Treatment, 2012. 136(2): p. 487-493.

**Ando 2014**

Ando, M., et al., Randomized phase II study of weekly paclitaxel with and without carboplatin followed by cyclophosphamide/epirubicin/5-fluorouracil as neoadjuvant chemotherapy for stage II/IIIA breast cancer without HER2 overexpression. Breast cancer research and treatment, 2014. 145(2): p. 401-9.

**Bear 2012**

Bear, H.D., et al., Bevacizumab added to neoadjuvant chemotherapy for breast cancer. N Engl J Med, 2012. 366(4): p. 310-20.

**Chen 2016**

Chen, X., et al., Non-anthracycline-containing docetaxel and cyclophosphamide regimen is associated with sustained worse outcome compared with docetaxel, anthracycline and cyclophosphamide in neoadjuvant treatment of triple negative and HER2-positive breast cancer patients: updated follow-up data from NATT study. Chinese journal of cancer research = Chung-kuo yen cheng yen chiu, 2016. 28(6): p. 561-569.

Chen, X., et al., Superior outcome after neoadjuvant chemotherapy with docetaxel, anthracycline, and cyclophosphamide versus docetaxel plus cyclophosphamide: results from the NATT trial in triple negative or HER2 positive breast cancer. Breast cancer research and treatment, 2013. 142(3): p. 549‐558.

**Earl 2015**

Earl, H.M., et al., Efficacy of neoadjuvant bevacizumab added to docetaxel followed by fluorouracil, epirubicin, and cyclophosphamide, for women with HER2-negative early breast cancer (ARTemis): an open-label, randomised, phase 3 trial. Lancet Oncol, 2015. 16(6): p. 656-66.

**Fasching 2019**

Fasching, P.A., et al., GeparOLA: A randomized phase II trial to assess the efficacy of paclitaxel and olaparib in comparison to paclitaxel/carboplatin followed by epirubicin/cyclophosphamide as neoadjuvant chemotherapy in patients (pts) with HER2-negative early breast cancer (BC) and homologous recombination deficiency (HRD). Journal of Clinical Oncology, 2019. 37(15_suppl): p. 506-506.

**Gerber 2013**

Gerber, B., et al., Neoadjuvant bevacizumab and anthracycline-taxane-based chemotherapy in 678 triple-negative primary breast cancers; results from the geparquinto study (GBG 44). Annals of oncology : official journal of the European Society for Medical Oncology, 2013. 24(12): p. 2978-84.

von Minckwitz, G., et al., Neoadjuvant chemotherapy and bevacizumab for HER2-negative breast cancer. The New England journal of medicine, 2012. 366(4): p. 299-309.

Fasching, P.A., et al., BRCA1/2 Mutations and Bevacizumab in the Neoadjuvant Treatment of Breast Cancer: response and Prognosis Results in Patients With Triple-Negative Breast Cancer From the GeparQuinto Study. Journal of clinical oncology, 2018. 36(22): p. 2281‐2287.

Bevacizumab in neoadjuvant chemotherapy increases the pathological complete response rate in patients with triple-negative breast cancer. CA Cancer Journal for Clinicians, 2014. 64(3): p. 155-156.

**Geyer 2017**

Geyer, C.E., et al., Phase 3 study evaluating efficacy and safety of veliparib (V) plus carboplatin (Cb) or Cb in combination with standard neoadjuvant chemotherapy (NAC) in patients (pts) with early stage triple-negative breast cancer (TNBC). Journal of Clinical Oncology, 2017. 35(15).

**Gianni 2018**

Gianni, L., et al., Comparing Neoadjuvant Nab-paclitaxel vs Paclitaxel Both Followed by Anthracycline Regimens in Women With ERBB2/HER2-Negative Breast Cancer-The Evaluating Treatment With Neoadjuvant Abraxane (ETNA) Trial: a Randomized Phase 3 Clinical Trial. JAMA oncology, 2018. 4(3): p. 302‐308.

**Gigolaeva 2019**

Gigolaeva, L., et al., Neoadjuvant chemotherapy regimens for triple negative breast cancer patients. Breast, 2019. 44((Gigolaeva L.; Krivorotko P.; Zhiltsova E.; Dashyan G.; Chadjimatova S.; Pesotckiy R.; Emelyanov A.; Semiglazov V.) Breast Tumors, Petrov′s National Medical Research Center of Oncology, St Petersburg, Russian Federation): p. S70.

**Gluz 2018**

Gluz, O., et al., Comparison of Neoadjuvant Nab-Paclitaxel+Carboplatin vs Nab-Paclitaxel+Gemcitabine in Triple-Negative Breast Cancer: Randomized WSG-ADAPT-TN Trial Results. Journal of the National Cancer Institute, 2018. 110(6): p. 628-637.

Gluz, O., et al., Comparison of 12 weeks neoadjuvant Nab-paclitaxel combined with carboplatinum vs. gemcitabine in triple-negative breast cancer: WSG - ADAPT TN randomized phase II trial. Cancer research, 2016. 76(4).

Gluz, O., et al., Efficacy of 12 weeks neoadjuvant nab-paclitaxel combined with carboplatinum vs. gemcitabine in triple-negative breast cancer: WSG-ADAPT TN randomized phase II trial. Journal of Clinical Oncology, 2015. 33(15).

**Gonzalez-Angulo 2014**

Gonzalez-Angulo, A.M., et al., Open-label randomized clinical trial of standard neoadjuvant chemotherapy with paclitaxel followed by FEC versus the combination of paclitaxel and everolimus followed by FEC in women with triple receptor-negative breast cancer. Annals of oncology, 2014. 25(6): p. 1122‐1127.

**Harbeck 2020**

Harbeck, N.Z., H., et al., LBA11 - IMpassion031: Results from a phase III study of neoadjuvant (neoadj) atezolizumab + chemotherapy in early triple-negative breast cancer (TNBC). Annals of Oncology, 2020. 31: p. S1142-S1215.

**Ishikawa 2016**

Ishikawa, T., et al., BRCAness is beneficial for indicating triple negative breast cancer patients resistant to taxane. European Journal of Surgical Oncology (EJSO), 2016. 42(7): p. 999-1001.

**Jovanovic 2017**

Jovanović, B., et al., A Randomized Phase II Neoadjuvant Study of Cisplatin, Paclitaxel With or Without Everolimus in Patients with Stage II/III Triple-Negative Breast Cancer (TNBC): responses and Long-term Outcome Correlated with Increased Frequency of DNA Damage Response Gene Mutations, TNBC Subtype, AR Status, and Ki67. Clinical cancer research, 2017. 23(15): p. 4035‐4045.

**Kummel 2017**

Kümmel, S., et al., Randomised, open-label, phase II study comparing the efficacy and the safety of cabazitaxel versus weekly paclitaxel given as neoadjuvant treatment in patients with operable triple-negative or luminal B/HER2-negative breast cancer (GENEVIEVE). European journal of cancer (Oxford, England : 1990), 2017. 84: p. 1‐8.

**Llombart-Cussac 2015**

Llombart-Cussac, A., et al., SOLTI NeoPARP: a phase II randomized study of two schedules of iniparib plus paclitaxel versus paclitaxel alone as neoadjuvant therapy in patients with triple-negative breast cancer. Breast cancer research and treatment, 2015. 154(2): p. 351‐357.

**Loi 2019**

Loi, S., et al., Relationship between tumor infiltrating lymphocytes (TILs) and response to pembrolizumab (pembro) plus chemotherapy (CT) as neoadjuvant treatment (NAT) for triple-negative breast cancer (TNBC): Phase Ib KEYNOTE-173 trial. Annals of Oncology, 2019. 30.

**Loibl 2018**

Loibl, S., et al., Addition of the PARP inhibitor veliparib plus carboplatin or carboplatin alone to standard neoadjuvant chemotherapy in triple-negative breast cancer (BrighTNess): a randomised, phase 3 trial. The lancet. Oncology, 2018. 19(4): p. 497‐509.

**Loibl 2018**

Loibl, S., et al., A randomised phase II study investigating durvalumab in addition to an anthracycline taxane-based neoadjuvant therapy in early triple-negative breast cancer: clinical results and biomarker analysis of GeparNuevo study. Annals of oncology : official journal of the European Society for Medical Oncology, 2019. 30(8): p. 1279-1288.

**Martinez 2015**

Martinez, M.C.A., et al., Randomized phase II trial to evaluate the safety and efficacy of neoadjuvant cisplatin in combination with taxanesanthracyclines vs taxanesanthracyclines alone in locally advanced triple negative breast cancer. Journal of Clinical Oncology, 2015. 33(15).

**Mayer 2019**

Mayer, E.L., et al., TBCRC 030: A randomized phase II study of preoperative cisplatin versus paclitaxel in TNBC—Evaluating the homologous recombination deficiency (HRD) biomarker. Journal of Clinical Oncology, 2019. 37(15_suppl): p. 507-507.

**Nahleh 2016**

Nahleh, Z.A., et al., SWOG S0800 (NCI CDR0000636131): addition of bevacizumab to neoadjuvant nab-paclitaxel with dose-dense doxorubicin and cyclophosphamide improves pathologic complete response (pCR) rates in inflammatory or locally advanced breast cancer. Breast cancer research and treatment, 2016. 158(3): p. 485-95.

**Nanda 2020**

Nanda, R., et al., Effect of Pembrolizumab Plus Neoadjuvant Chemotherapy on Pathologic Complete Response in Women With Early-Stage Breast Cancer: An Analysis of the Ongoing Phase 2 Adaptively Randomized I-SPY2 Trial. JAMA oncology, 2020.

**Rugo 2016**

Rugo, H.S., et al., Adaptive Randomization of Veliparib-Carboplatin Treatment in Breast Cancer. New England journal of medicine, 2016. 375(1): p. 23‐34.

**Schmid 2020**

Schmid, P., et al., Pembrolizumab for Early Triple-Negative Breast Cancer. New England journal of medicine, 2020. 382(9): p. 810‐821.

Schmid, P., et al., KEYNOTE-522: Phase III study of pembrolizumab (pembro) + chemotherapy (chemo) vs placebo (pbo) + chemo as neoadjuvant treatment, followed by pembro vs pbo as adjuvant treatment for early triple-negative breast cancer (TNBC). Annals of Oncology, 2019. 30((Schmid P.) Centre for Experimental Cancer Medicine, Barts Cancer Institute-Queen Mary University of London, London, United Kingdom): p. v853-v854.

**Schneeweiss 2019**

Schneeweiss, A., et al., Intense dose-dense epirubicin, paclitaxel, cyclophosphamideversus weekly paclitaxel, liposomal doxorubicin (plus carboplatin in triple-negative breast cancer) for neoadjuvant treatment of high-risk early breast cancer (GeparOcto-GBG 84): A randomised phase III trial. European journal of cancer (Oxford, England : 1990), 2019. 106: p. 181-192.

**Sharma 2019**

Sharma, P., et al., Results of randomized phase II trial of neoadjuvant carboplatin plus docetaxel or carboplatin plus paclitaxel followed by AC in stage I-III triplenegative breast cancer (NCT02413320). Journal of Clinical Oncology, 2019. 37((Sharma P.; Kimler B.F.; O'Dea A.; Nye L.E.; Wang Y.Y.; Yoder R.; Prochaska L.H.; Wagner J.L.; Amin A.L.; Larson K.; Balanoff C.; Elia M.; Crane G.J.; Madhusudhana S.; Hoffmann M.S.; Sheehan M.; Rodriguez R.R.; Jensen R.A.; Godwin A.K.; Khan Q.J.) 1University of Kansas Medical Center, Kansas City, KS; University of Kansas Medical Center, Westwood, KS; Kansas University Medical Center, Westwood, KS; Univ of Kansas Medical Center, Kansas City, KS; University of Kansas Health System, Kansas City, KS; University of Kansas Medical Center, Overland Park, KS; University of Missouri, Kansas City, MO; The University of Kansas Cancer Center, Kansas City, KS).

**Sikov 2015**

Sikov, W.M., et al., Impact of the addition of carboplatin and/or bevacizumab to neoadjuvant once-per-week paclitaxel followed by dose-dense doxorubicin and cyclophosphamide on pathologic complete response rates in stage II to III triple-negative breast cancer: CALGB 40603 (Alliance). Journal of clinical oncology : official journal of the American Society of Clinical Oncology, 2015. 33(1): p. 13-21.

**Tung 2020**

Tung, N., et al., TBCRC 031: Randomized Phase II Study of Neoadjuvant Cisplatin Versus Doxorubicin-Cyclophosphamide in Germline BRCA Carriers With HER2-Negative Breast Cancer (the INFORM trial). Journal of clinical oncology : official journal of the American Society of Clinical Oncology, 2020. 38(14): p. 1539-1548.

**Untch 2016**

Untch, M., et al., Nab-paclitaxel versus solvent-based paclitaxel in neoadjuvant chemotherapy for early breast cancer (GeparSepto&#x2014;GBG 69): a randomised, phase 3 trial. The Lancet Oncology, 2016. 17(3): p. 345-356.

Furlanetto, J., et al., Efficacy and safety of nab-paclitaxel 125mg/m2 and nab-paclitaxel 150mg/m2 compared to paclitaxel in early high-risk breast cancer. Results from the neoadjuvant randomized GeparSepto study (GBG 69). Breast cancer research and treatment, 2017. 163(3): p. 495-506.

Von Minckwitz, G., et al., nab-paclitaxel at a dose of 125 mg/m2 weekly is more efficacious but less toxic than at 150 mg/m2. Results from the neoadjuvant randomized GeparSepto study (GBG 69). Cancer research, 2016. 76(4).

**von Minckwitz 2014**

von Minckwitz, G., et al., Neoadjuvant carboplatin in patients with triple-negative and HER2-positive early breast cancer (GeparSixto; GBG 66): a randomised phase 2 trial. The Lancet. Oncology, 2014. 15(7): p. 747-56.

Hahnen, E., et al., Germline Mutation Status, Pathological Complete Response, and Disease-Free Survival in Triple-Negative Breast Cancer: secondary Analysis of the GeparSixto Randomized Clinical Trial. JAMA oncology, 2017. 3(10): p. 1378‐1385.

Denkert, C., et al., Increased tumor-associated lymphocytes predict benefit from addition of carboplatin to neoadjuvant therapy for triple-negative and HER2-positive early breast cancer in the GeparSixto trial (GBG 66). Cancer research, 2013. 73(24).

**Wu 2018**

Wu, X., et al., A randomized and open-label phase II trial reports the efficacy of neoadjuvant lobaplatin in breast cancer. Nature communications, 2018. 9(1): p. 832.

**Zhang 2016**

Zhang, P., et al., Better pathologic complete response and relapse-free survival after carboplatin plus paclitaxel compared with epirubicin plus paclitaxel as neoadjuvant chemotherapy for locally advanced triple-negative breast cancer: a randomized phase 2 trial. Oncotarget, 2016. 7(37): p. 60647‐60656.

Zhang, P., et al., Carboplatin plus paclitaxel compared with epirubicin plus paclitaxel as neoadjuvant chemotherapy for triple-negative breast cancer - A phase II clinical trial. Cancer research, 2013. 73(24).

**Zhang 2020**

Zhang, L., et al., Neoadjuvant docetaxel + carboplatin versus epirubicin+cyclophosphamide followed by docetaxel in triple-negative, early-stage breast cancer (NeoCART): Results from a multicenter, randomized controlled, open-label, phase II trial. Journal of Clinical Oncology, 2020. 38(15_suppl): p. 586-586.

# Appendix 5 Results of network meta-analysis (NMA) for pCR

## Table S5 Number of Studies per treatment

| **AT** | **AT_plus_Atezo** | **AT_plus_Bev** | **AT_plus_Durva** | **AT_plus_Eve** | **AT_plus_Olaparib** |
| --- | --- | --- | --- | --- | --- |
| 19 | 1 | 4 | 1 | 1 | 1 |
| **AT_plus_Pembro** | **ATPt** | **ATPt_plus_Bev** | **ATPt_plus_Pembro** | **ATPt_plus_Veliparib** | **TC** |
| 1 | 11 | 1 | 1 | 3 | 2 |
| **TCb** |  |  |  |  |  |
| 2 |  |  |  |  |  |

## Table S6 Number of n-arm studies

| **2-arm** | **3-arm** | **4-arm** |
| --- | --- | --- |
| 19 | 2 | 1 |

## Table S7 Number of Studies per treatment comparison

| **Treatment 1** | **Treatment 2** | **Number of studies** |
| --- | --- | --- |
| AT | AT_plus_Atezo | 1 |
| AT | AT_plus_Bev | 4 |
| AT | AT_plus_Durva | 1 |
| AT | AT_plus_Eve | 1 |
| AT | AT_plus_Pembro | 1 |
| AT | ATPt_plus_Veliparib | 1 |
| AT | ATPt | 8 |
| AT | ATPt_plus_Bev | 1 |
| AT | ATPt_plus_Veliparib | 2 |
| AT | TC | 2 |
| AT | TCb | 1 |
| AT_plus_Bev | ATPt | 1 |
| AT_plus_Bev | ATPt_plus_Bev | 1 |
| AT_plus_Olaparib | ATPt | 1 |
| ATPt | ATPt_plus_Bev | 1 |
| ATPt | ATPt_plus_Pembro | 1 |
| ATPt | ATPt_plus_Veliparib | 2 |
| ATPt | TCb | 1 |

## Table S8 Overall heterogeneity and consistency

|  | **Overall *I^2^*** | **DIC** | Differences of DIC [should less than 5] |
| --- | --- | --- | --- |
| Fixed effect model Consistency model | 26% | 99.16076 | 1.64362 |
| Fixed effect model Non-consistency model | 27% | 100.80438 | - |

Notes: Fixed-effect consistency model was performed for final analysis

## Table S9 NMA results

Notes: OR (95%Crl) reported. Statistically significant was presented as bolding format, green presents favour intervention, and red presents favour control.

## Table S10 Ranking with SURCA

| **Ranks** | SUCRA |
| --- | --- |
| **AT_plus_Pembro** | 0.957043542 |
| **ATPt_plus_Pembro** | 0.899515208 |
| **ATPt_plus_Bev** | 0.757585000 |
| **TCb** | 0.594432292 |
| **ATPt_plus_Veliparib** | 0.586034375 |
| **ATPt** | 0.563406458 |
| **AT_plus_Atezo** | 0.518051042 |
| **AT_plus_Olaparib** | 0.471919375 |
| **AT_plus_Bev** | 0.369014583 |
| **AT_plus_Durva** | 0.325137500 |
| **AT_plus_Eve** | 0.318381667 |
| **AT** | 0.131807083 |
| **TC** | 0.007671875 |

## Figure S2 Ranking plot


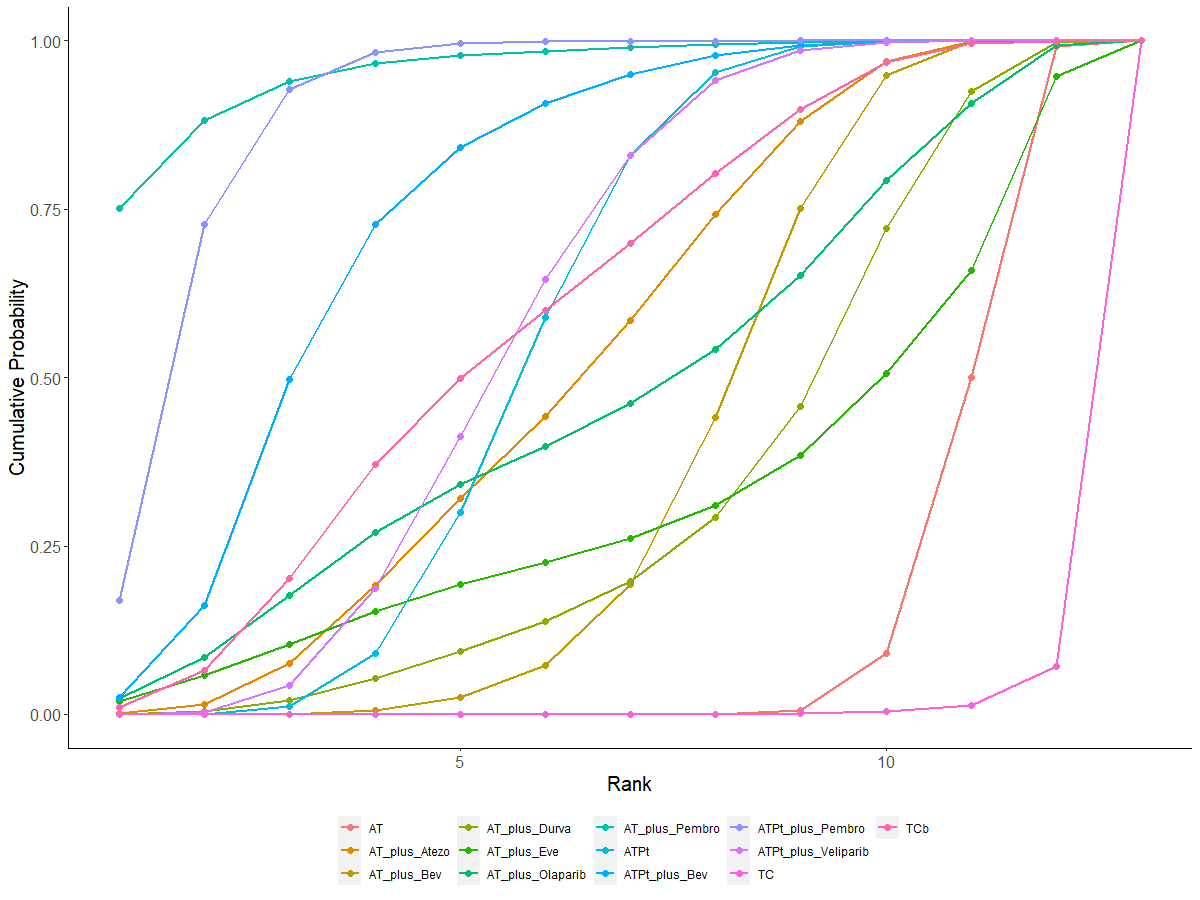


## Figure S3 Node-split plot


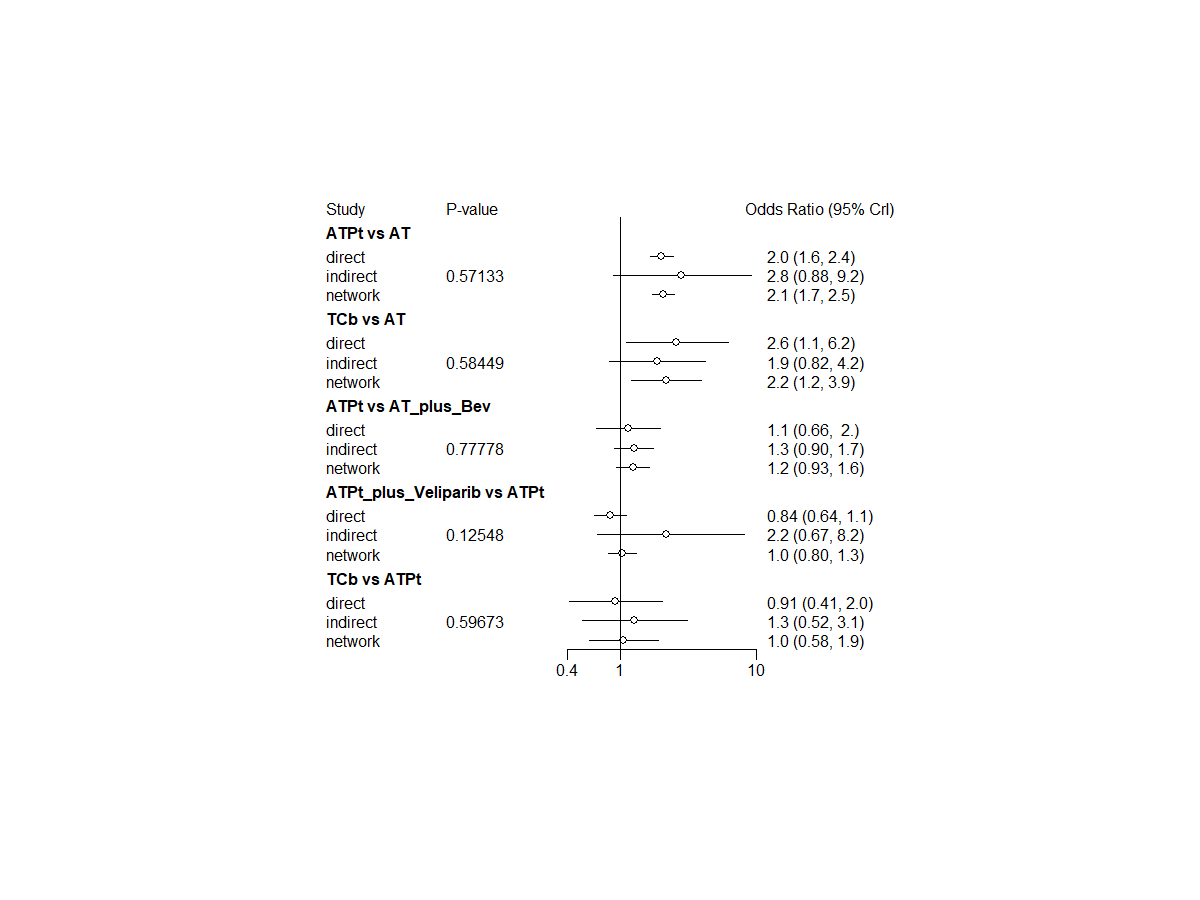


## Figure S4 Trace plot and density plot

PSRF=1.000319


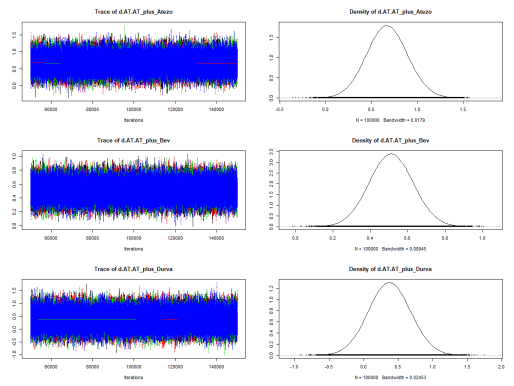

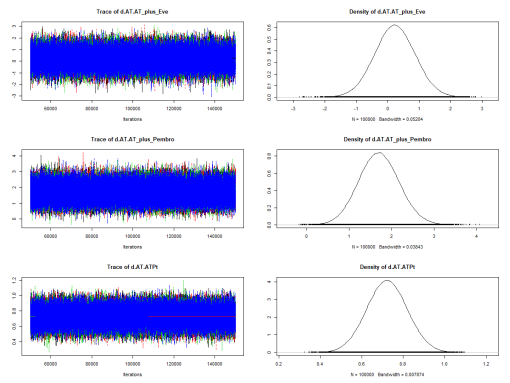

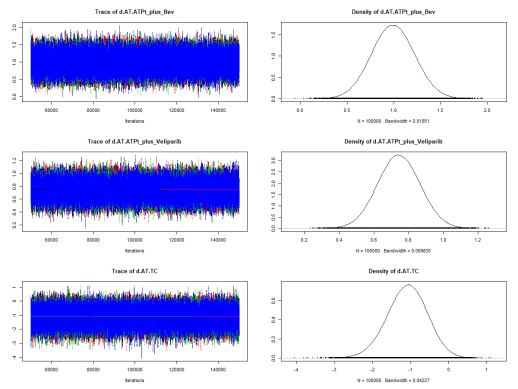


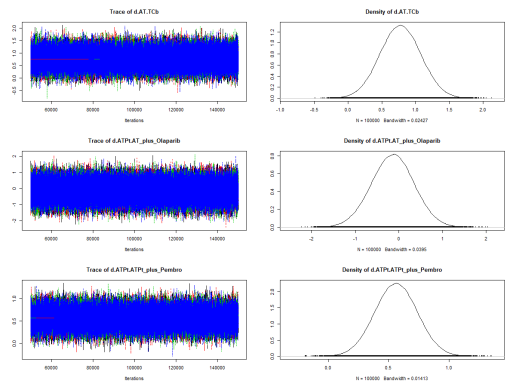

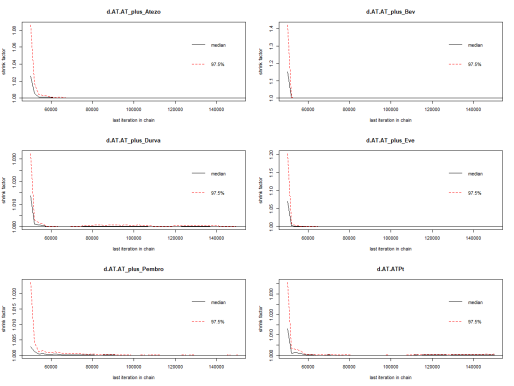

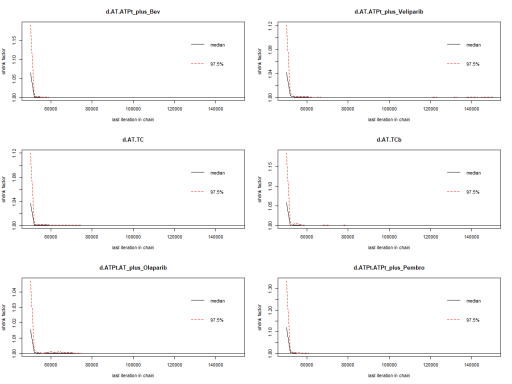


# Appendix 6 Results of NMA for aggregated AE

## Table S11 Number of Studies per treatment

| **AT** | **AT_plus_Bev** | **ATPt** | **ATPt_plus_Bev** | **ATPt_plus_Pembro** |
| --- | --- | --- | --- | --- |
| 3 | 1 | 5 | 1 | 1 |
| **ATPt_plus_Veliparib** | **TCb** |  |  |  |
| 2 | 1 |  |  |  |

## Table S12 Number of n-arm studies

| **2-arm** | **3-arm** | **4-arm** |
| --- | --- | --- |
| 2 | 2 | 1 |

## Table S13 Number of Studies per treatment comparison

| **Treatment 1** | **Treatment 2** | **Number of studies** |
| --- | --- | --- |
| AT | AT_plus_Bev | 1 |
| AT | ATPt | 3 |
| AT | ATPt_plus_Bev | 1 |
| AT | ATPt_plus_Veliparib | 2 |
| AT_plus_Bev | ATPt | 1 |
| AT_plus_Bev | ATPt_plus_Bev | 1 |
| ATPt | ATPt_plus_Bev | 1 |
| ATPt | ATPt_plus_Pembro | 1 |
| ATPt | ATPt_plus_Veliparib | 2 |
| ATPt | TCb | 1 |

## Table S14 Overall heterogeneity and consistency

|  | Overall *I^2^* | DIC | Differences of DIC [should less than 5] |
| --- | --- | --- | --- |
| Fixed effect model Consistency model | 51% | 37.64464 | 0.04539 |
| Fixed effect model Non-consistency model | 51% | 37.59925 | - |

Notes: Fixed-effect consistency model was performed for final analysis

## Table S15 NMA results

Notes: OR (95%Crl) reported. Statistically significant was presented as bolding format, green presents favour control, and red presents favour intervention

## Table S16 Ranking with SURCA

| **Ranks** | **SUCRA** |
| --- | --- |
| TCb | 0.9671125 |
| AT | 0.8662208 |
| ATPt | 0.5977083 |
| AT_plus_Bev | 0.3774125 |
| ATPt_plus_Veliparib | 0.2903583 |
| ATPt_plus_Pembro | 0.2245875 |
| ATPt_plus_Bev | 0.1766000 |

## Figure S5 Ranking plot


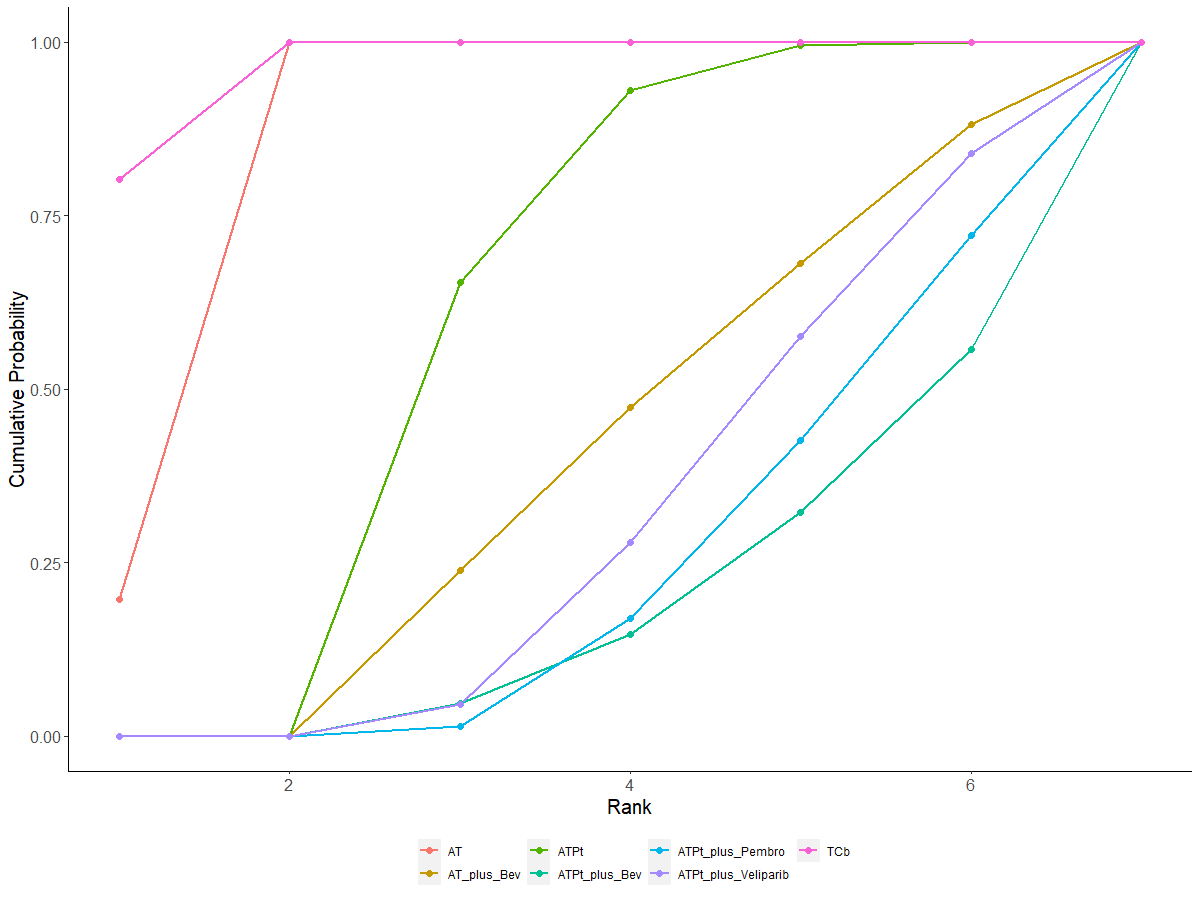


## Figure S6 Trace plot and density plot

PSRF=1.000558


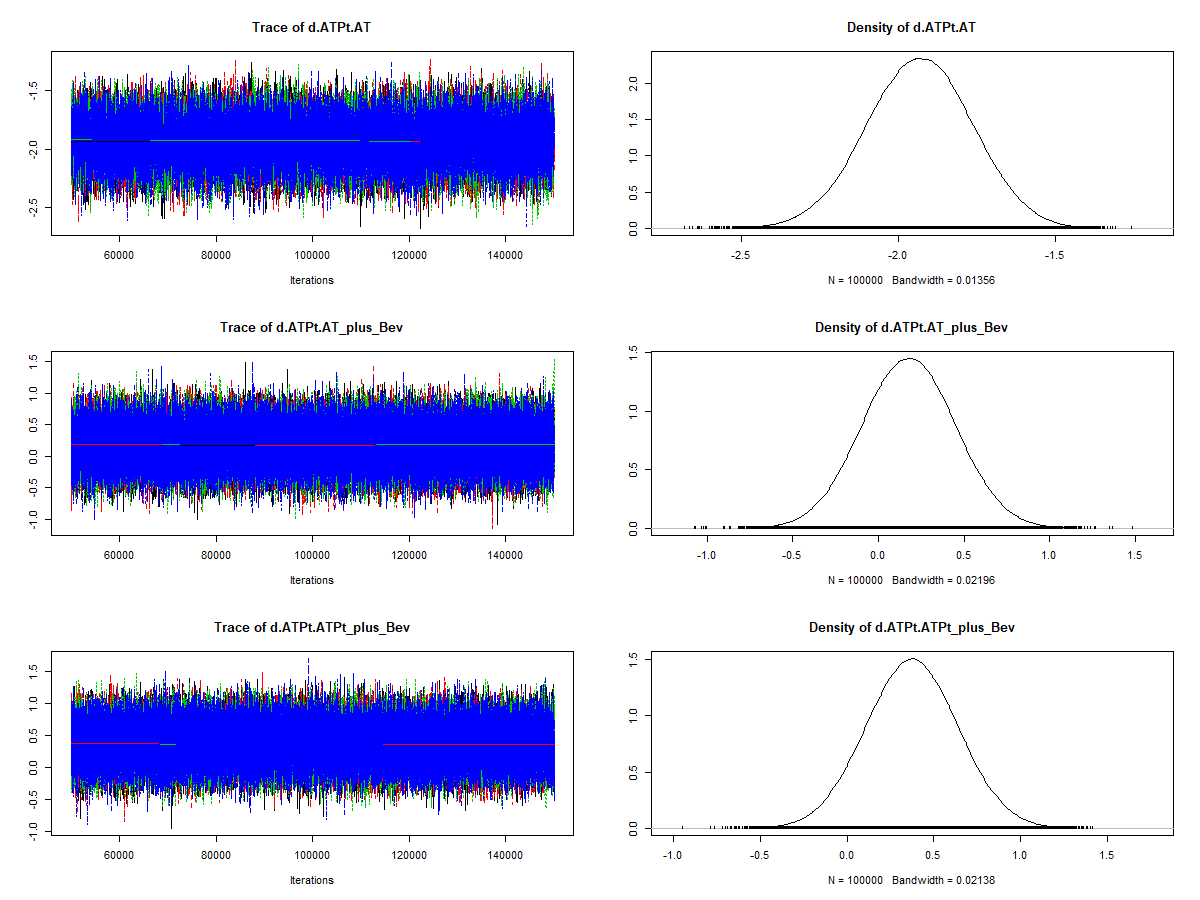

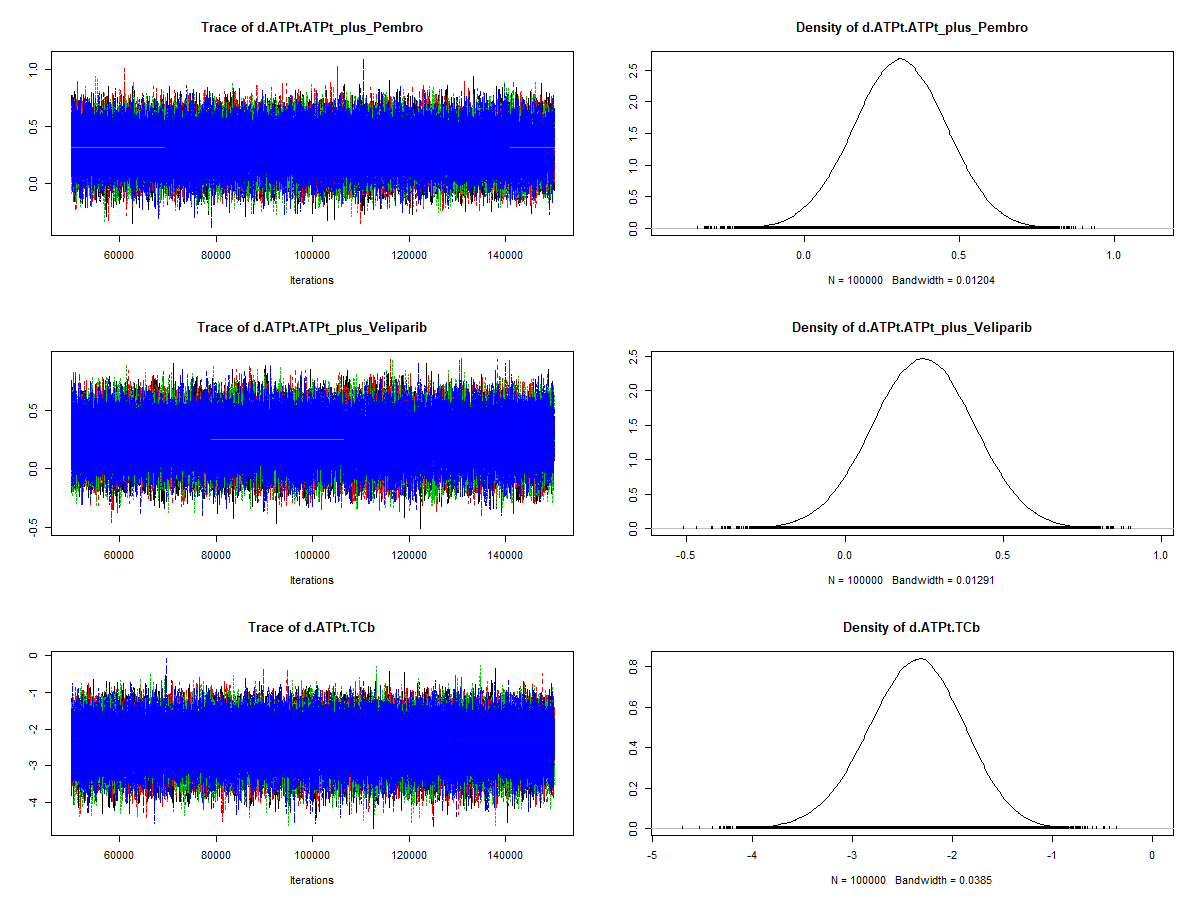


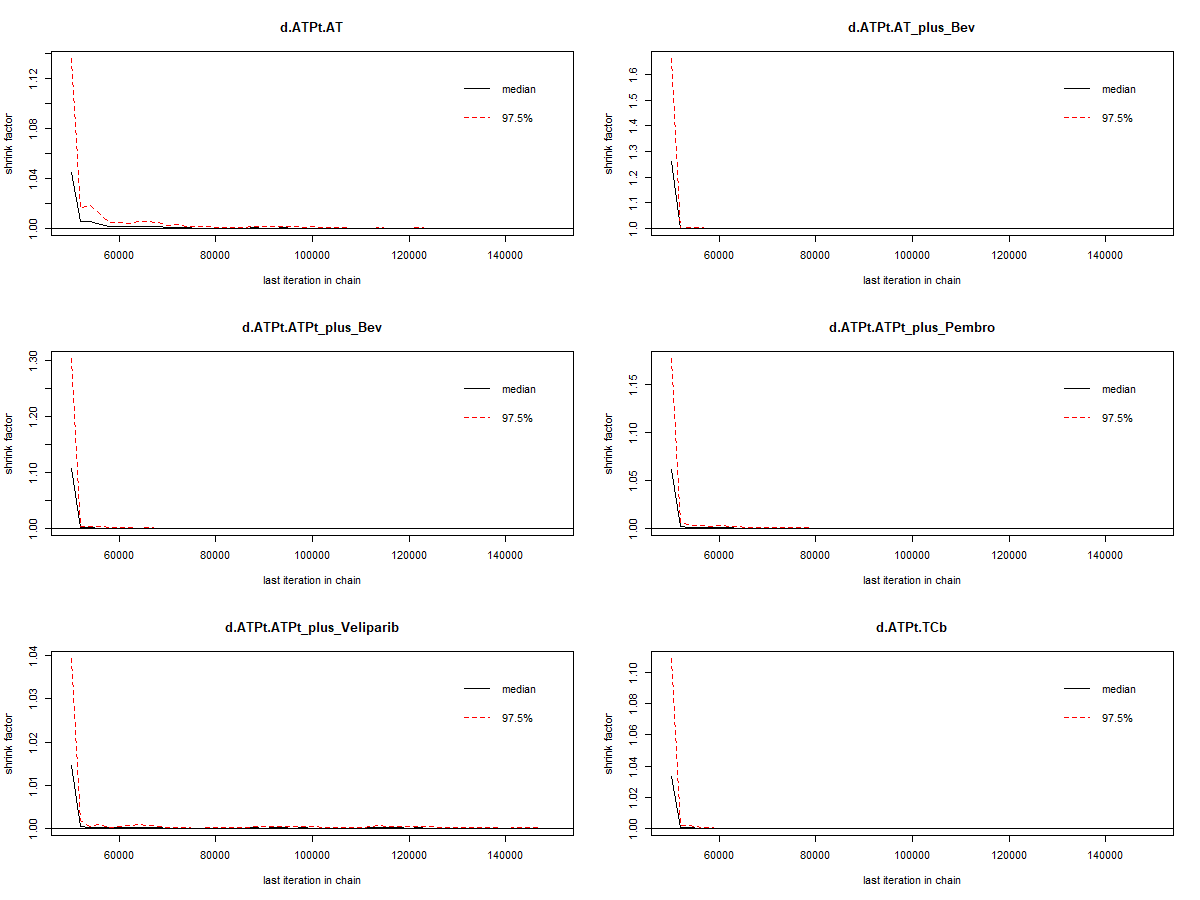


# Appendix 7 Results of NMA for pCR (detailed regimens)

## Figure S7 Network plot


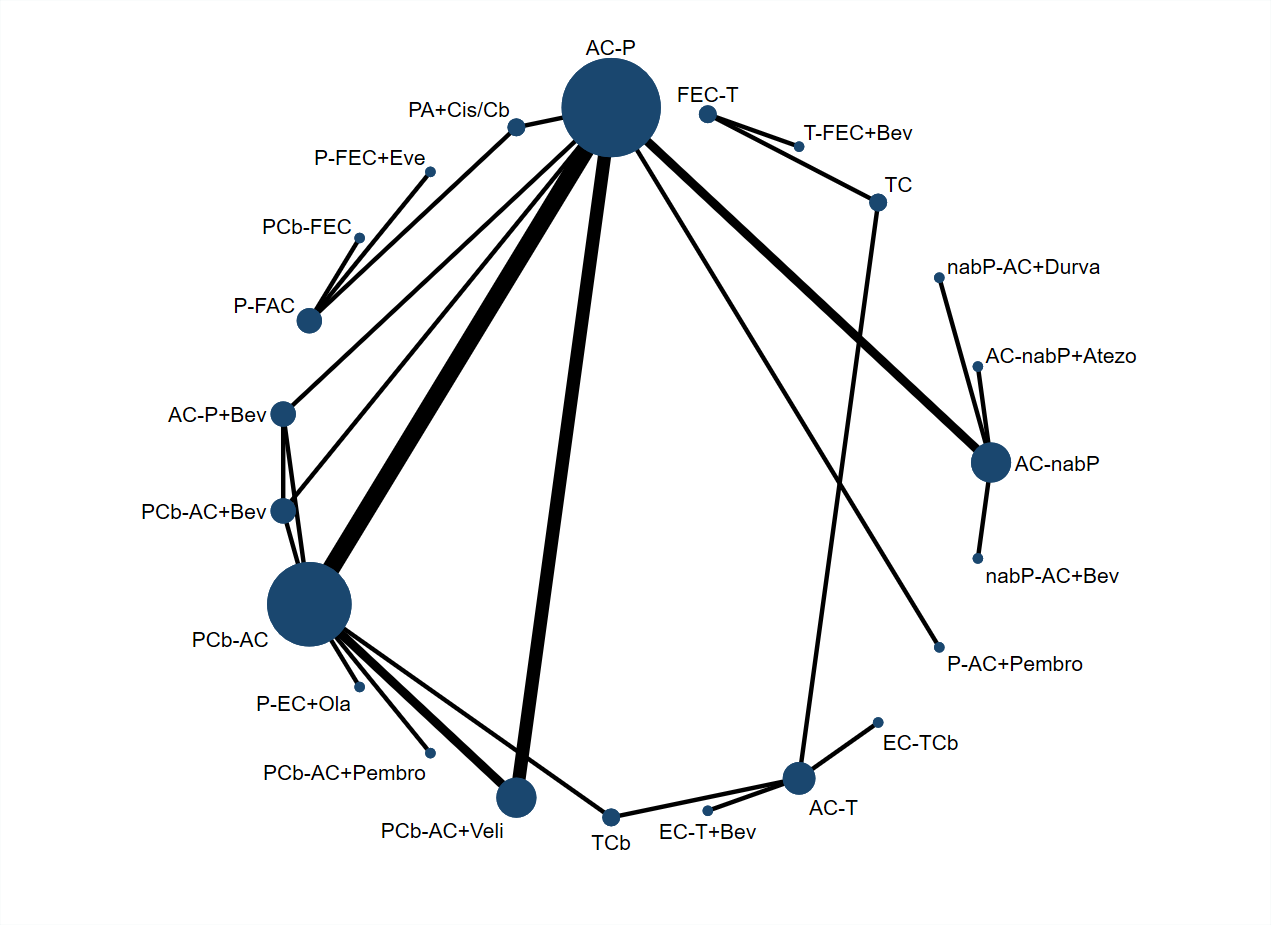


Notes: Direct comparisons are represented by the black lines connecting the neoadjuvant therapyregimens. Line width is proportional to thenumber of trials including every pair of neoadjuvant regimens, whereas circle size is proportional to the total number of trialsfor each neoadjuvant regimen in the network.A= doxorubicin; Atezo=atezolizumab; Bev=bevacizumab; C=cyclophosphamide; Cb=carboplatin; Cis=cisplatin; Durva=durvalumab; E=epirubicin; Eve=everolimus; F=5-fluorouracil; nabP=albumin paclitaxel (weekly cycle if not specially noted); Ola=olaparib; P=paclitaxel (weekly cycle if not specially noted); Pembro=pembrolizumab; T=docetaxel; Veli=veliparib.

## Table S17 Number of Studies per treatment

| **AC_nabP** | **AC_nabP_plus_Atezo** | **AC_P** |
| --- | --- | --- |
| 5 | 1 | 9 |
| **AC_P_plus_Bev** | **AC_T** | **Bev_plus_nabP_AC** |
| 1 | 4 | 1 |
| **Durva_plus_nabP_AC** | **EC_T_plus_Bev** | **EC_TCb** |
| 1 | 1 | 1 |
| **Eve_plus_P_FEC** | **FEC_T** | **P_AC_plus_Pembro** |
| 1 | 2 | 1 |
| **P_FAC** | **P_plus_Olaparib_EC** | **PA_plus_Cis_or_Cb** |
| 3 | 1 | 2 |
| **PCb_AC** | **PCb_AC_plus_Bev** | **PCb_AC_plus_Pembro** |
| 7 | 1 | 1 |
| **PCb_FEC** | **PCb_plus_Veliparib_AC** | **T_FEC_plus_Bev** |
| 1 | 3 | 1 |
| **TC** | **TCb** |  |
| 2 | 2 |  |

## Table S18 Number of n-arm studies

| **2-arm** | **3-arm** | **4-arm** |
| --- | --- | --- |
| 21 | 2 | 1 |

## Table S19 Number of Studies per treatment comparison

| **Treatment 1** | **Treatment 2** | **Number of studies** |
| --- | --- | --- |
| AC_nabP | AC_nabP_plus_Atezo | 1 |
| AC_nabP | AC_P | 2 |
| AC_nabP | Bev_plus_nabP_AC | 1 |
| AC_nabP | Durva_plus_nabP_AC | 1 |
| AC_P | AC_P_plus_Bev | 1 |
| AC_P | P_AC_plus_Pembro | 1 |
| AC_P | PA_plus_Cis_or_Cb | 1 |
| AC_P | PCb_AC | 4 |
| AC_P | PCb_AC_plus_Bev | 1 |
| AC_P | PCb_plus_Veliparib_AC | 3 |
| AC_P_plus_Bev | PCb_AC | 1 |
| AC_P_plus_Bev | PCb_AC_plus_Bev | 1 |
| AC_T | EC_T_plus_Bev | 1 |
| AC_T | EC_TCb | 1 |
| AC_T | TC | 1 |
| AC_T | TCb | 1 |
| Eve_plus_P_FEC | P_FAC | 1 |
| FEC_T | T_FEC_plus_Bev | 1 |
| FEC_T | TC | 1 |
| P_FAC | PA_plus_Cis_or_Cb | 1 |
| P_FAC | PCb_FEC | 1 |
| P_plus_Olaparib_EC | PCb_AC | 1 |
| PCb_AC | PCb_AC_plus_Bev | 1 |
| PCb_AC | PCb_AC_plus_Pembro | 1 |
| PCb_AC | PCb_plus_Veliparib_AC | 2 |
| PCb_AC | TCb | 1 |

## Table S20 Overall heterogeneity and consistency

|  | **Overall *I^2^*** | **DIC** | Differences of DIC [should less than 5] |
| --- | --- | --- | --- |
| Fixed effect model Consistency model | 12% | 104.34077 | 0.05281 |
| Fixed effect model Non-consistency model | 12% | 104.28796 | - |

Notes: Fixed-effect consistency model was performed for final analysis

## Table S21 NMA results

Notes: OR (95%Crl) reported. Statistically significant was presented as bolding format, green presents favour intervention, and red presents favour control.

## Figure S8 Forest plot for all treatments compared with key regimens (A. AC-P; B. PCb-AC+Pembro; C. TCb)

*
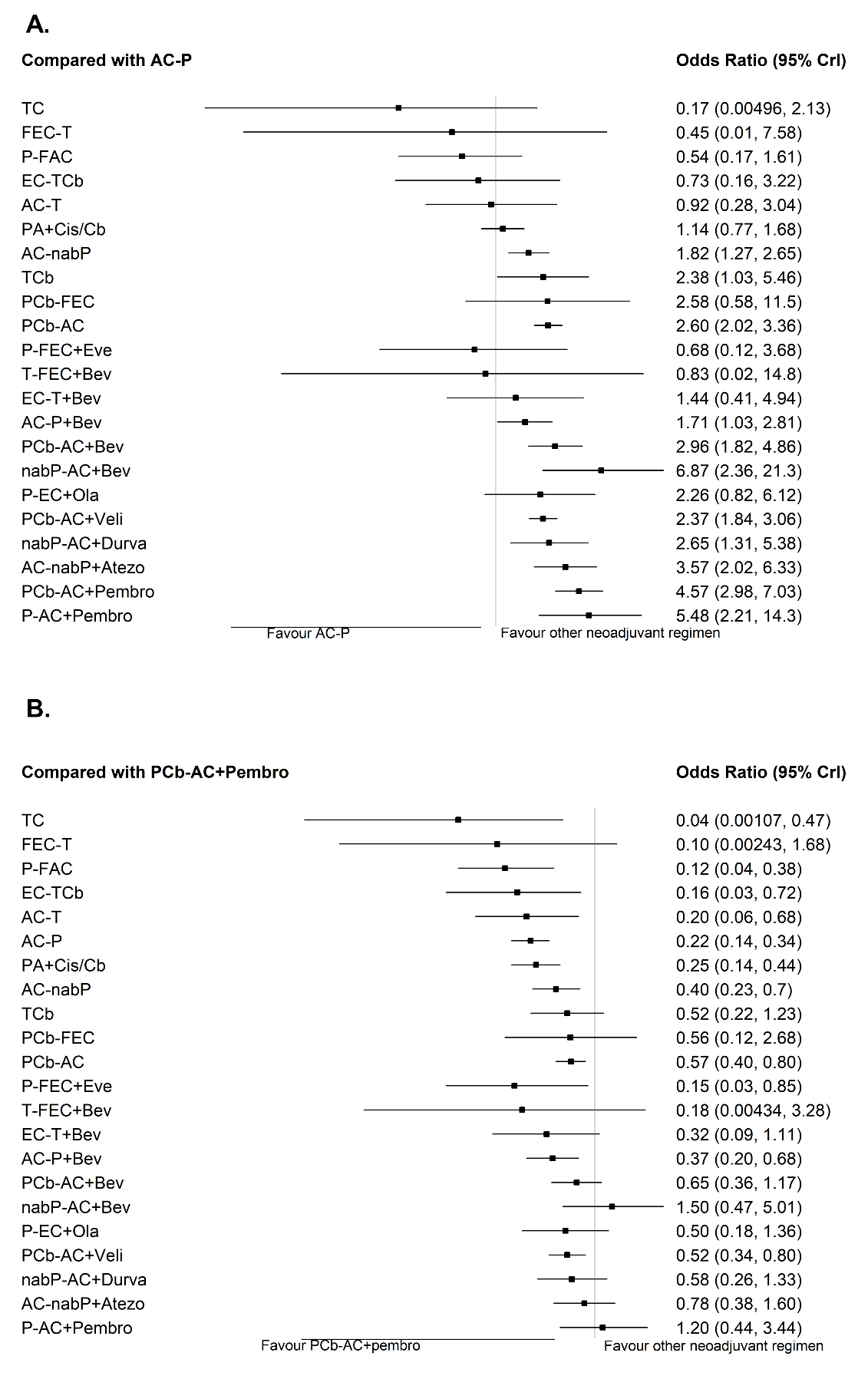
*


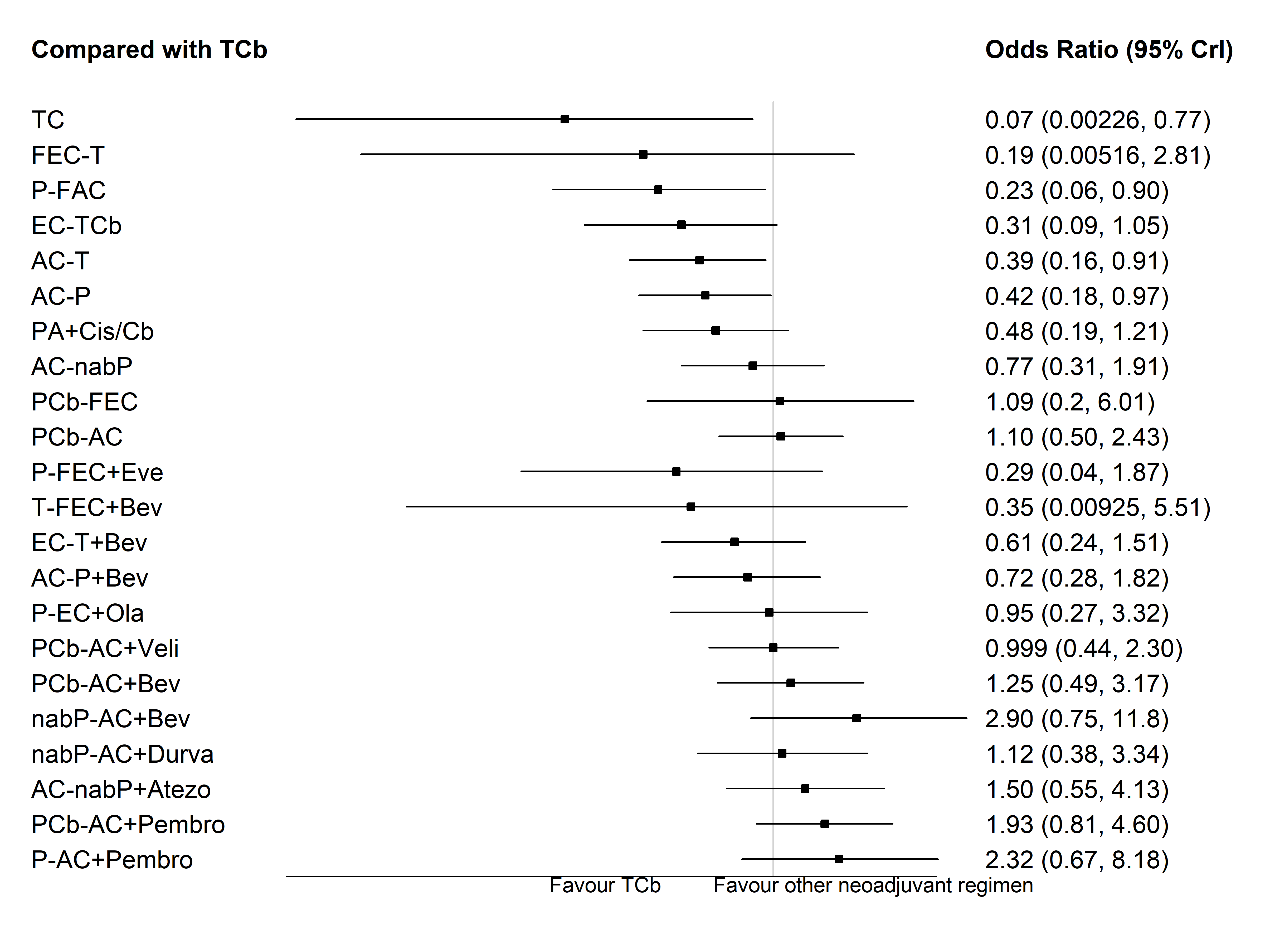


## Table S22 Ranking with SURCA

| **Ranks** | SUCRA |
| --- | --- |
| Bev_plus_nabP_AC | 0.93527102 |
| P_AC_plus_Pembro | 0.89893591 |
| PCb_AC_plus_Pembro | 0.88768500 |
| AC_nabP_plus_Atezo | 0.79770886 |
| PCb_AC_plus_Bev | 0.72552864 |
| PCb_AC | 0.66977068 |
| Durva_plus_nabP_AC | 0.66581659 |
| PCb_FEC | 0.64132659 |
| TCb | 0.62706852 |
| PCb_plus_Veliparib_AC | 0.61046943 |
| P_plus_Olaparib_EC | 0.59169989 |
| AC_nabP | 0.48208045 |
| AC_P_plus_Bev | 0.45822500 |
| EC_T_plus_Bev | 0.44111239 |
| T_FEC_plus_Bev | 0.37000830 |
| PA_plus_Cis_or_Cb | 0.30916455 |
| AC_T | 0.26682693 |
| AC_P | 0.25742500 |
| Eve_plus_P_FEC | 0.23096830 |
| EC_TCb | 0.22156602 |
| FEC_T | 0.21585205 |
| P_FAC | 0.14544170 |
| TC | 0.05004818 |

## Figure S9 Ranking plot


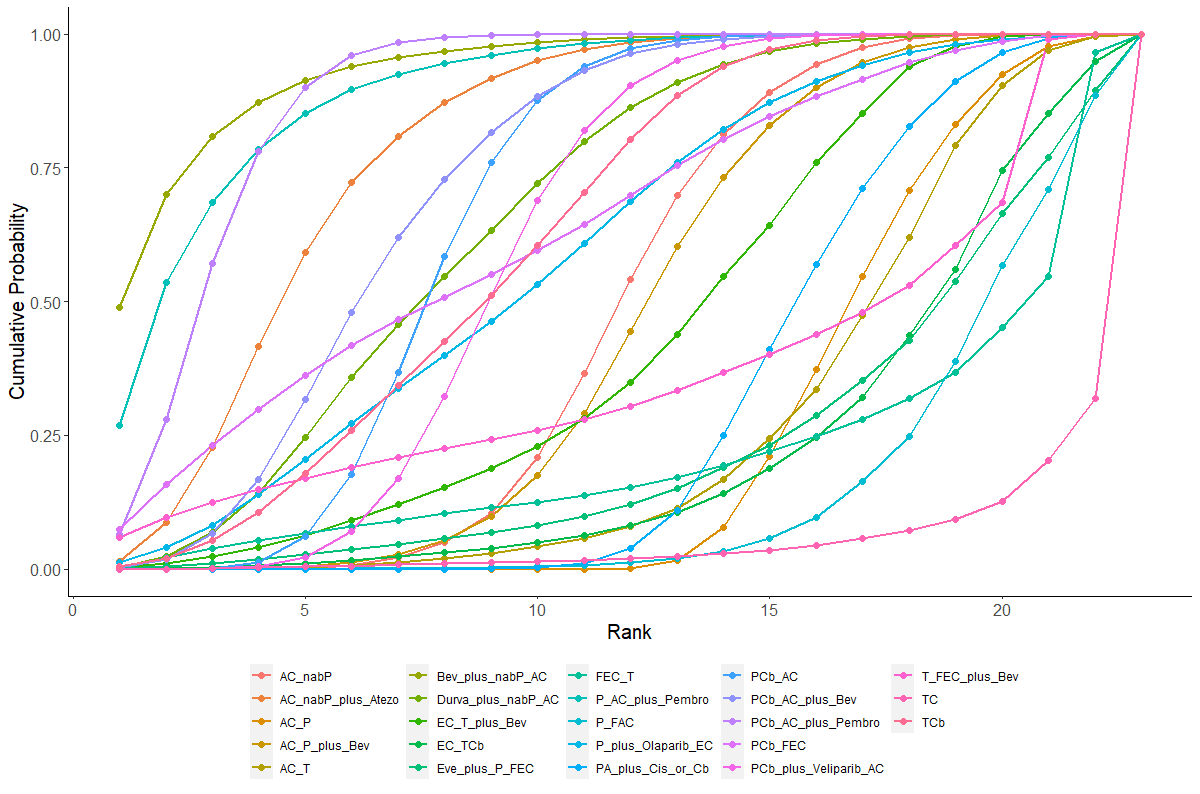


## Figure S10 Node-split plot


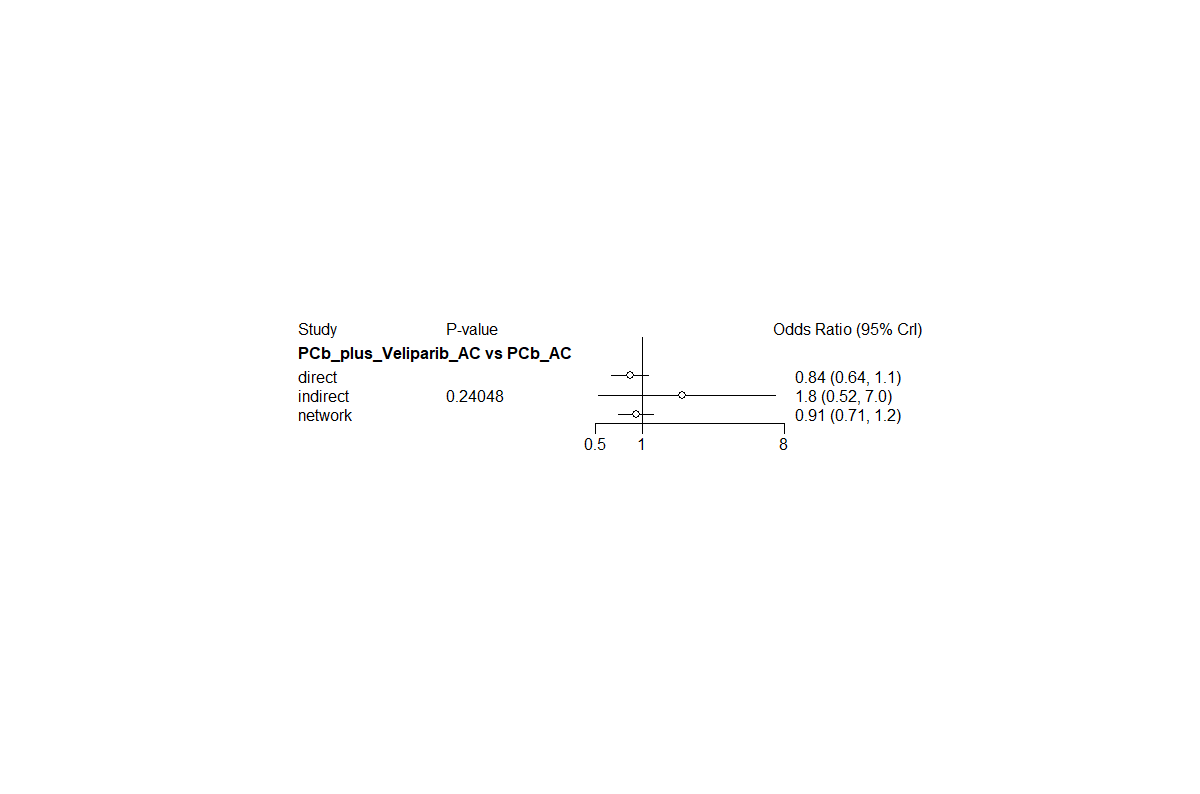


## Figure S11 Trace plot and density plot

PSRF=1.0005333


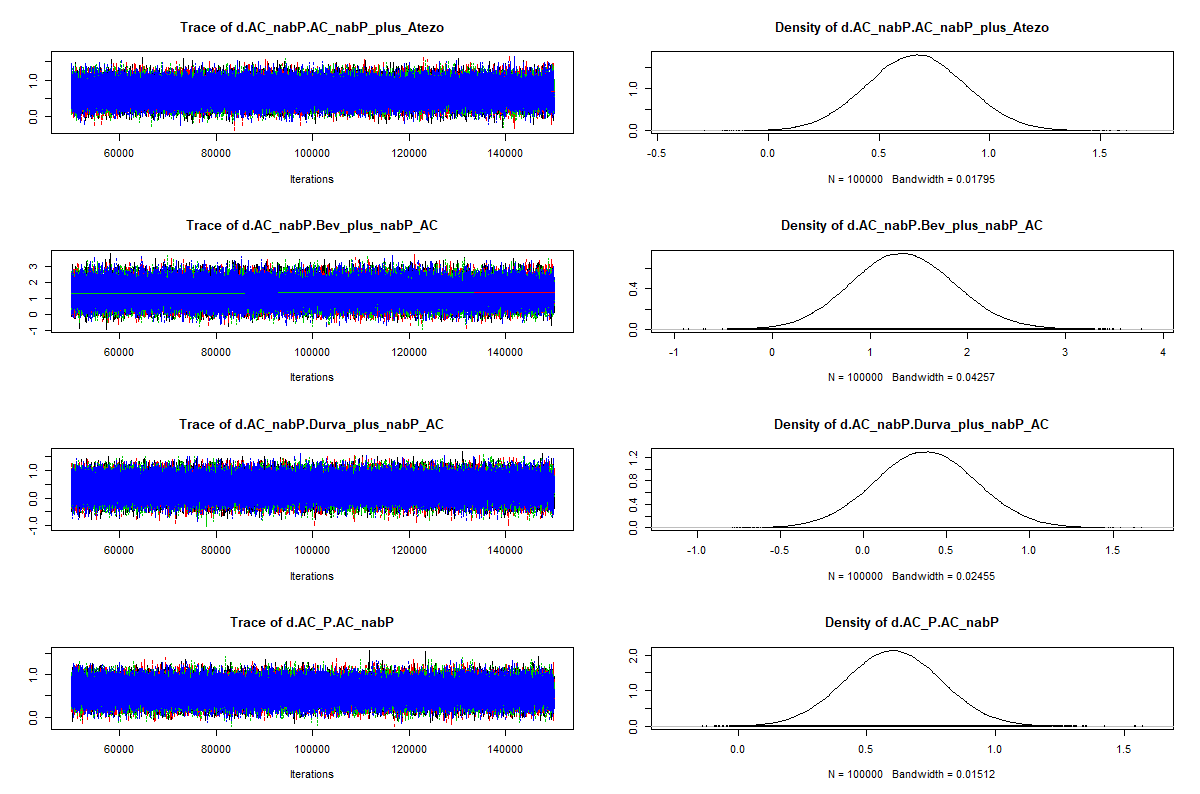

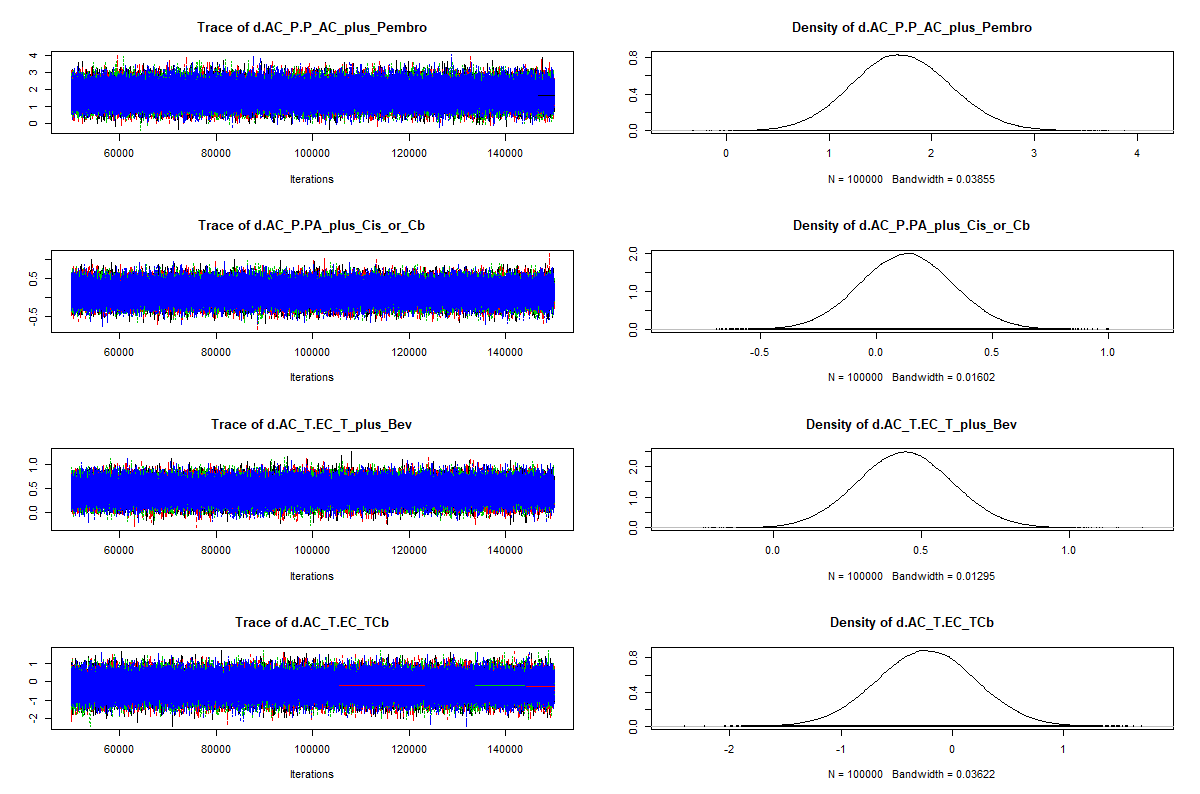


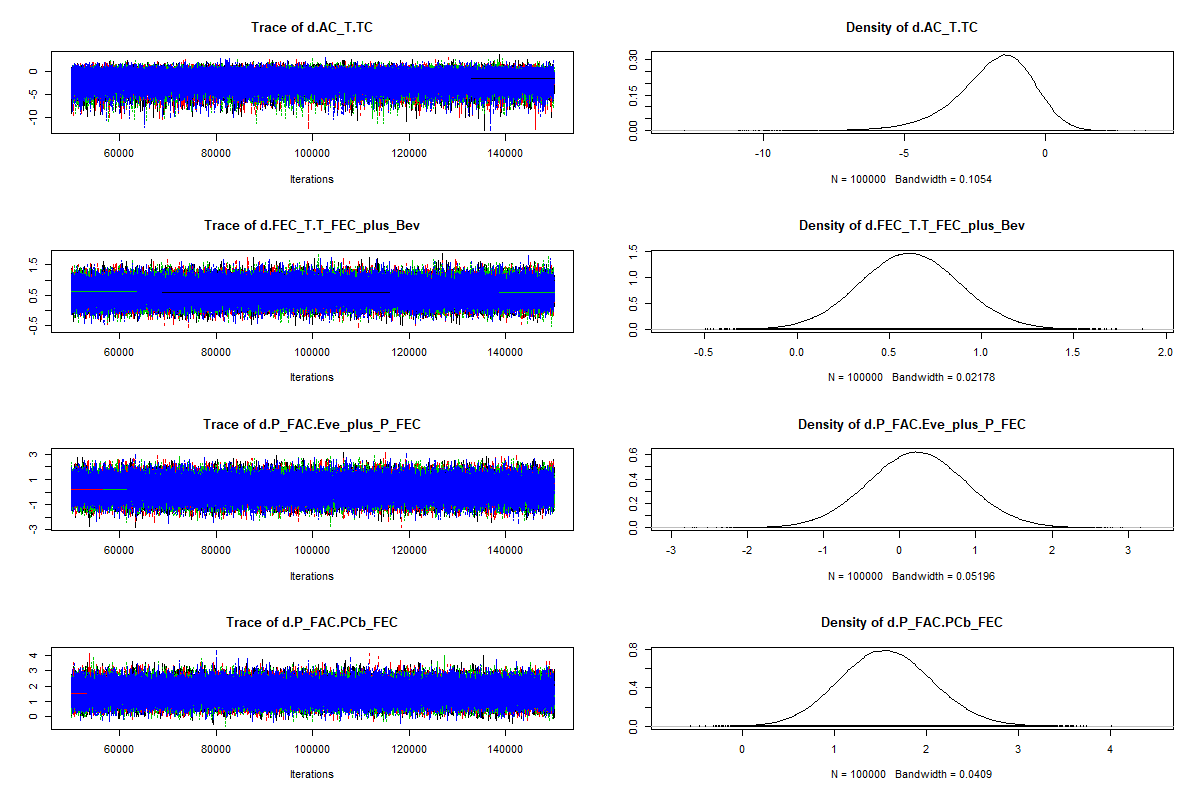

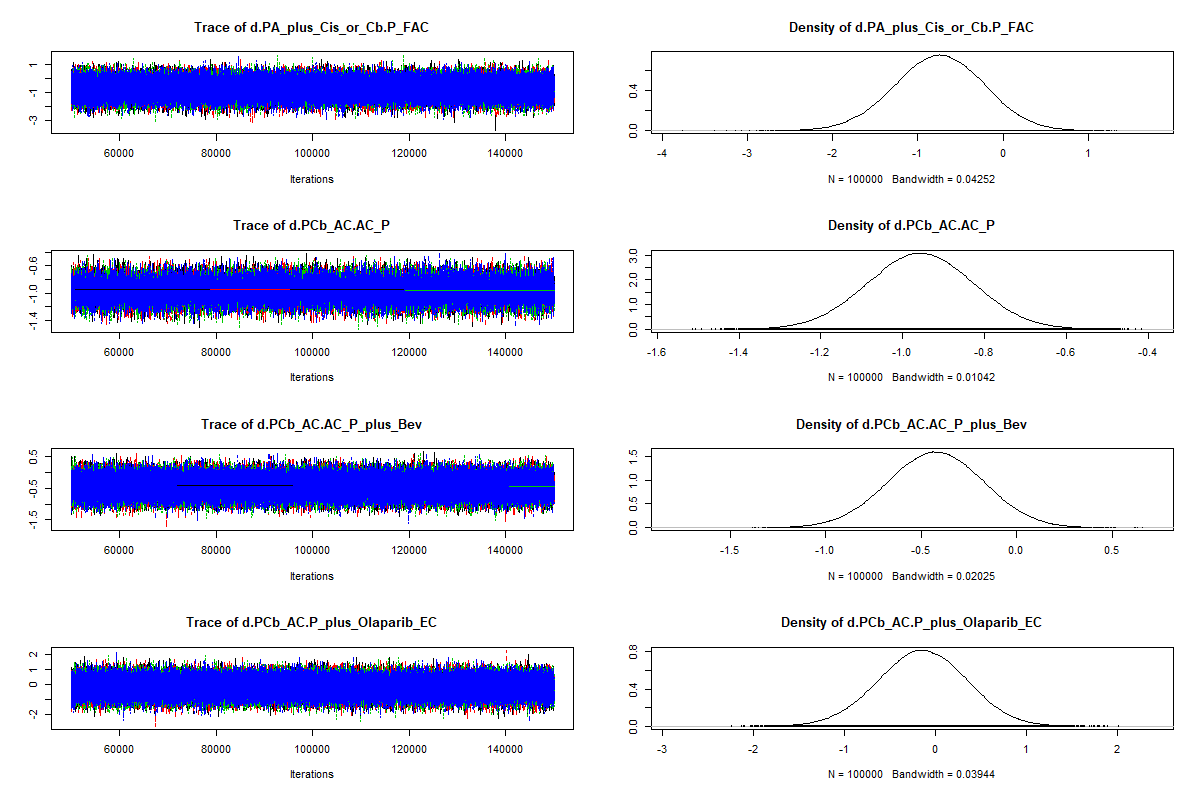


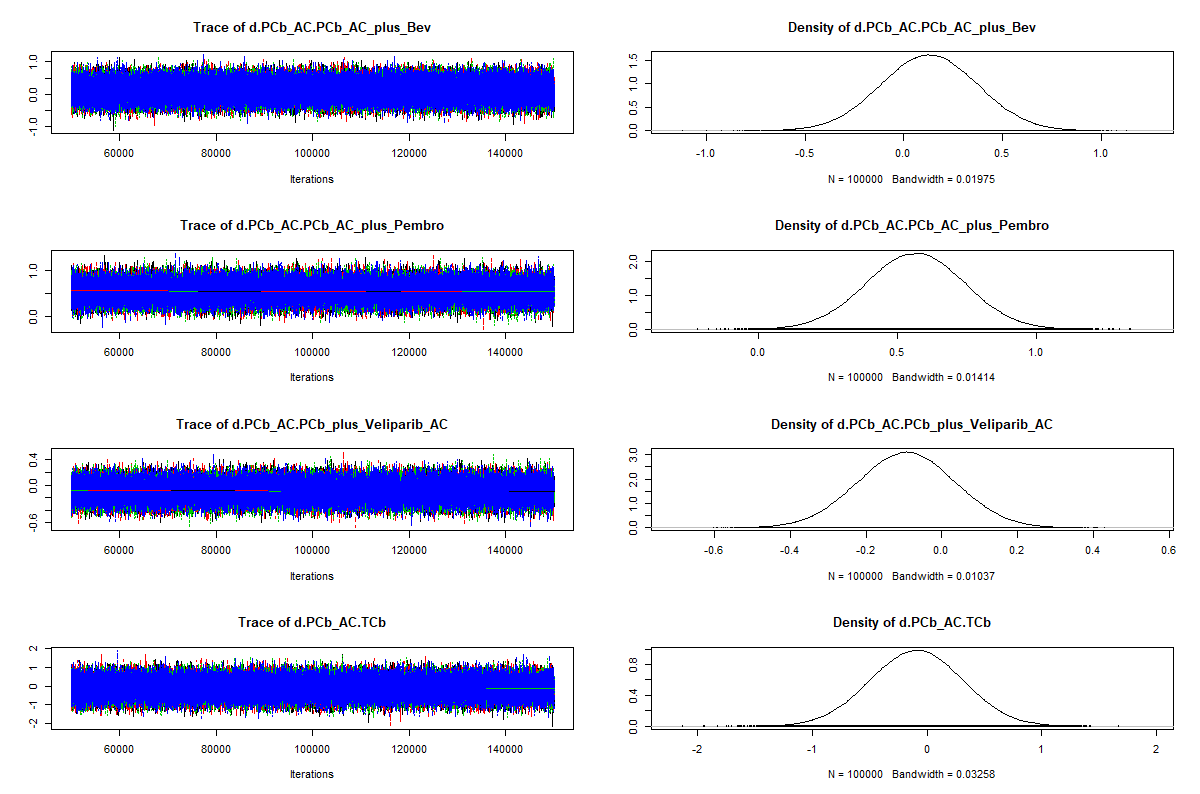

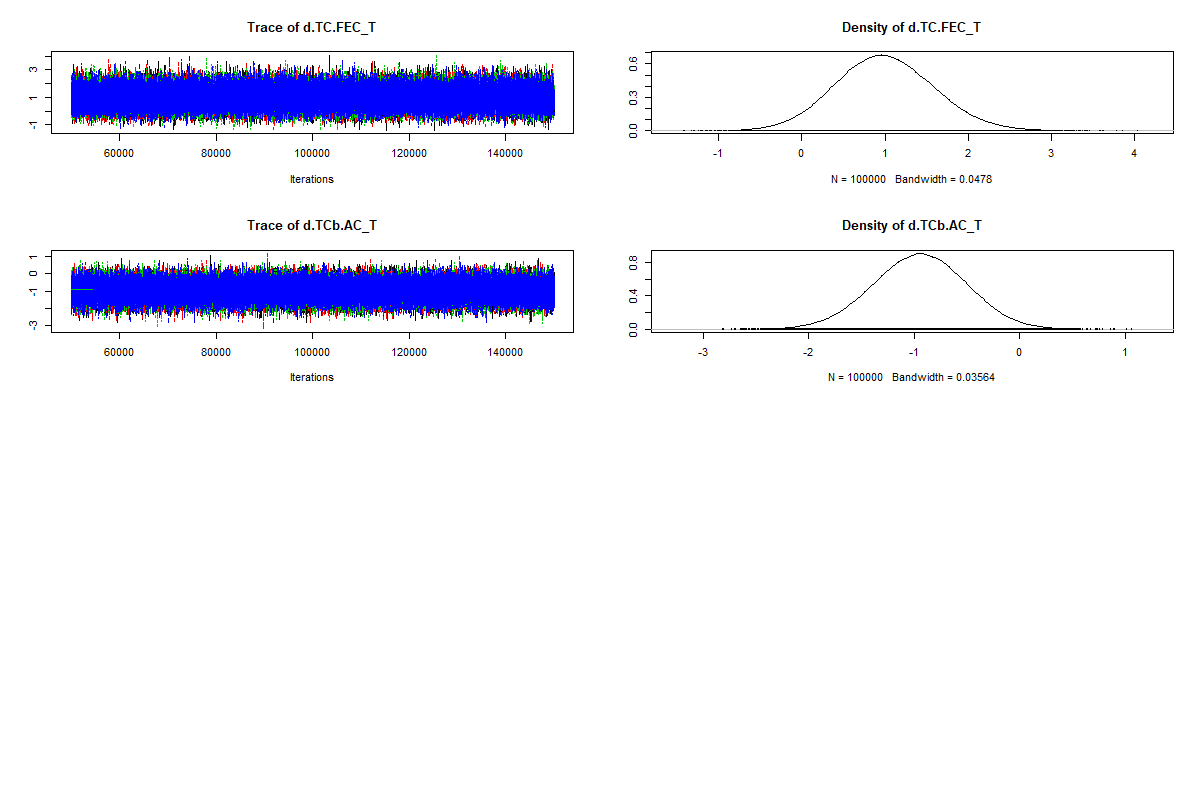


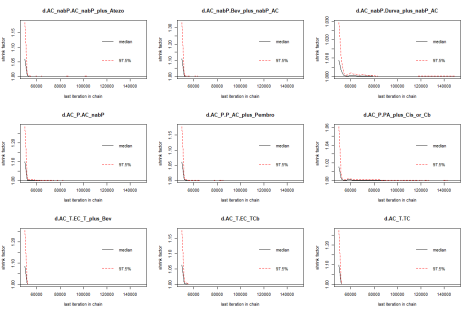

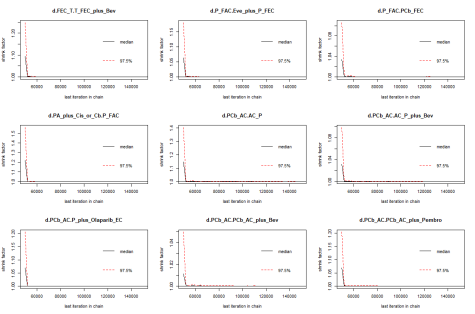

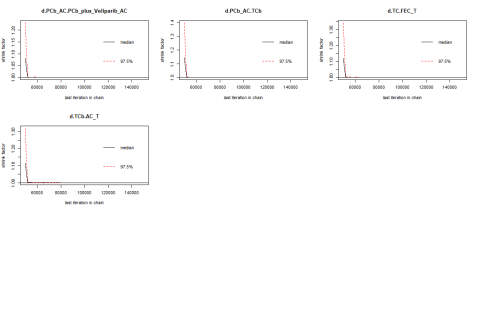


# Appendix 8 Results of NMA for aggregated AE (detailed regimens)

## Figure S12 Network plot

******

Notes: Direct comparisons are represented by the black lines connecting the neoadjuvant treatment regimens (NTR). Line width is proportional to the number of trials including every pair of NTR, whereas circle size is proportional to the total number of trials for each NTR in the network. A= doxorubicin; Bev=bevacizumab; C=cyclophosphamide; Cb=carboplatin; nabP=albumin paclitaxel (weekly cycle if not specially noted); P=paclitaxel (weekly cycle if not specially noted); Pembro=pembrolizumab; T=docetaxel; Veli=veliparib.

## Table S23 Number of Studies per treatment

| **AC_P** | **AC_P_plus_Bev** | **PCb_AC** |
| --- | --- | --- |
| 3 | 1 | 5 |
| **PCb_AC_plus_Bev** | **PCb_AC_plus_Pembro** | **PCb_plus_Veliparib_AC** |
| 1 | 1 | 2 |
| **TCb** |  |  |
| 1 |  |  |

## Table S24 Number of n-arm studies

| **2-arm** | **3-arm** | **4-arm** |
| --- | --- | --- |
| 2 | 2 | 1 |

## Table S25 Number of Studies per treatment comparison

| **Treatment 1** | **Treatment 2** | **Number of studies** |
| --- | --- | --- |
| AC_P | AC_P_plus_Bev | 1 |
| AC_P | PCb_AC | 3 |
| AC_P | PCb_AC_plus_Bev | 1 |
| AC_P | PCb_plus_Veliparib_AC | 2 |
| AC_P_plus_Bev | PCb_AC | 1 |
| AC_P_plus_Bev | PCb_AC_plus_Bev | 1 |
| PCb_AC | PCb_AC_plus_Bev | 1 |
| PCb_AC | PCb_AC_plus_Pembro | 1 |
| PCb_AC | PCb_plus_Veliparib_AC | 2 |
| PCb_AC | TCb | 1 |

## Table S26 Overall heterogeneity and consistency

|  | Overall *I^2^* | DIC | Differences of DIC [should less than 5] |
| --- | --- | --- | --- |
| Fixed effect model Consistency model | 51% | 37.59799 | 0.00754 |
| Fixed effect model Non-consistency model | 51% | 37.59045 | - |

Notes: Fixed-effect consistency model was performed for final analysis

## Table S27 NMA results

Notes: OR (95%Crl) reported. Statistically significant was presented as bolding format, green presents favour control, and red presents favour intervention

## Figure S13 Forest plot for all treatments compared with key regimens (AC-P)


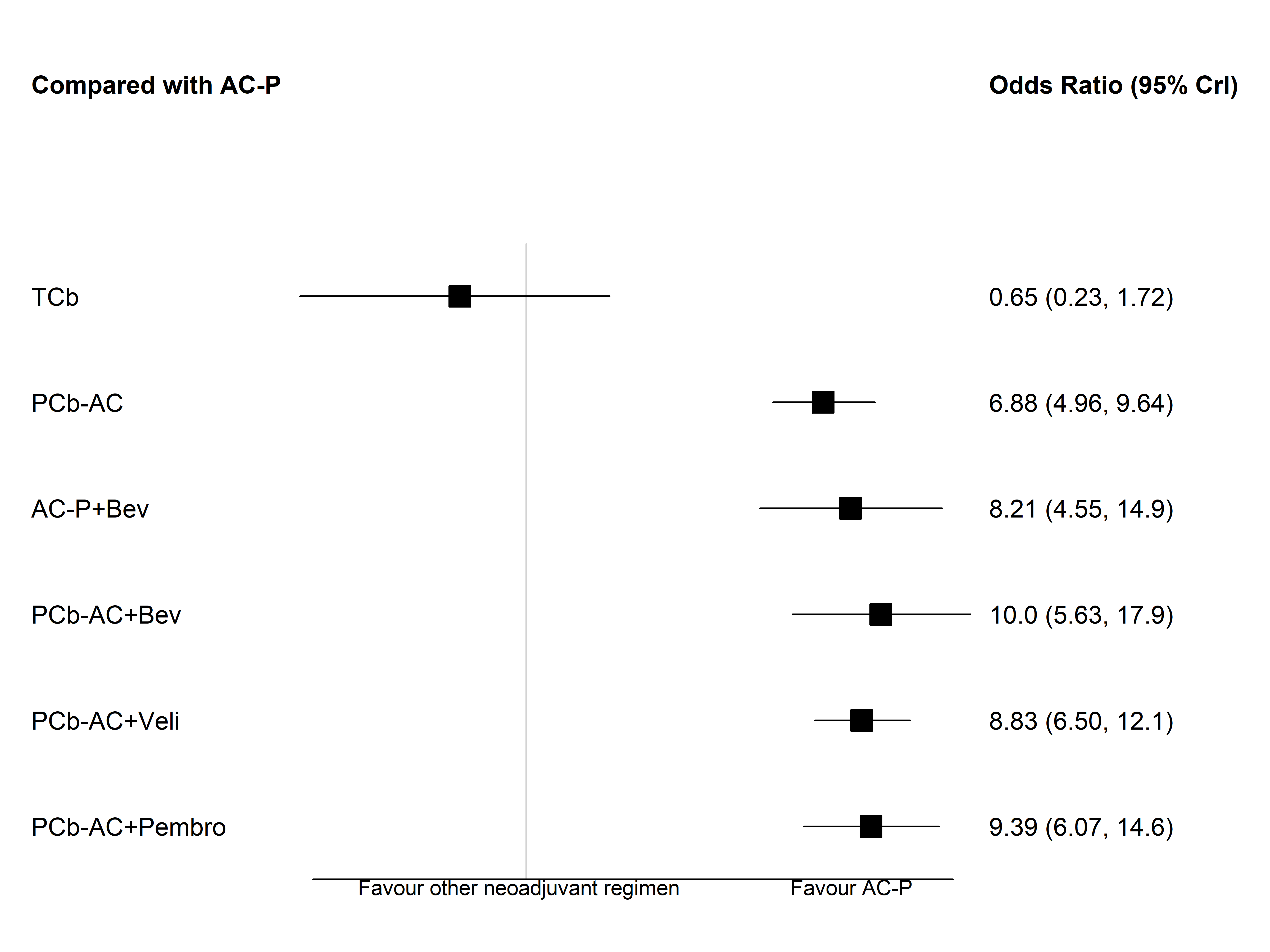


## Table S28 Ranking with SURCA

| **Ranks** | SUCRA |
| --- | --- |
| TCb | 0.9669100 |
| AC_P | 0.8664233 |
| PCb_AC | 0.5965058 |
| AC_P_plus_Bev | 0.3780200 |
| PCb_plus_Veliparib_AC | 0.2918388 |
| PCb_AC_plus_Pembro | 0.2218162 |
| PCb_AC_plus_Bev | 0.1784858 |

## Figure S14 Ranking plot


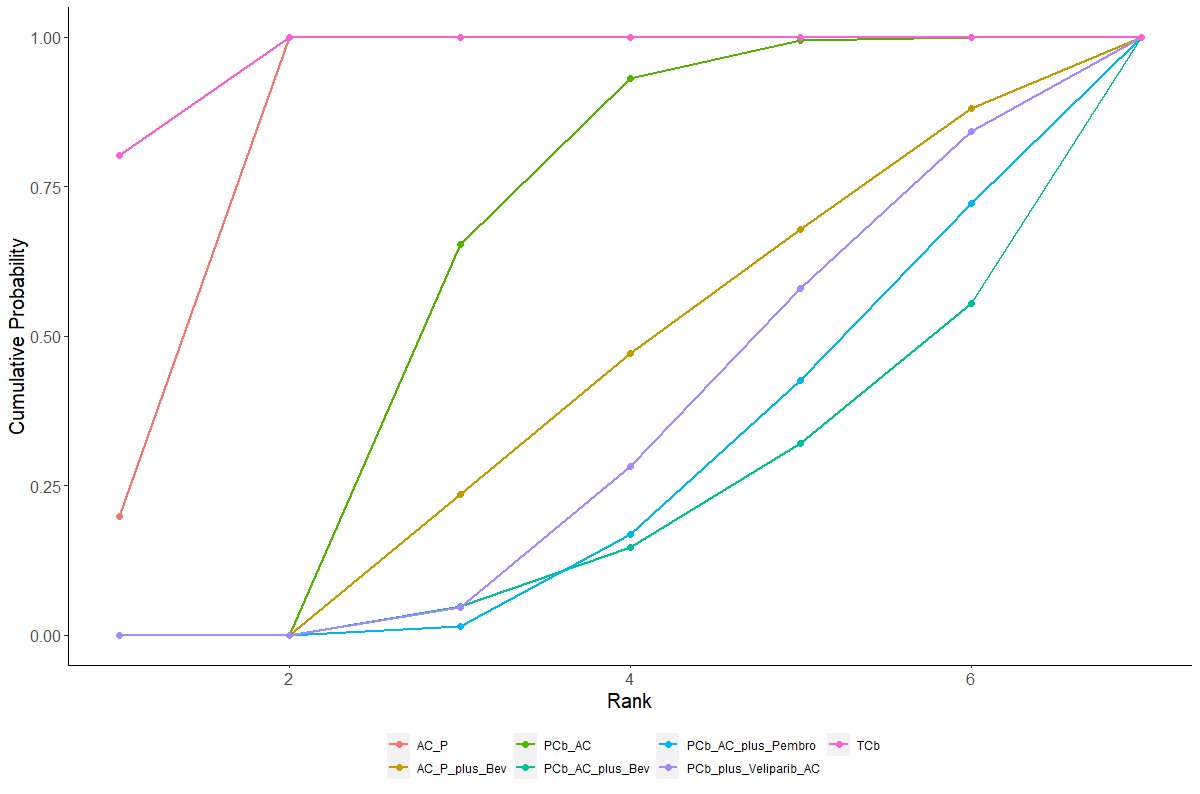


## Figure S15 Trace plot and density plot

PSRF=1.000163


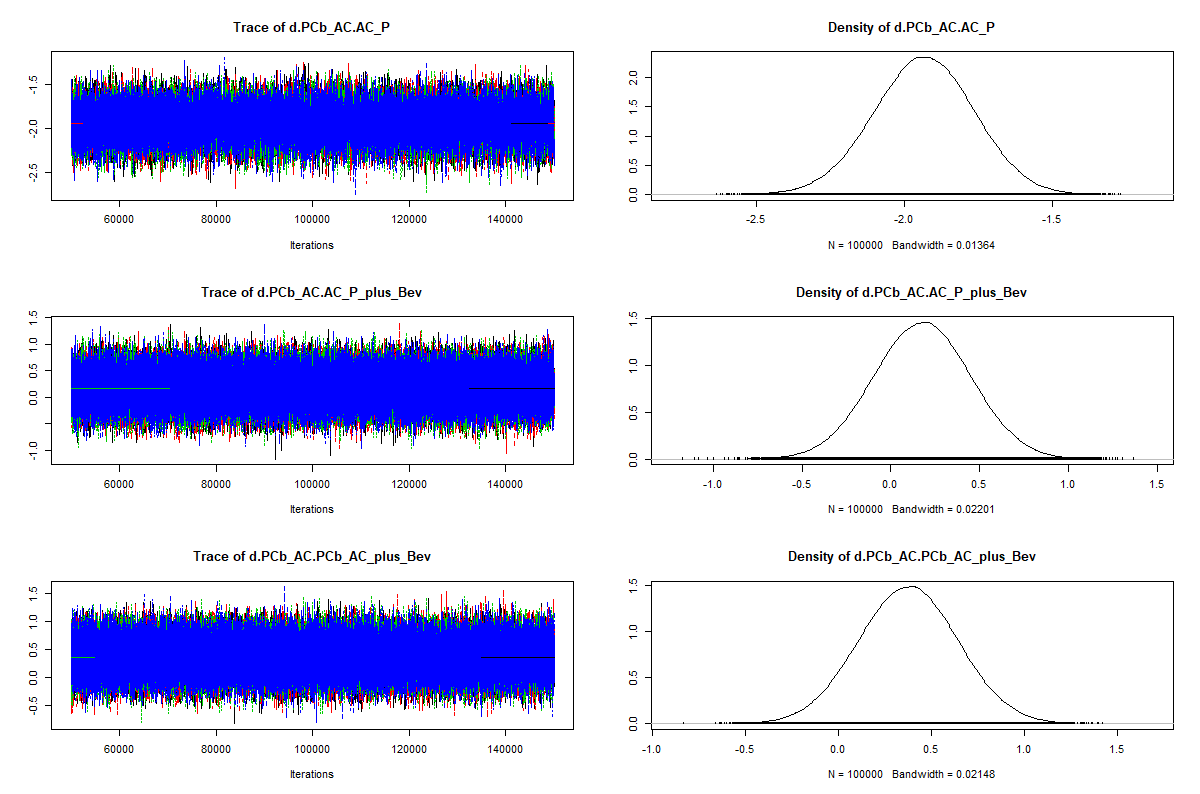

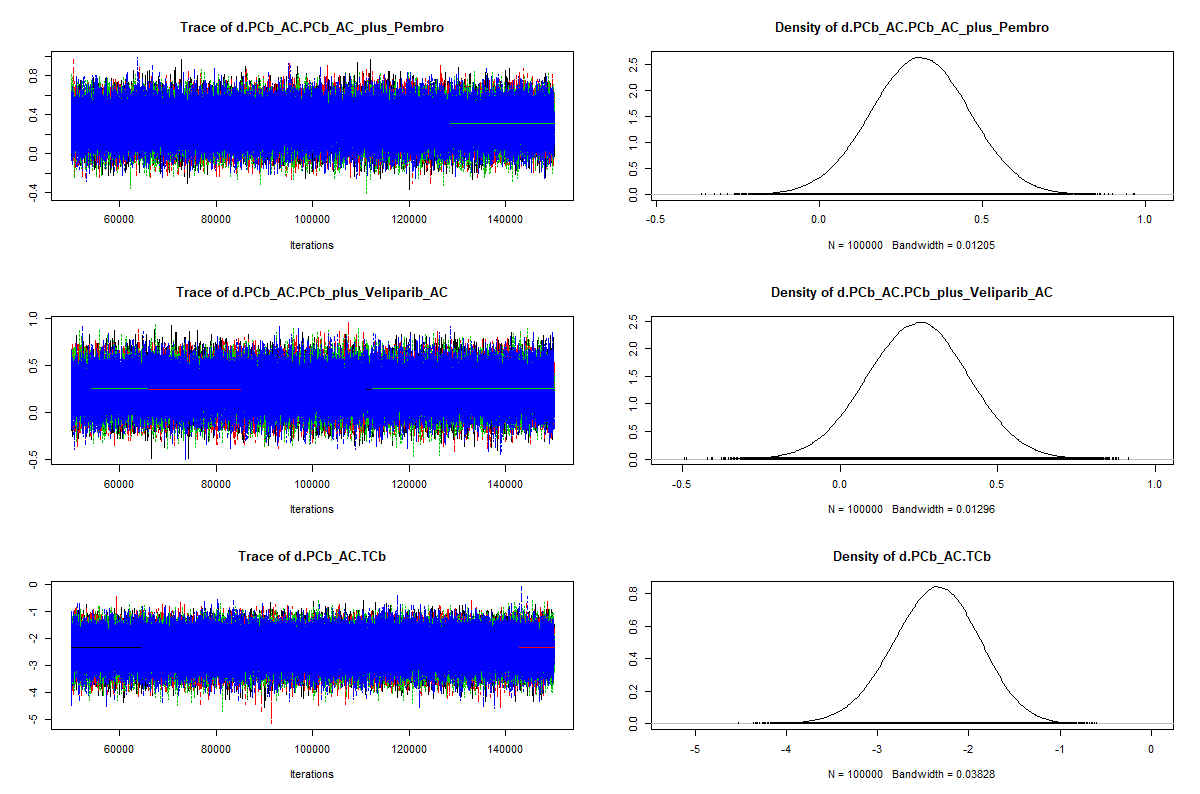


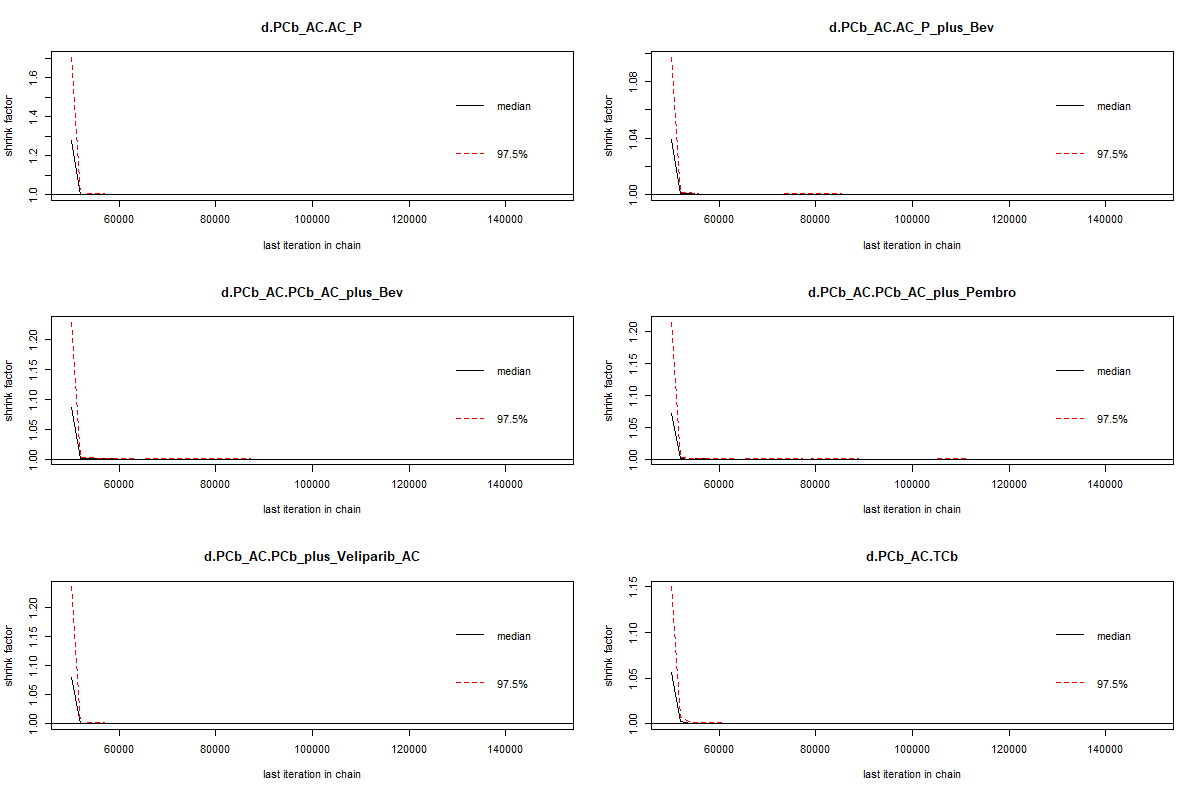

Supplement: Supplementary file 1 [file cbm-19-742-s001.docx]
